# Changes in Vertical Phenotypic Traits of Rice (*Oryza sativa* L.) Response to Water Stress

**DOI:** 10.3389/fpls.2022.942110

**Published:** 2022-07-14

**Authors:** Yufan Zhang, Yuanyuan Zha, Xiuliang Jin, Yu Wang, Han Qiao

**Affiliations:** ^1^State Key Laboratory of Water Resources and Hydropower Engineering Science, Wuhan University, Wuhan, China; ^2^Key Laboratory of Crop Physiology and Ecology, Institute of Crop Sciences, Chinese Academy of Agricultural Sciences, Ministry of Agriculture, Beijing, China

**Keywords:** drought response, drought-rehydration, image processing, vertical structural traits, rice vertical heterogeneity

## Abstract

Drought-rehydration irrigation has an enhancing impact on rice yield, but the current research on its yield-increasing effect is mainly experimental and empirical, lacking mechanism theoretical support. Image-based machine vision is rapidly developing and can estimate crop physical and chemical properties. A novel image processing method has been purposefully carried out to detect the real-time response shape of rice drought-rehydration. By application of this method, two new types of morphological descriptors were proposed to characterize and quantify the vertical phenotypic heterogeneity of rice, in which the relative height of the plant centroid (*RHC*) locates the growth focus, while the leaf angle distribution model describes the vertical characteristics of the leaf phenotypic traits. We verified the response of the vertical traits to different water treatments through designed experiments. The results showed that the *RHC* and leaf angle distribution parameters followed divergent trends under water stress, reflecting the drought characteristics of rice at different growth stages. The newly developed indicators were sensitive to drought response at specific growth stages and also efficient for evaluating rice growth, including determination of radiation interception capacity and assessment of nutrient accumulation. Furthermore, through the measurement and analysis of vertical structural traits, we found that a short-term water deficit and reasonable rehydration during the rice heading period could help to extend the spike-growing time and improve photosynthetic efficiency, thus benefiting yield formation.

## Introduction

As one of the most important grain food in China, rice contributes 31% of the total output of food crops by utilizing 26% of the cultivated acreage, thus rice plays a critical role in ensuring food security in China (Tester and Langridge, 2010; NBS, 2021a,b). Another aspect is that rice is more water-consuming and has low water and nitrogen use efficiency compared with other cereal crops (Bouman et al., 2007; Jin et al., 2016; Liang et al., 2021). To improve rice growth, the least costly and environmental-friendly initiative is the optimization of irrigation strategies, also known as water-saving irrigation (Mao, 2002). The alternating wet and dry pattern of water control improves water productivity and nitrogen utilization in rice, reinforces drought tolerance traits in intergenerational genetics, and ultimately increases yield and quality (Belder et al., 2004; Cui et al., 2004; Cheng et al., 2006; Thakur et al., 2014; Pei et al., 2015; Luo et al., 2019). Previous studies have shown that alternating drought and rehydration cultivation environments can cause significant changes in rice yield components including total grain number and seed setting rate (Gao et al., 2019). Intermittent water-saving irrigation can vary shoot vigor, reshape root traits, and lead to changes in N, P, and K uptake rates during the rice growth period (Huang et al., 2019, 2021). An appropriate level of water deficit has a facilitating effect on rice growth, but how to evaluate the intrinsic mechanism of this enhancing function and how to identify the optimal level of drought to avoid yield reduction are the main concerns of water-saving irrigation models for rice (Kang et al., 2021).

Phenotyping traits analysis is an important approach to study the water consumption of rice and analyze its adjustment regulation in coping with drought. It not only provides an efficient tool for the extraction of crop information but also establishes a cross-disciplinary bridge between genomics, environments, and agricultural management (Walter et al., 2015; Ji et al., 2018; Pieruschka and Schurr, 2019; Aasen et al., 2020). Previous studies showed that some phenotypic parameters on a plant scale such as a dark green color fraction, leaf morphology, and leaf size can effectively identify the occurrence of water stress (O’Toole and Cruz, 1980; Briglia et al., 2019; Cal et al., 2019). Since phenomics plays an important role in stress-related research on rice, it is critical to obtain morphological traits of rice and evaluate the response of phenotypic indicators. Accurate phenotype trait measurements require time-consuming and laborious manual work, and destructive sampling is prohibitive for continuous observation. Recently, the method based on digital image analysis seems to be an effective and reliable method for the measurement of rice phenotype. Image-based phenotyping measurement is not only about “taking pictures” but also about analyzing the interaction between plant pixels and light and shadow in the images (Li et al., 2014), which digitizes and extracts the response of rice to the real-world environments into the matrix of pixel cells. Several stress-sensitive visual indicators can be extracted from images of crops taken from a certain viewing angle or multiple dimensions (Moshou et al., 2014; Anshori et al., 2018; Laraswati et al., 2021). To determine the progression of senescence (maturity) and water deficit status of the plant quantitatively, some color index (CRI) is derived by partitioning pixels into green and non-green groups and calculating the color space characteristics. Besides, the analysis of the pixel number and area enables the estimation of the plant biomass ratio, which is useful for the assessment of plant growth status under stress (Petrozza et al., 2014; Malinowska et al., 2017; Zhuang et al., 2017; Mohan and Gupta, 2019). From a holistic perspective, image-based phenotypic traits that combine color, size, and structure can express richer information about crop physiology and are explanatory for the mechanism of crop response to water stress. As a representative study, Duan et al. (2018) propose four visual features from RGB images of individual rice, which are proved sensitive to the imposition of drought at different growth stages.

In addition to a single indicator, the combined analysis of multiple sources of phenotypic data can provide a more comprehensive description of the water physiological condition of the plant (Chen et al., 2014), which made more reliable and interpretable conclusions to assess the effect of rice environmental stress on phenotyping traits. In this paper, we developed a vertical distribution form of phenotypic traits to describe the overall rice (and possibly generalize to other crops) characteristics and its response to different water stress levels based on image visual technology.

The transfer and interception of photosynthetically active radiation (PAR) in the plant canopy is a top-to-bottom vertical movement, while the transport and absorption of nutrients and water in plant growth are dominated by a bottom-to-top vertical process. Thereby, the physiological and morphological traits of crops form distributional differences in the vertical direction (Song et al., 1997). The heterogeneity mutates following certain regulations under the stress of the external environment as well. It was shown that the chlorophyll content of the plant is unevenly spread over the vertical profile as influenced by the internal light distribution in the canopy, which could be expressed by the spectral reflectance (Barton, 2001; Ciganda et al., 2008). Likewise, the leaf nitrogen content (LNC) in the horizontal layers varies across different heights of the canopy, and the response of LNC to soil nutrients also differs at different nutrient stress levels (He et al., 2020). Externally, the vertical morphology of congeneric crops has similar genetic attributes and specific patterns. Some structural indicators, including leaf size, leaf angle, branch number, etc., conform to certain distribution formulas. Information regarding leaf area (*LA*) index vertical distribution is helpful to analyze the effect of canopy structure on photosynthetic ability and dry matter accumulation (Hirooka et al., 2018). Suitable vertical heterogeneity distribution of *LA* optimizes light transmission path, improves *PAR* interception efficiency, and enhances aeration at the canopy levels. Moreover, it notably avoids oversaturation of radiation in the upper part of the plant and undersaturation in the lower part. Phenotypic heterogeneity of high-yield varieties has high heritability and can be a dominant morphological trait in generational selection (Li and Wang, 2013; Perez et al., 2019). The existing problem is how to qualify the phenotypic vertical heterogeneity of plants, in the form of a single index or expression with several parameters. The practice was pioneered in the field of forestry. Branch, crown vertical *LA* distribution models have been developed for the broadleaf forest to find species-specific differences based on right-truncated Weibull distribution models (Nelson et al., 2014). Studies on maize used a simplified form of the bell-shaped function to describe the relationship between leaf rank and *LA* (Fan et al., 2020).

However, there were few studies on the vertical phenotype of rice response to environmental stress. On the one hand, since the single plant morphological traits of rice are complex with a large number of tillers and leaves, it is difficult to generalize the characteristics from digital images. On the other hand, data were scarce for research on the vertical phenotype of rice due to the time-consuming and intermittent drawbacks of traditional observation methods. Therefore, in this paper, we proposed a simple and efficient visual analyzing method for the study of phenotypic traits, which combined a computer vision algorithm with vertical phenotyping analysis. By designing experiments with different water stress levels, this paper aimed to understand the response of rice vertical phenotypic distribution traits to changing soil water conditions. The results of the experimental analysis can be applied to interpret the physiological functions of vertical morphology of rice under changing water conditions in soil and thus understand the adjustments made in appearance morphology when rice encounters external stresses. The main objectives of the paper were to (1) develop a novel image processing and data interpretation method for analyzing the vertical heterogeneity of rice canopy, (2) propose several statistical indicators to quantitatively represent the vertical distribution of structural phenotypes and identify the stress-sensitive factors involved, and (3) compare the correlation between the proposed indicators and the rice growth status and evaluate their indication capacity of the effect of water stress.

## Experiment and Materials

### Experimental Site and Setting Overview

Experiments with different water stress levels were conducted at the Irrigation and Drainage Experimental Site of State Key Laboratory of Water Resources and Hydropower Engineering Science, Wuhan University (114°37′E, 30°54′N). The rice seedlings were cultivated in Jingmen, Hubei, on 31 May 2021, and transplanted in experimental pots on 1 June, which have an inner diameter of 20 cm and a depth of 25.5 cm. Two rice seedlings were transplanted into each pot. The rice for experiments was a kind of indica two-series hybrid rice, with a seedling age of about 30 days and a total fertility period of about 140 days. The pots were filled with 6.4 kg of air-dried soil that passed through a 2-mm sieve, and the measured soil bulk density was about 1.2 g.cm^3^. In this study, we artificially regulated the degree of water stress by controlling the soil moisture content (*SMC*). Before the experiments, the soil retention curve was fitted by measuring soil water potential and SMC at different soil saturation levels. In addition, the parameters of soil for experimental cultivation were provided in Supplementary Table 2.

### The Experiment of Water Stress

By design, the soil water potential subjected to mild drought stress was maintained between -20 and -10 kPa (80% of field capacity), while severe drought stress was between -35 and -25 kPa (60% of field capacity). We monitored *SMC* roughly by weighing the pots every half-day and determined the current soil water potential value based on the soil water retention curve measured in advance. When observing that the negative soil pressure dropped to the lower limit of the design, we replenished the appropriate amount of water to keep the predesigned soil water potential.

During the whole growth period of rice, we designed three water treatment groups, one for flood irrigation treatment (WF), i.e., always keeping a shallow water layer of 5–50 mm, one for mild drought stress (WM), and another group was for severe drought stress (WS). The drought process started from the 25th day after transplanting (25th June) until maturity for harvest.

**FIGURE 1:**
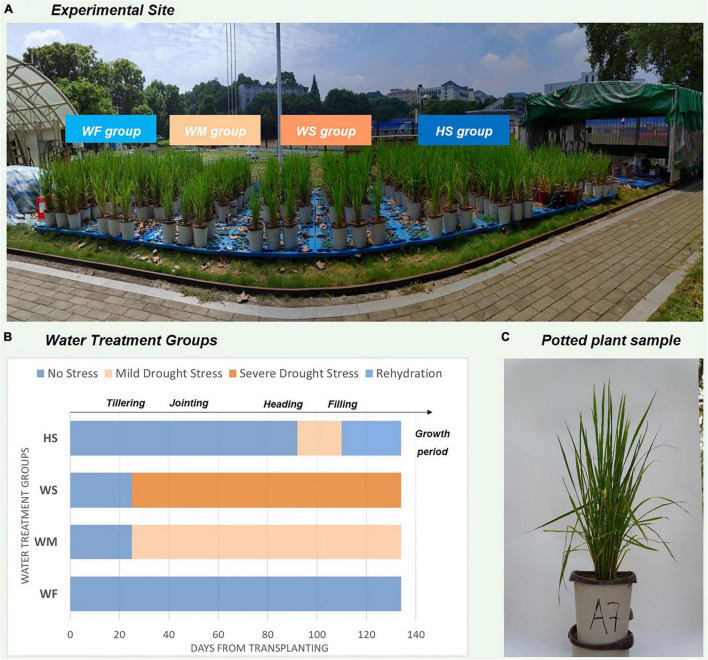
Description of the experiment conducted and the water treatment process. **(A)** Experimental site, **(B)** The degree and duration of water stress in different water treatment groups. The *x*-axis number refers to time-elapsed from rice transplanting (Jun 1st), **(C)** Plant sample of potted rice.

To understand the pattern of water consumption and response to water stress in rice at different growth stages, we set up drought treatments at the heading stage (HS) specifically. After being observed to have entered the specified growth period, the potted plants began to drain and dry until *SMC* was reduced to a set level and resumed flood irrigation by the end of the period. Figure 1 showed an overview of the site where the experiment was conducted and how the images of rice samples were obtained. The periodic water treatments for the four groups are illustrated in Figure 1B, and the details about the experimental design were provided in Supplementary Table 1.

## Materials and Methods

### Data Acquisition and Processing

At each sampling date, we examined various physiological and phenotypic indicators of potted rice samples, including plant height (*PH*; measured by tapeline), PAR (measured by handle radiometer), and *LA* (measured by green *LA* scanner), biomass, and *SMC*, etc. Front-view images of potted planting rice were captured using a SONY a7m3 digital SLR camera with an exposure value set to 0. The shooting distance was 1.2 m ± 0.1 m. Each potted sample was oriented with the widest side (most extension side) facing toward the camera imaging side (target side). All the image thumbnails of the potted plants were provided in the Supplementary Image Series.

**FIGURE 2:**
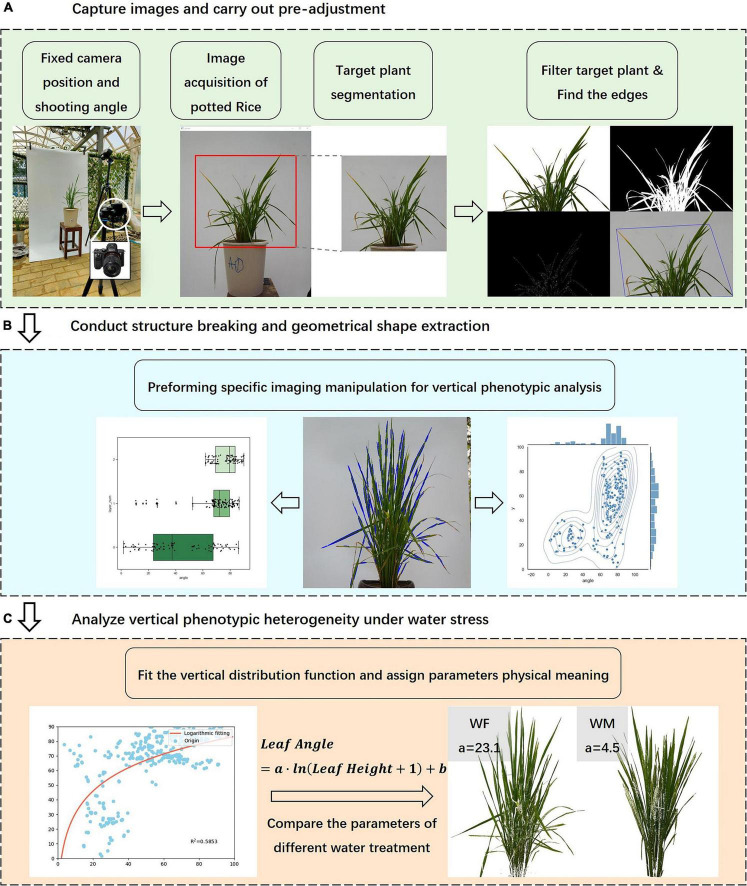
Experimental procedure and image data analysis process. **(A)** Acquisition and pre- processing of rice front- view images, **(B)** Extraction of the geometry of rice subjects, **(C)** Analysis of the characteristics of vertical traits.

After the data collection and image acquisition work, the rice images were processed according to the following steps, including the calculation of the vertical phenotypic traits distributions, and the analysis of the variability of traits and the correlation between each other (Figure 2).

(A) The pre-processing of rice images included region of interest cropping, rice subject identification and segmentation, edge detection, contour depiction, external convex hull extraction, etc. The purpose was to avoid the interference of background and clutter and to emphasize the geometric structures of the plant. The segmentation of the plant was completed by color thresholding. The cropped image was transformed to HSV color space which is more conducive to identifying green plants, and then background pixels and rice pixels were discriminated using set coupled thresholds. The boundary lines of the plant were extracted using a Canny edge detector (Thanikkal et al., 2018), and the bounding polygon was portrayed by Harris detection and the Convexhull algorithm (Hamuda et al., 2018; Wang et al., 2019).
(B) In this step, the centroid of the plant contour was located, which determined the pixel coordinates of the plant center. The plant edges were turned into line segments by the Hough transform algorithm, and the inclination of the lines was consistent with those of the leaves. Through the above operation, the plant structural morphology was reduced to a relatively simple geometric trait, preserving the three-dimensional features and facilitating the subsequent phenotypic traits calculations.
(C) Finally, we computed several phenotypic traits based on image processing results and fitted the vertical distribution function of leaf inclination angle, attributing physical significance to the parameters. These traits reflected the vertical characteristics of rice, and we were to study the physiological mechanisms of the crop under water stress by analyzing the correlation of the characteristics with rice growth status.

### Phenotypic Indicators Measurement and Calculation

As with traditional phenotyping research, we measured a series of rice phenotypic traits to monitor the changes under different water stress conditions. Besides, based on digital images and computer vision techniques, we innovatively proposed independent traits and a fitting function to characterize the vertical heterogeneity of rice phenotyping traits.

In Supplementary Table 3, some of the traits have been rather maturely explored in previous studies, and while we focused on such conventional features, we proposed novel traits for analysis, which proved more efficient in indicating the growth status of rice. In addition, the raw data of the observed traits mentioned in Supplementary Table 3 were provided in Supplementary Table 4.

The independent vertical feature was the relative height of the plant centroid (*RHC*), which means the ratio of the distance from the center of mass of the plant to the ground to the whole *PH*. The definition and calculation of *RHC* are provided in Eq. (1).


(1) $$RHC\;=\;\frac{Centroid\;Height}{Plant\;Height}$$

**FIGURE 3:**
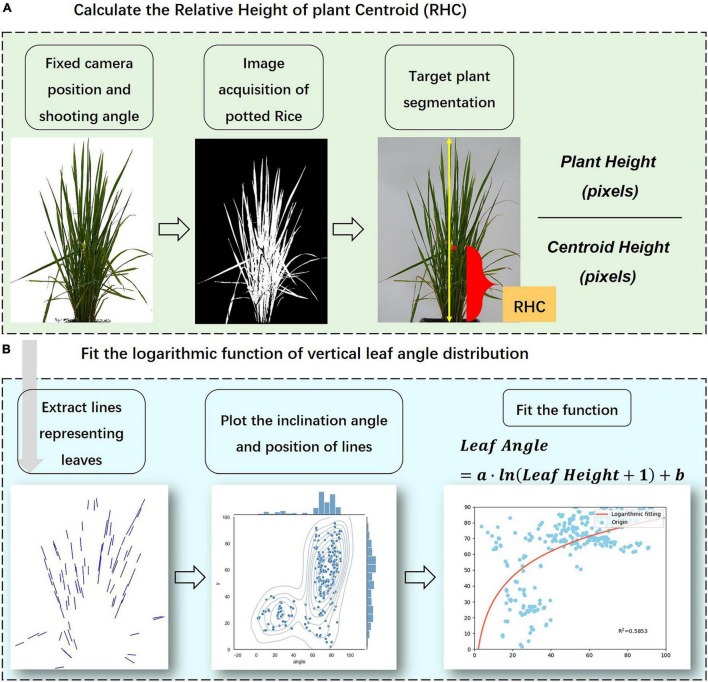
Calculation of the visual indicators according to image processing results. **(A)** Calculation of *RHC*, **(B)** Fitting of vertical distribution model.

In Eq. (1), both *Centroid Height* and *Plant Height* refer to the pixel size in the front-view image. First, the topmost and bottommost pixel points were retrieved using the filtering algorithm, and the vertical distance between the top and bottom pixels was figured out as the *Plant Height* (excluding drooping leaves pixels). Second, the segmented plant was transformed into a binary image mask, and the moments algorithm was used to find out the shape center position. The vertical pixel distance between the centroid and the bottommost pixel was used as the *Centroid Height*. Finally, the ratio of the two was defined as the *RHC*. Figure 3A illustrated the procedures to calculate *RHC* from plant images.

### Vertical Distribution of Leaf Angle

**FIGURE 4:**
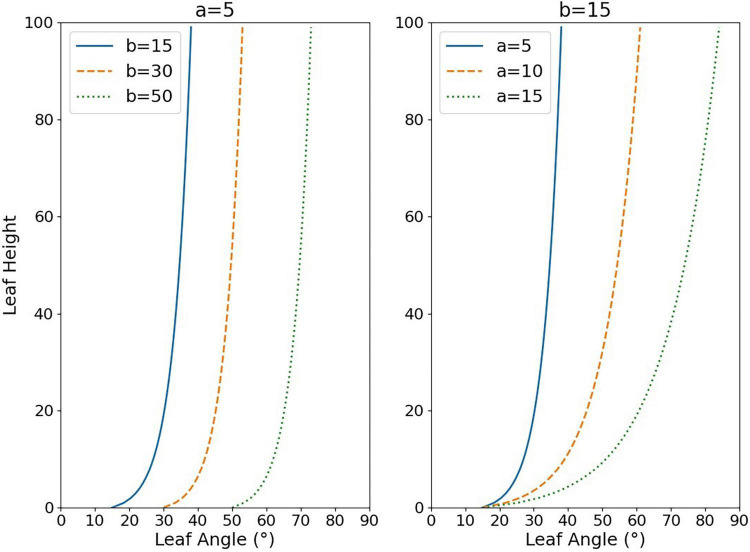
Effect of changes in the values of parameters *a* and *b* on the fitted logarithmic function curve shape. The *y*-axis represents the relative height of the leaf (*RH*_*l*_), while the *x*-axis represents the leaf inclination angle at that height position (*A*_*l*_).

Leaf angle is defined as the angle between the normal of the leaf outer surface and the zenith direction, numerically equal to the angle between the leaf surface and the horizontal plane (Campbell, 1990; Campbell and Norman, 1990). In this paper, we not only calculated the average angle of rice leaves (*ALA*) but also established the corresponding equation for the leaf angle and the leaf position to quantitatively describe the continuous vertical phenotypic characteristics of rice plants. There are two main reasons for using leaf angle as the object of research for the vertical phenotypic distribution: (1) Angular features are scaled projection invariant, so the value of leaf angle can be calculated from the front-view images precisely and non-destructively. (2) Leaf angle is one of the key traits expressing plant shape of rice, which not only directly determines leaf light exposure area to affect the interception of radiation by the canopy but also has a high contribution to rice yield (Mantilla-Perez and Salas Fernandez, 2017; Hu et al., 2019). According to the performance of experimental data, the vertical distribution of rice leaf angle showed a trend of increasing from bottom to top, and the increase slows down with the rise of relative height. This variation feature is similar to the shape of the logarithmic curve, so this paper tried to use the logarithmic function as the fitting form to describe the vertical traits of the leaf angle. Figure 3B displays the flowchart to fit the vertical distribution function of leaf angle. The data pair of leaf angle combined with its relative height was obtained and then fitted by the non-linear least squares method, obtaining the logarithmic function as shown in Eqs (2, 3):


(2) $${\text{A}}_{l}=a\cdot \text{ln}\,\left(R\,H_{l}+1\right)+b$$

(3) $$R\,H_{l}=\frac{H_{l}}{P\,H}\times 100$$

where *A*_*l*_ refers to the inclination angle of the leaf line, *RH*_*l*_ refers to the relative leaf height which can be calculated by Eq. (3), and *H*_*l*_ refers to the distance of the midpoint of the line representing the leaf from the bottommost, *PH* refers to the plant height, *a* and *b* in Eq. (2) are fitting parameters influencing the shape of the curve (Figure 4). The logarithmic model is the result of a scatter fit to the leaf angle data, and the independent variable is the relative height indicating the leaf position, taking value in the range of 0–100, which is used to estimate leaf angle at different heights of the plant. First, the inclination angle of the line segments representing the leaves was calculated by image processing and skeleton extraction algorithm. These lines were retrieved by the Hough line-detection algorithm. Second, the midpoint coordinate of each line segment was considered as the value characterizing the relative vertical position of the leaf, and the relative distance to the bottommost in relation to the whole plant was computed as the *RH*_*l*_. Finally, after completing the calculation of *A*_*l*_ and *RH*_*l*_, the paired data sets were fitted with the curve-fitting algorithm to obtain the logarithmic model parameters *a* and *b*. According to the calculation, the range of values of *a* was 0–20 and the range of values of *b* was -20 to 90 at most growth stages, thus Figure 4 shows the effects of *a* and *b* on the distribution shape. The value of *a* affects the rising (falling) trend of the logarithmic curve, while the value of *b* mainly controls the starting point of the curve (when *RH_*l*_* = 0). In this paper, we analyzed the response pattern of the vertical distribution model to different water stress levels, and the indication of plant growth, to verify the model’s performance in describing the vertical morphological heterogeneity of rice.

### Statistical Analysis

In the analysis of experimental data, the accuracy of the phenotypic traits calculated using the computer vision method was verified by the Pearson correlation coefficient analysis, and the root-mean-square error compared with the manually measured values was calculated. The parameters *a* and *b* in Eq. (2) were fitted using non-linear regression analysis, and the correlation coefficient was used to evaluate the accuracy.

To test the response of physiological and morphological expressions to different water treatments, the one-way analysis of variance (ANOVA) with *post hoc* comparison using Tukey’s HSD method was applied at a 95% confidence level. In parallel, multivariate linear regression (MLR) was employed to analyze the performances of different traits combinations to indicate the growth and radiation interception capacity of rice.

The concrete results of statistical analysis in this study were provided in Supplementary Tables 5–7.

## Results

### Variation in Phenotyping Traits Under Long-Term Water Stress

**FIGURE 5:**
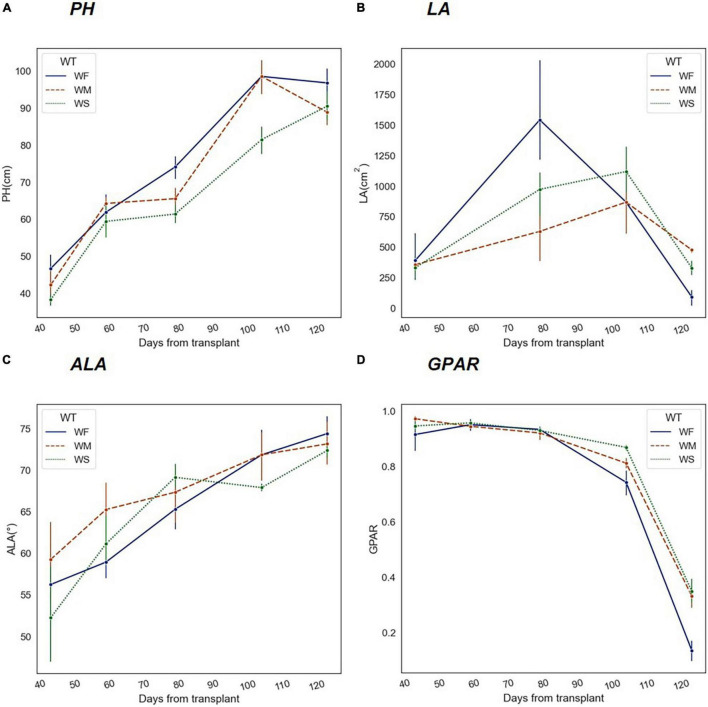
Trends of phenotypic traits in different water treatment groups (WF, WM, and WS) during the whole growth period. The *x*-axis represents the number of days experienced after rice transplanting. **(A)** Plant height (*PH*), **(B)** Leaf area (*LA*), **(C)** Average leaf angle (*ALA*), and **(D)** Greenness ratio (*GPAR*).

Under normal irrigation conditions (WF), both *PH* and average leaf angle (*ALA*) increased during the maturation of rice, while *LA* went through a process of increasing and then decreasing, as the green leaves dropped significantly after the panicles were tasseled. As shown in Figure 5, the temporal changes of *PH*, *LA*, and *ALA* were all affected by long-term water stress to different degrees, presenting regular variations. During long-term water stress, *PH* was always at a lower level (Figure 5A). Second, *LA* was significantly lower at the increasing stage and declined more slowly at the decreasing stage (Figure 5B). Third, *ALA* was higher at the early stage but by the end of the HS, the differences were not significant (Figure 5C). Moreover, Figure 5D showed that the greenness of rice declined continuously with growth.

### Variation in Vertical Phenotyping Traits Under Long-Term Water Stress

**FIGURE 6:**
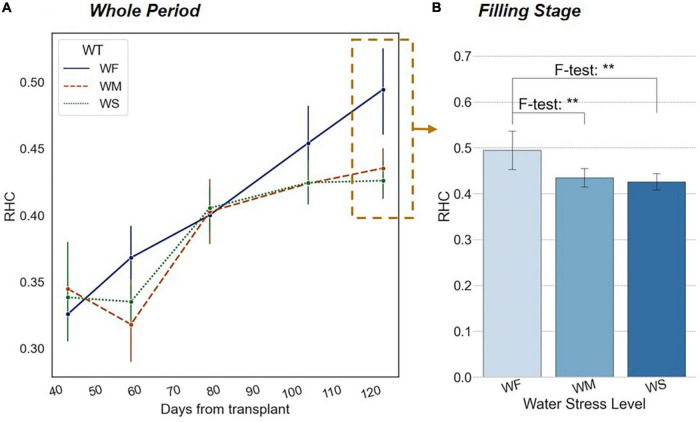
**(A)** Trends of *RHC* in different long-term water treatment groups during the whole growth period. **(B)** Significant differences in *RHC* among three water treatment groups at the filling stage. The *x*-axis represents water treatment groups. The blue bars indicate the value of *RHC*. The number of asterisk (*) represents the degree of the significance (*p*-value of < 0.05 *, *p*-value of < 0.01 **, and *p*-value of < 0.001 ***).

As shown in Figure 6A, *RHC* continuously increased during the whole growth period. After transplanting, the growing focus of rice shifted from underground to the surface when the root system was well developed. With the accumulation of aboveground biomass, the geometric center (i.e., the centroid) of the plant also rose. At the heading and filling stages, the increasing rate of *RHC* accelerated evidently until the final yield was formed.

According to the results of the experimental groups WM and WS in Figure 6A, the increasing trend of *RHC* was significantly suppressed under long-term water stress. Mainly during the yield formation period, the process of rice heading, fruiting, and filling was notably delayed by water stress, thus the corresponding *RHC* was lower. The differences were significant in the observation at the reproductive growth stage (i.e., filling and mike-riping stage) based on a one-way ANOVA result, while multiple *post hoc* tests proved that the *RHC* values of the WF group were significantly higher than those of WM and WS groups, but the difference between WM and WS groups was not significant (Figure 6B).

It can be illustrated that long-term stress caused the decrease in the panicles number and seed setting rate, which led to the reduction in yield. The effect on rice external morphology showed a decrease in the growth rate of *RHC*. In contrast, during the period when nutrient accumulation was mainly carried out (i.e., tillering and jointing stage), we noticed a significant change in the vertical heterogeneity characteristics of the rice plants.

**FIGURE 7:**
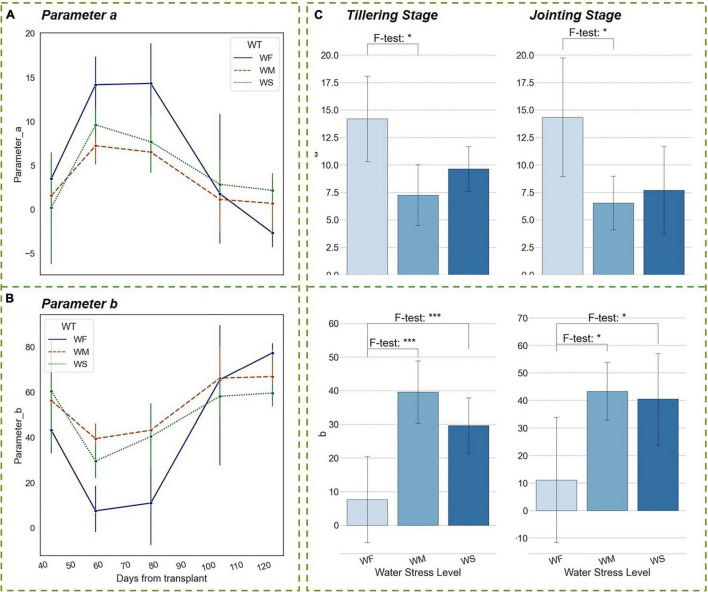
Trends of parameters of the vertical distribution function in different water treatment groups during the whole growth period. **(A)** Parameter *a*, **(B)** Parameter *b*. **(C)** Significant differences in parameters among three water treatment groups at the tillering and jointing stages. The blue bars indicate the value of parameter a or b. The number of asterisk (*) represents the degree of the significance (*p*-value of < 0.05 *, *p*-value of < 0.01 **, and *p*-value of < 0.001 ***).

As shown in Figures 7A,B, after the rice tillering process, the spreading degree of the rice plant reached its maximum. During this period, the plant had a small leaf inclination angle, an extended *LA*, and a high *PH* growing rate. The upper leaves of the plant were biased erect and the middle and bottom leaves tended to spread out, forming an optimized shape that was narrow at the top and wide at the bottom. The above-mentioned vertical distribution pattern was conducive to radiation interception and light absorption, as well as to increasing the diurnal temperature difference and enhancing aeration in the rice canopy (Wang and Li, 2005; Springer, 2010; Xiao et al., 2021). The dashed lines in Figures 7A,B represented the results of the experimental groups WM and WS. Parameter *a* under long-term water stress reached its maximum at the end of the tillering stage as well, but this maximal value was much smaller than that of WF groups. The differences were proved to be significant in the ANOVA results and persisted until the HS (about 100 days after transplanting). Parameter *b* had a similar pattern as *a*. The statistical analysis results showed that the vertical heterogeneity of leaf angle was significantly affected by water stress when the rice plants were in the nutrient accumulation period of tillering and jointing within 50–100 days after transplanting (Figure 7C). It was thus clear that long-term water stress has shaped a more compact morphology of rice, and the vertical heterogeneity was lower.

### Changing Trends in Vertical Phenotypes Under Short-Term Water Stress at Heading Stage

In actual agricultural production, short-term drought is a relatively common stress situation. To verify the response pattern of the vertical phenotypic traits that we proposed to short-term water stress, a specific type of experimental group was implemented at the HS (Figure 1B).

**FIGURE 8:**
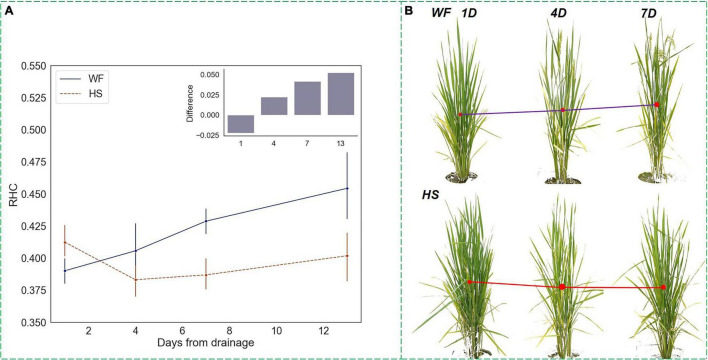
Changing trends of *RHC* under short-term water stress at the heading stage. **(A)** RHC trends and differences. **(B)** Changes in the front-view images of rice 7 days after drought. The blue line (upper) represents the *RHC* of plants in the WF group, while the red line (lower) represents the *RHC* of plants in the HS group. The *y*-axis of the histogram represents the difference between *RHC* of the WF group minus that of the HS group.

The HS is a critical period for rice yield formation during which the rice panicle begin to emerge from the sheath until completely withdrawn (Meier, 2001). Therefore, changes in rice phenotype during this stage could visually interpret the effect of water treatments on rice growth status. Functional leaves remain erect, while ineffective tillers begin to wither, and non-functional leaves stretch or shrivel. During this period, the accumulation of above-ground biomass accelerated, and therefore the increasing rate of *RHC* rose, indicating that the growth focus of rice shifted upward to the panicles. When plants were subjected to short-term water stress at the HS, as shown in Figure 8, *RHC* values experienced a significant decrease within 1–4 days of drought onset and then continued to increase tardily after 4 days, but produced differences from normal levels, which were proved significant by the ANOVA test. This result indicated that short-term water stress leads to a lower growth focus during the rice heading process. In terms of plant physiology, the short-term drought delayed the emergence of rice panicles, and this effect prolonged the process of nutrient accumulation in rice, which improved the final yield. This was similar to the variation model of *RHC* in rice under long-term drought, and there was a regular response pattern of the *RHC* indicator for both long-term and short-term water stress, which suggested that *RHC* is a drought-sensitive visual feature with certain physical interpretability.

**FIGURE 9:**
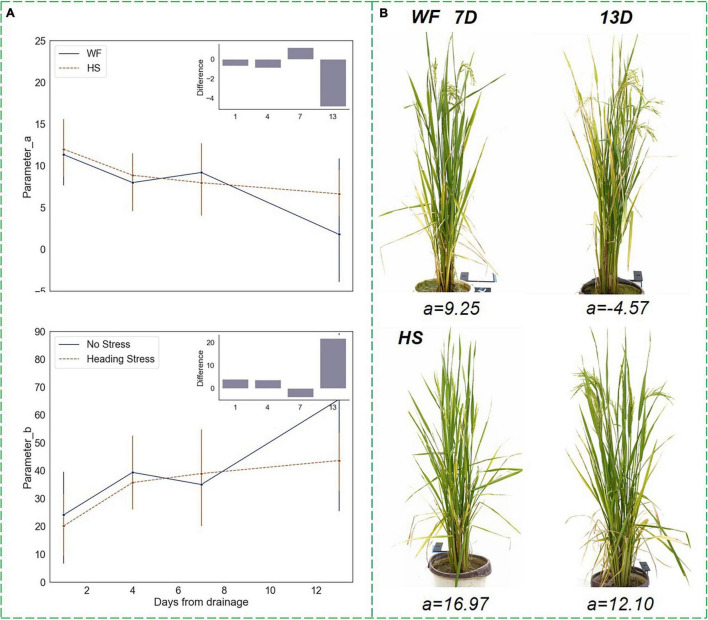
Changing trends of parameters in the vertical distribution function under short-term water stress during the heading stage. **(A)** Parameters *a* and *b* trends. **(B)** Changes in the front-view image within 7–13 days after drought, where the upper images showed the plants in the WF group, and the lower images showed the plants in the HS group.

Compared to the *RHC*, parameters *a* and *b* had less drought sensitivity but were significantly discriminated under short-term water stress as well. As shown in Figure 9, at the HS, parameter *a* kept decreasing in the WF group, while parameter *b* kept increasing, indicating that the vertical phenotypic heterogeneity would diminish during the rice heading process. The degree of leaf spreading weakened, the difference in leaf angle between the top and bottom layers narrowed, and the plant morphological shape became more compact. However, after being subjected to mild water stress, the downtrend (uptrend) of parameters *a* and *b* became flatter, and the differences from the value of the WF group reached a significant degree when the drought proceeded to day 13, which was proved by the ANOVA test that *p*-value of < 0.05. Examples of front-view images of rice samples under different water treatment conditions are shown in Figure 9B. It can be seen that during the 7 days of drought lasted, parameter *a* was positive and the differences between the two experimental groups were not significant. However, on the 13th day of drought, when the plants in the WF group had a higher degree of tasseling, *a* decreased to a negative value, and the HS group maintained a higher value of *a*. As can be seen in the sampling images, the plants in the WF group were significantly more compact and the lower leaves were almost non-existent, while the plants of the HS group retained some of the lower leaves and had a more spread morphology. In conclusion, the results indicated that the short-term drought at the HS maintained a high heterogeneity in rice vertical morphology rather than causing it to diminish rapidly.

The response patterns of the vertical leaf angle distribution function to long-term and short-term drought were different. In the model of long-term water stress, the differences in the vertical distribution function parameters mainly occurred at the tillering and jointing stages, and largely disappeared after heading, indicating that prolonged drought mostly affected the morphological traits of the plants during the nutrient accumulation period and eventually impacted the yield, while the effects of short-term drought occurring during the reproductive growth period on the vertical phenotypes were relatively lagging and insignificant.

### Phenotypic Changing in Rice During Short-Term Drought and Rehydration

After the effects of drought are detected by visual phenotypic analysis, compensatory rehydration irrigation is usually performed in agricultural production. In our experimental design, after a period of water stress lasting at the rice HS, flood irrigation was applied on day 19 of the drought to restore *SMC* to the field capacity rate. The *SMC* of the HS group was consistent with the WF group until the 32nd day from the drainage, and the rehydration lasted 14 days. This drainage-rehydration operation spanned the heading and filling stages and was completed at the milk-ripe stage.

**FIGURE 10:**
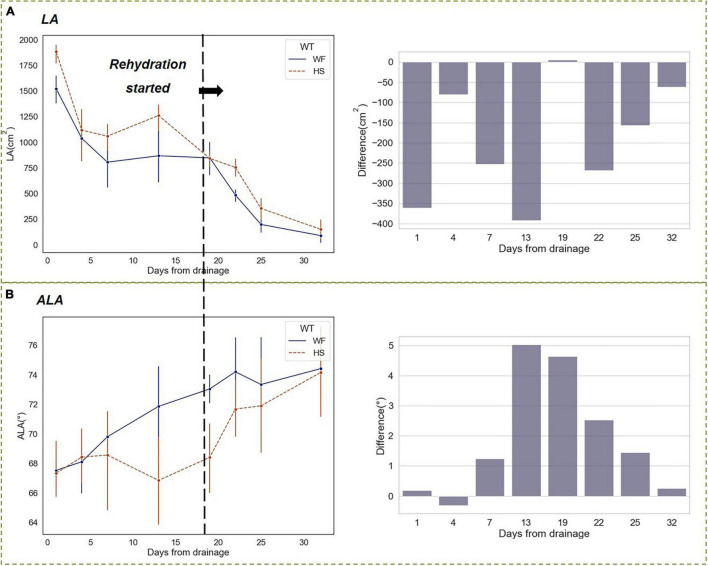
Changing trends of phenotypic traits under short-term drought-rehydration at heading and filling stages. **(A)** Leaf area (*LA*). **(B)** Average leaf angle (*ALA*).

Figure 10 showed the results of changing trends of leaf phenotyping traits. During the reproductive growth period when the *LA* began to decline, short-term water stress attenuated the decreasing trend, resulting in a higher *LA* in the HS group than that in the WF group. In contrast, the advantage in *LA* disappeared on the first day after resuming flood irrigation, and the *LA* in the HS group decreased rapidly, although it was still slightly higher than that in the WF group, the difference was not significant (Figure 10A). In the short-term drought at the HS, we found that the *ALA* of the HS group decreased after the drainage and was significantly lower than that of the WF group. After the resuming flood irrigation, the ALA of the HS group increased rapidly and the gap with the WF group narrowed (Figure 10B). The differences in leaf phenotypic traits, mainly *LA* and *ALA*, experienced a process of increasing and then decreasing during the drought-rehydration period, i.e., the leaf phenotypes of rice were significantly different from the normal level within 1–4 days after drought beginning, and these differences were nearly eliminated after 1 day of restoration of flooding irrigation.

The above analysis indicated that the restoration irrigation after short-term water stress at the rice HS led to a compensatory adjustment of some physiological functions of rice, which was reflected in the recovery of the phenotypic traits. Likewise, the trend of the vertical phenotypic distribution traits can be consistent with this pattern.

**FIGURE 11:**
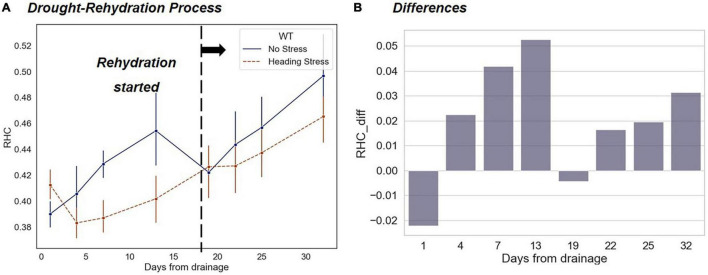
Changing trends of *RHC* indicators under short-term drought-rehydration during the heading and filling stage. **(A)** Comparison of changes between the HS and WF groups over 32 days after drought beginning. **(B)** Changes in RHC differences between the HS and WF groups.

As shown in Figure 11, during the drought-rehydration process, the differences in *RHC* between HS and WF groups experienced a fluctuant change that first increase and then decrease. On the first day after the resumption of flooding irrigation, the differences in *RHC* largely disappeared. In the following days, the *RHC* value of the HS group was still lower than that of the WF group, but the difference was smaller than that during the drought (Figure 11B). In contrast to the changing trends in leaf phenotyping traits analyzed above, the differences in the *RHC* during short-term water stress did not disappear completely after rehydration.

**FIGURE 12:**
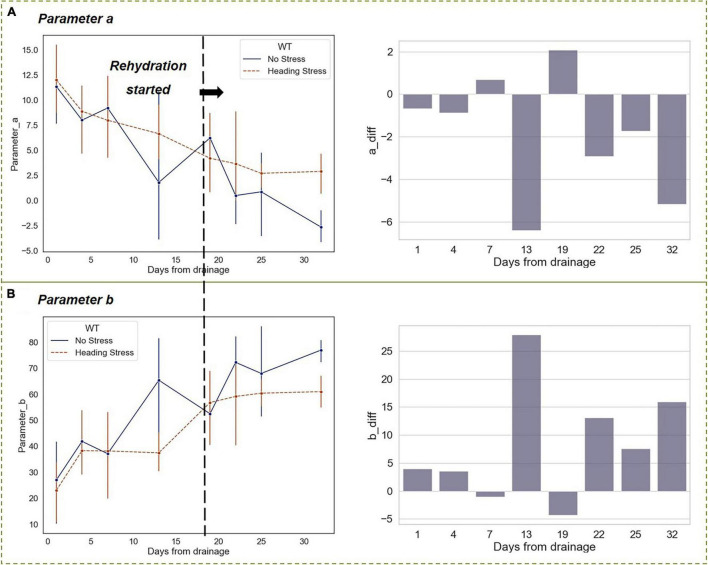
Changing trends of parameters *a* and *b* in the vertical distribution function under short-term drought-rehydration during the heading stage. **(A)** Parameter *a*. **(B)** Parameter *b*.

The differences in the vertical function parameters *a* and *b* between the HS and WF groups reached their maximum after experiencing a 2-week drought. Similar to the changes in *RHC*, the differences vanished on the first day after rehydration. However, as the growth period developed, the rice entered the stage of filling and milk-riping, and the differences in parameters *a* and *b* between the two experimental groups remained at a certain level, which was lower than that of the drought stage though (Figure 12).

**FIGURE 13:**
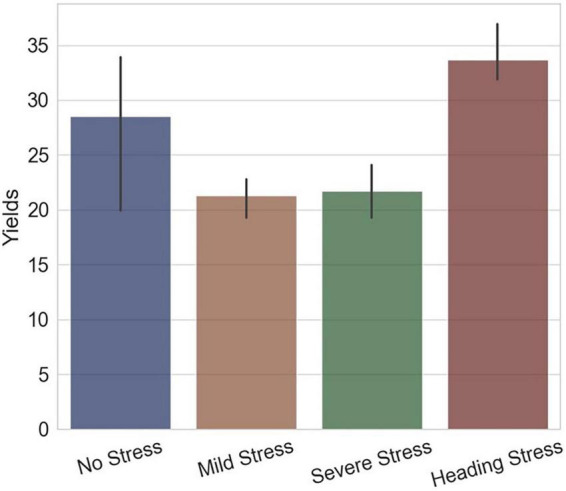
The final yields of experimental groups of different water treatments, the *x*-axis represents the groups and the *y*-axis represents the yields (g per plant).

The trends of *RHC* and parameters *a* and *b* indicated that the short-term drought-rehydration process had irreversible influences on rice at the HS, which were continuous and mechanistic. This response pattern can be used to illustrate the final yield differences, with the HS group subjected to short-term water stress having a higher yield instead. As Figure 13 represented, the yield of the WF group with flooding irrigation during the whole growth period was significantly higher than that of the WM and WS groups, which were subjected to mild or severe water stress, but the HS group had higher yields. During rice heading, the growth process of rice panicles was significantly slowed down under insufficient water supply, which could be informed by the changes in the *RHC* index. After rehydration in the HS group, the *RHC* maintained a steady and slow rising trend compared with the normal level, indicating that despite the resumption of irrigation, rice kept a lower growth focus. The drought-rehydration process gave a drawn-out period for the rice spike to grow and resulted in more nutrient accumulation. On the other hand, through the changing trends of the parameters *a* and *b*, it can be seen that the evolution of rice light interception morphology during the short-term drought-rehydration process. Under the drought condition, the functional leaves of the rice plant maintained a high degree of uprightness, and part of the leaves in the lower and middle layers was retained instead of falling, thus the radiation transfer efficiency of the plant was improved obviously.

The irrigation model of short-term drought-rehydration during the rice heading and filling stages was proved to be conducive to improving the plant morphology and structure, which was less compact and more suitable for radiation interception. Moreover, the problems of weak and bent stems and low rice panicle set rate due to prolonged flooding irrigation were mostly avoided.

### Capacity of Vertical Distribution Traits to Indicating Rice Growth Status

The vertical distribution characteristics which were worked out from digital images were proved to be effective indicators for the detection of drought response in rice plants. Many previous studies have shown that visual phenotypic indicators estimated from images can be well correlated with crop biomass as well as growth status, while the digital features involved in these studies are mainly projection areas of the plants (Honsdorf et al., 2014; Fisher et al., 2016; Duan et al., 2018). In this paper, we also analyzed the correlation between the vertical distribution traits and rice growth status. The growth and radiation interception capacity of rice were evaluated by the above-ground dry matter (*ADM*) and the extinction coefficient (*K*; Supplementary Table 3).

**FIGURE 14:**
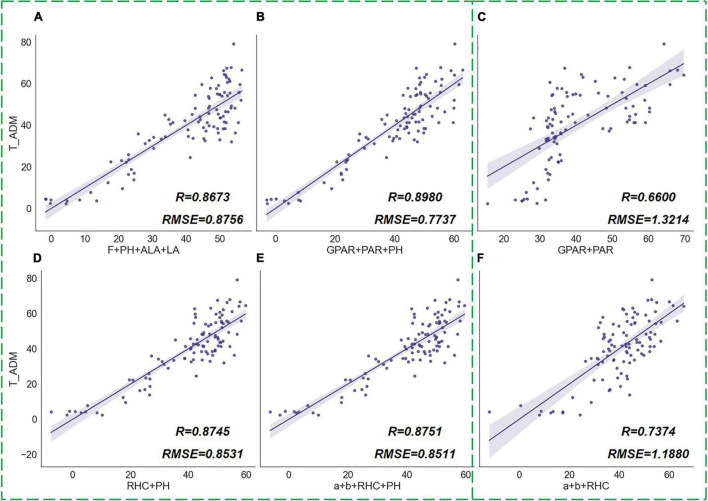
MLR results of phenotypic traits groups and above-ground dry matter of rice (*ADM*). The scatter graphs show the Pearson correlation test results between the regression values and the true measurement values. The *x*-axis represents the *ADM* values derived from the regression model, and the *y*-axis represents the measured *ADM* values. Different combinations of traits are **(A)** [*F*, *PH*, *ALA*, *LA*], **(B)** [*GPAR*, *PAR*, *PH*], **(C)** [*GPAR*, *PAR*], **(D) [***RHC*, *PH*], **(E)** [*a*, *b*, *RHC*, *PH*], **(F)** [*a*, *b*, *RHC*].

To test the capacity of different types of phenotypic traits to estimate plant growth, we grouped the calculated indicators by type and performed a multivariate correlation analysis with the measured *ADM* values separately (Figure 14). Tiller number (*F*), *PH*, *ALA*, and *LA* were combined into a group. These traditional phenotypic indicators have been commonly used in previous studies (Peng, 2000; Rahaman et al., 2015; Peng and Xie, 2020). They describe the crop morphology in more comprehensive dimensions, which indicate how many tillers, stem length, degree of leaf erectness, and leaf size of rice. Therefore, this combination of parameters had a good capacity for indicating dry matter accumulation in rice plants (Figure 14A). However, among these widely used traits, *F* and *LA* require destructive sampling measurements to obtain accurate values, which is uncomplicated, and only *ALA* can be derived from a single image. For rapid acquisition of plant morphological features from images, Duan et al. (2018) proposed two shape descriptors, *GPAR* and *PAR*, which were proved to be effective in the quantification of drought response of rice. *GPAR* is the greenness projected plant area ratio of rice that represents the proportion of the green organs in rice, and *PAR* is the perimeter area ratio which indicates the compactness of rice tillers. By calculating these two shape descriptors on our experimental images, we discovered that *GPAR* and *PAR* were not only advantageous indicators of drought response in rice but also had a high correlation with above-ground biomass. The trait set with *GPAR*, *PAR*, and *PH* as a combination had the highest accuracy of regression analysis for *ADM* (Figure 14B). The novel phenotypic traits we proposed in this paper include *RHC* and parameters *a* and *b*, which also correlated relatively well with rice biomass. Figures 14D,E showed the MLR results of the combination of *RHC* and *PH*, and the traits combination of *a*, *b*, *RHC*, and *PH* on *ADM*. The results showed that the relative centroid height and the vertical phenotypic distribution features could be a good indication of the rice growth status, and this indicating capacity was outperformed by the combination of traditional phenotypic parameters. Meanwhile, we noticed a high correlation between *PH* and *ADM*, which was because the *PH* information indicates the length of rice stems that occupy a large portion of above-ground biomass. However, accurate *PH* required manual measurements or calibrated photography measurements so that it cannot be obtained by a single digital image directly. Therefore, we analyzed the correlation between only image visual features and rice biomass when *PH* was not involved in the parameter combination. The results showed that the traits combination of *a*, *b*, and *RHC* was slightly more correlated with rice biomass than that of *GPAR* and *PAR* (Figures 14C,F).

The extinction coefficient (*K*) is also one of the most significant indicators describing the growth status of the crop. Unlike *ADM* which measures the amount of nutrient material accumulated by the plant, *K* is used to characterize the interception and absorption of light by the canopy, which is of interest for evaluating the photosynthetic efficiency. Plant morphological structure is the key factor affecting the process of radiation transformation in the canopy, so the extinction coefficient is sensitive to changes in phenotypic traits. Thus, quantifying morphological features is an important tool to evaluate the photosynthetic efficiency of plants (Saitoh et al., 2002; Duursma and Mäkelä, 2007).

**FIGURE 15:**
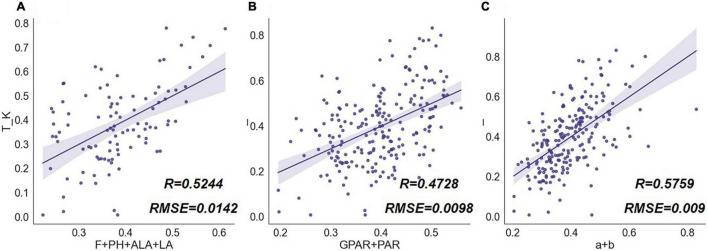
MLR results of phenotypic trait combinations and extinction coefficient (*K*). The scatter graphs show the Pearson correlation test results between the regression values and the true measurement values. The *x*-axis represents the *K* values derived from the regression model, and the *y*-axis represents *K* values measured by a radiometer. Different combinations of rice traits are **(A)** [*F*, *PH*, *ALA*, *LA*], **(B)** [*GPAR*, *PAR*], **(C)** [*a*, *b*].

To test the capacity of the phenotypic traits to describe rice structure and their correlation with the extinction coefficients, we grouped the phenotype traits by type. The results were shown in Figure 15. Among the traits groups, the conventional traits combination of *F*, *PH*, *ALA*, and *LA* was correlated with *K* to some extent, the regression accuracy of the combination of *GPAR* and *PAR* was lower, while the combination of parameters *a* and *b* had the highest regression accuracy for *K*. Among the conventional traits set, *ALA* and *LA* were indicators describing the degree of leaf inclination and leaf size, respectively. The angle and size constitute the basic structural elements in the canopy and therefore possess a certain ability to estimate the extinction coefficient. Parameters *a* and *b* had the highest regression accuracy for *K*, while the combination of *GPAR* and *PAR* had a lower correlation with *K*. The results illustrated that the vertical distribution model well described the structure of rice plants, and the hierarchical modeling contributed to the study of rice traits related to light interception and radiation transformation. *GPAR* and *PAR*, on the other hand, lack representation of the three-dimensional structure and vertical heterogeneity. It should be noted that the MLR analysis of *K* for all combinations of phenotypic traits in this research did not exhibit a very high correlation since at the single plant scale; external factors interfering with radiation measurements below the plants were complex and scabrous.

## Discussion

The vertical architecture of gramineous plants is primarily formed by the leaves and their distribution, so it is complex and difficult to accurately carve the vertical structures. Previously, methods such as manual measurement and LiDAR scanning reconstruction have been mainly applied (Fournier and Pradal, 2012; Martin-Ducup et al., 2018; Perez et al., 2019). To avoid the high cost and complicated measurement, we designed several traits to characterize the vertical morphology of rice based on a simpler image process method. By implementing experiments with several water treatments, we understood the characteristics of rice response to water stress at different growth stages with the application of these traits. Meanwhile, these traits have a good correlation with rice growth status, so while reflecting the effects of water stress, they can indicate changes in rice physiology as well as provide a more reasonable explanation for the formation of rice yield. Compared to previous drought experiments, the experiment in this study focused on a more practical irrigation model of drought-rehydration. In the experiment, the water stress was not so severe as to cause leaf rolling and wilting, but it still significantly affected the yield. Some conventional drought-sensitive indicators cannot reflect the changes in rice physiology and morphology under this situation. The novel proposed vertical traits helped to gain more knowledge about the growth compensation effect of rice under alternating drought-rehydration scenarios. For future field applications, our research results will be used to improve the phenotype module and stress description in the crop model. We aim to identify the effects of different levels of water stress on crop growth at different times through crop modeling. The image analysis model combining phenotype detection and stress identification will be used for farm management and irrigation decisions. The scientific determination of irrigation timing and quantity from rice growth mechanism is the significance of our research and the future development trend. The drought-detection method we proposed has the flexibility to realize through the photography function of a smartphone, which can be applied in other similar species and different scenarios.

## Conclusion

(1) It was found that the distribution of rice leaf angle in the vertical direction has a certain pattern, which can be expressed quantitatively by the logarithmic model and its parameters *a* and *b*. Meanwhile, this paper proposed that the relative focused location of nutrient accumulation and transformation in rice can be described by the *RHC* index.
(2) Long-term water stress produced various effects on rice growth at the nutritional and reproductive growth stages and was reflected in differences in vertical phenotypic traits. In addition, reasonable short-term water regulation had the effect of optimizing rice architecture and increasing yield accumulation, which could be reflected by changes in the vertical phenotypic traits as well.

In conclusion, water regulation affects physiological processes such as water use, photosynthesis, and nutrient transformation in rice by altering plant morphological characteristics. Our research is helpful in evaluating the real-time effects of water regulation, providing a decision basis for rationalizing irrigation systems, and adjusting water conditions, which is enlightening for studying water-use mechanisms in rice under various water conditions.

## Data Availability Statement

The original contributions presented in this study are included in the article/Supplementary Material, further inquiries can be directed to the corresponding author.

## Author Contributions

YFZ designed and performed the experiments, wrote the algorithm, analyzed the data, and wrote the manuscript. YYZ put forward the hypothesis and edited the manuscript. YW designed and performed the experiments. HQ participated in the experimental work. XJ contributed to editing and revising the manuscript. All authors contributed to the article and approved the submitted version.

## Conflict of Interest

The authors declare that the research was conducted in the absence of any commercial or financial relationships that could be construed as a potential conflict of interest.

## Publisher’s Note

All claims expressed in this article are solely those of the authors and do not necessarily represent those of their affiliated organizations, or those of the publisher, the editors and the reviewers. Any product that may be evaluated in this article, or claim that may be made by its manufacturer, is not guaranteed or endorsed by the publisher.
