# Supplementary material for: Changes in Vertical Phenotypic Traits of Rice (Oryza sativa L.) Response to Water Stress
Source: Front Plant Sci. 2022 Jul 14;13:942110. doi: 10.3389/fpls.2022.942110 (PMC9331173; doi:10.3389/fpls.2022.942110)
Supplement: Supplementary file 1 [file Data_Sheet_1.PDF]

Supplementary Data Sheet

Supplementary Table 1. The details in the experimental design of this study

Experimental group	Water stress level	Duration of flood irrigation	Duration of water stress	Sample size	Sampling date
**WF**	No stress	Day 1 to 134	\	78	Day 43, 59, 79, 92, 95, 98, 104, 110, 113, 116, 123
**WM**	Mild stress	Day 1 to 25	Day 25 to 134	42	Day 43, 59, 79, 104, 123
**WS**	Severe stress	Day 1 to 25	Day 25 to 134	42	Day 43, 59, 79, 104, 123
**HS**	Mild stress	Day 1 to 92<br>& Day 111 to 134	Day 92 to 110	60	Day 92, 95, 98, 104, 110, 113, 116, 123

Note: The number of days in Table 1 refers to the days from rice transplanting.

Supplementary Table 2. Measurement of soil physical and chemical properties

**Clay proportion %**	**Silt proportion %**	**Sand proportion %**	**Soil bulk density ($g \cdot cm^{-3}$)**	**Soil electric conductivity ($us \cdot cm^{-1}$)**	**Saturated water content %**	**Field Capacity %**	**Absorption moisture content %**
15.15	70.75	14.09	1.39	82.11	32.12	30.52	5.07

Note: The water stress levels in this study were developed based on soil mositure characteristics measured beforehand, under which we ensure the survival and growth of the rice.

Supplementary Table 3. Several rice physiological and morphological traits and their measurements in the experiment.

Traits	Nomenclature	Measurements	Units
Biomass (Aboveground dry matter)	ADM	Dry and weight	g
Extinction coefficient	*K*	Handle ratiometer	\
Plant Height	PH	Tapeline	cm
Tiller Number	*F*	Manual counting	\
Total Leaf Area	LA	Leaf area scanner	cm^2
Average Leaf Angle	ALA		°

Greenness Ratio	GPAR	Image processing and Machine vision approach	
Relative Height of Centroid	RHC		\
Logarithmic coefficient	*a*		\
	b		\
Soil Moisture Content	SMC	Dry and weight	\

Supplementary Table 4. Raw data recording sheet for all observed traits mentioned in the manuscript

**WT**	**Date**	**LA(cm^2)**	**PH(cm)**	**ALA(°)**	**GPAR**	**RHC**	***a***	***b***	**K**	**ADM(g)**	**SMC(%)**
Water treatment groups	Days from transplant	Leaf area	Plant Height	Average leaf angle	Greenness ratio	Relative height of centroid	Model parameter	Model parameter	Extinction coefficient	Above ground dry matter	Soil moisture content
WF	43		46	58.320	0.9476	0.2930	2.2100	47.1581	0.3436		
WF	43	330.3	50	57.853	0.9736	0.3488	4.9846	45.3257	0.4674	3.76	36.81
WF	43	234.3	35	53.190	0.8714	0.2916	4.6657	33.7958	0.3594	4.18	38.18
WF	43	610.3	48	52.147	0.9683	0.3292	7.1118	26.3717	0.3488	7.80	41.78
WF	43		53	56.249	0.9482	0.3206	5.4695	35.5979	0.5807		
WF	43		48.5	59.873	0.7856	0.3716	-3.3295	71.4114	0.4470		
WF	59		58	58.737	0.9592	0.3581	8.0401	25.3019	0.5673		
WF	59		53	61.084	0.9761	0.3063	19.8150	-5.8022	0.8329		
WF	59		57.5	55.566	0.9022	0.3789	16.0533	-5.3022	0.5349		
WF	59		70	62.116	0.9389	0.3946	17.0586	-3.3395	0.4421		
WF	59		64.5	56.807	0.9561	0.4112	12.8092	14.7864	0.4855		

WF	59		68.8	59.515	0.9742	0.3601	11.3915	20.1853	0.3089		
WF	79		75.5	65.034	0.9372	0.4033	23.6322	-26.3587	0.8022		
WF	79		75	64.608	0.9470	0.4026	14.4533	12.4953	0.6030		
WF	79		67.8	60.018	0.9270	0.4401	16.8230	-4.2726	0.7539		
WF	79	1221.7	76	68.172	0.9372	0.3831	7.5623	39.3701	0.7806	34.15	42.60
WF	79	2030.0	79.5	66.270	0.9099	0.3790	15.1458	8.3491	0.7775	31.13	42.86
WF	79	1381.3	71.2	67.842	0.9462	0.3931	8.3945	36.9439	0.5383	35.91	41.03
WF	92	1542.4	95.4	67.186	0.8885	0.4058	19.3801	-9.8379	0.6509	48.10	42.32
WF	92	1646.2	78.2	67.720	0.9791	0.4029	13.8889	15.1030	0.7426	28.92	42.17
WF	92	1381.8	91	68.968	0.8831	0.3704	5.7019	47.3393	0.6466	34.62	40.52
WF	92		98.3	69.840	0.9207	0.3902	6.9764	44.0472	0.6561		
WF	92		92	65.534	0.8360	0.3790	6.9025	39.4514	0.7766		
WF	92		90.3	65.932	0.9239	0.3928	15.2473	8.8090	0.6600		
WF	95	1054.8	96.7	62.725	0.8069	0.3691	12.4831	23.0405	0.5453	57.85	38.56
WF	95	980.5	98.2	67.879	0.8799	0.4298	3.2232	57.6010	0.3092	47.56	39.46

WF	95	1081.6	102.6	67.164	0.8656	0.3969	10.2141	27.5419	0.4168	46.65	45.30
WF	95		103.9	70.369	0.8738	0.4209	10.8693	30.5341	0.4383		
WF	95		87	70.253	0.9254	0.4416	3.0280	59.4003	0.2259		
WF	95		95.5	70.278	0.8726	0.3762	8.2101	38.3285	0.2958		
WF	98	957.5	99.8	68.720	0.8442	0.4333	10.3793	31.2923	0.4942	43.64	41.72
WF	98	561.4	87.5	67.496	0.7321	0.4369	3.0748	56.9834	0.6107	24.52	43.51
WF	98	903.3	105.5	69.981	0.8253	0.4450	9.2582	35.0406	0.5326	36.33	38.57
WF	98		101	71.862	0.8192	0.4185	5.9624	49.4706	0.4769		
WF	98		98	69.515	0.8446	0.4075	16.5913	4.8612	0.5296		
WF	98		95.3	71.257	0.8376	0.4318	10.0288	32.6072	0.4920		
WF	104	876.5	102.1	76.861	0.7412	0.4490	-4.5677	94.4751	0.0275	49.34	54.67
WF	104	1118.6	101.2	69.673	0.6473	0.5159	-4.6665	89.0296	0.0086	64.50	35.94
WF	104	613.3	96.5	72.511	0.7063	0.4516	1.1917	69.7535	0.1390	45.35	47.54
WF	104		107	73.376	0.8068	0.4348	-0.1338	74.7438	0.2636		

WF	104		92.8	65.389	0.7962	0.4058	23.3369	-26.8627	0.3519		
WF	104		92	73.404	0.7603	0.4694	-4.3668	92.4721	0.2605		
WF	110	684.2	100	73.741	0.6048	0.3995	8.2672	44.6061	0.4129	63.13	39.96
WF	110	864.0	102	73.105	0.7487	0.4302	3.8745	64.4857	0.2742	54.32	42.38
WF	110	1000.1	99.5	74.673	0.7335	0.4284	7.0888	48.7062	0.4303	67.53	40.87
WF	110		105	71.151	0.5826	0.4182	7.2556	45.2859	0.4075		
WF	110		100	71.744	0.5895	0.4512	3.5569	57.2896	0.2827		
WF	110		99	73.913	0.7655	0.4059	7.3359	47.7690	0.3051		
WF	113	502.8	100	72.709	0.5098	0.4377	-4.3293	87.9484	0.1397	55.55	40.53
WF	113	536.5	93	72.176	0.4498	0.4459	5.9613	49.4270	0.2477	48.27	42.67
WF	113	422.7	95	75.819	0.4738	0.4030	4.3264	58.3292	0.2867	38.44	40.18
WF	113		104	69.855	0.3628	0.4087	-0.7779	73.6128	0.2210		
WF	113		94	77.069	0.6110	0.4839	-1.5353	83.9006	0.2730		
WF	113		102	77.659	0.5196	0.4826	-0.7020	79.5051	0.2023		
WF	116	143.7	100.8	63.895	0.3000	0.4387	8.9484	30.9252	0.0092	62.19	39.92

WF	116	333.0	96.2	75.979	0.4229	0.4085	-3.7475	86.6888	0.1844	54.06	39.55
WF	116	123.6	100.7	78.654	0.2362	0.5038	1.9546	65.0337	0.0223	55.78	39.12
WF	116		102.3	73.419	0.4160	0.4790	1.3846	66.0338	0.1177		
WF	116		96.2	73.827	0.3813	0.4378	3.5324	56.8676	0.2817		
WF	116		103.7	74.355	0.3854	0.4739	-6.8853	100.3707	0.1011		
WF	123	23.8	95	77.908	0.0714	0.5361	-1.8560	76.7624	0.4756	64.13	40.99
WF	123	106.0	99	72.539	0.1641	0.5013	-2.6075	76.3428	0.4482	79.03	40.34
WF	123	144.7	103	73.047	0.1629	0.4190	-4.3289	82.1668	0.4331	52.03	37.58
WF	123		101	74.615	0.1883	0.5174	-2.9563	79.9462	0.4292		
WF	123		89	71.593	0.0716	0.4669	-5.2727	84.8073	0.3822		
WF	123		94	76.861	0.1537	0.5401	1.1243	65.1407	0.2262		
WM	43		52	49.316	0.9878	0.3065	-0.8508	60.0568	0.4581		
WM	43	316.6	42	59.467	0.9752	0.3487	-2.0792	71.0113	0.4896	3.70	35.09
WM	43	366.4	44	67.983	0.9511	0.3505	-5.0686	87.1182	0.3682	4.20	36.03

WM	43	387.5	35	55.669	0.9724	0.3773	6.3328	30.7894	0.5115	4.42	34.43
WM	43		40	60.348	0.9719	0.3550	2.0443	55.4915	0.4799		
WM	43		41	62.917	0.9759	0.3309	9.1091	33.5346	0.2952		
WM	59		63	59.561	0.9390	0.3068	3.8027	49.7201	0.4574		
WM	59		65	65.633	0.9393	0.3882	5.1730	48.8303	0.4422		
WM	59		63	67.706	0.9389	0.3218	11.3842	26.8702	0.4361		
WM	59		66	68.488	0.9511	0.3297	10.5790	27.2991	0.4959		
WM	59		67.1	70.228	0.9506	0.2611	6.2828	42.7498	0.4000		
WM	59		61.8	60.117	0.9521	0.3008	6.3074	42.0055	0.3558		
WM	79	494.7	59.5	58.730	0.9330	0.3750	7.3801	33.2621	0.4986	12.50	31.50
WM	79		71	71.820	0.9441	0.4590	2.9212	59.9326	0.4810		
WM	79		67.5	69.083	0.9376	0.3989	5.0145	51.5913	0.5588		
WM	79	281.3	65	67.414	0.9140	0.4128	5.0002	48.1105	0.5844	16.92	41.52
WM	79	960.4	68	68.744	0.9367	0.4102	9.8929	31.5487	0.5205	18.75	31.92
WM	79	782.6	62.7	68.436	0.8614	0.3613	8.9783	35.4536	0.4733	9.60	38.05

WM	104	876.5	102.1	76.861	0.8265	0.4318	10.3303	28.4153	0.3751	32.08	32.84
WM	104	1118.6	101.2	69.673	0.8421	0.4184	1.4934	65.7205	0.2782	41.70	32.23
WM	104	613.3	96.5	72.511	0.7712	0.4308	-0.1000	72.7777	0.2934	36.35	31.62
WM	104		107	73.376	0.8033	0.3955	3.7531	56.3033	0.1836		
WM	104		92.8	65.389	0.8233	0.4230	-4.2554	87.9934	0.4707		
WM	104		92	73.404	0.8070	0.4444	-4.3052	86.5514	0.4290		
WM	123	458.0	87	77.425	0.3320	0.4640	-3.6065	84.7429	0.3587	41.60	35.14
WM	123	487.2	81	69.086	0.3699	0.4443	5.7554	44.0544	0.4671	41.39	34.06
WM	123	487.9	90	72.807	0.3459	0.4196	6.1898	47.0484	0.2410	42.67	32.62
WM	123		91.5	77.042	0.2512	0.4313	0.9683	67.0663	0.3297		
WM	123		93	69.876	0.4004	0.4041	-3.9922	85.0157	0.1979		
WM	123		91	72.883	0.2933	0.4500	-1.0879	73.8936	0.2352		
WS	43		37	56.2914	0.9520	0.2908	8.0405	37.9761	0.2960		
WS	43	245.95	41	65.9706	0.9742	0.3053	-0.9928	71.3427	0.2710	2.40	30.23

WS	43	274.33	41	45.3676	0.9272	0.4322	-13.5065	112.0316	0.2878	2.50	30.79
WS	43	476.27	37	50.8208	0.9381	0.3426	0.3731	58.6999	0.4479	4.54	30.60
WS	43		38	45.5187	0.9645	0.3340	-3.4587	65.7081	0.4197		
WS	43		36	49.63	0.9228	0.3255	10.9492	17.3761	0.3862		
WS	59		51.2	56.8199	0.9598	0.3572	13.9701	13.8565	0.5000		
WS	59		56.5	61.3974	0.9623	0.3364	8.7775	29.8920	0.4492		
WS	59		58	59.8604	0.9384	0.3081	9.3124	32.0323	0.5611		
WS	59		60	59.1751	0.9601	0.3490	8.9510	33.0870	0.2981		
WS	59		68.5	60.5441	0.9682	0.3241	9.3901	28.0692	0.5697		
WS	59		62.7	69.1671	0.9574	0.3359	7.3810	41.0902	0.5063		
WS	79		63.2	71.3444	0.9523	0.4253	7.8045	41.4817	0.6428		
WS	79		59	67.8275	0.9193	0.4215	9.3323	33.4349	0.6005		
WS	79		56.5	71.5643	0.9386	0.4281	6.3990	47.9955	0.4000		
WS	79	759.373	64.5	67.3666	0.9282	0.3987	0.6054	69.0919	0.3710	16.38	26.96
WS	79	1108.13	65	68.1692	0.9157	0.3701	14.0060	13.5205	0.3958	22.26	30.02

WS	79	1055.374	60.5	68.7035	0.9266	0.3895	8.0631	37.3967	0.4770	22.42	28.04
WS	104	1321.686	84.4	67.8919	0.8489	0.4484	1.3790	62.5331	0.3820	42.80	30.43
WS	104	1088.025	73.8	68.0366	0.8757	0.4129	6.3218	47.0740	0.2719	32.77	32.59
WS	104	951.698	79.1	67.5025	0.8698	0.4163	1.3860	63.7322	0.3069	33.51	28.76
WS	104		81.8	68.1848	0.8724	0.4529	-2.6705	78.6010	0.1780		
WS	104		81.9	68.8203	0.8823	0.4257	6.3640	46.3217	0.3291		
WS	104		88.2	67.1085	0.8653	0.3920	4.4120	51.1675	0.3173		
WS	123	276.315	85	74.7739	0.2703	0.4577	0.5833	66.4825	0.6208	45.26	33.52
WS	123	326.615	93.5	71.1072	0.3343	0.4377	3.2642	53.4327	0.2810	52.94	33.56
WS	123	385.774	93	72.7164	0.3077	0.4244	4.6088	51.9858	0.2910	49.21	32.89
WS	123		100	72.7506	0.3675	0.4046	1.6801	62.8833	0.3816		
WS	123		86	71.4552	0.4424	0.4223	1.9535	60.1466	0.4812		
WS	123		86	71.5662	0.3724	0.4101	1.0076	63.1829	0.4494		
HS	92	1771.886	93	65.2492	0.8883	0.4059	12.6170	18.1092	0.7107	42.29	23.49

HS	92	1946.808	87.5	66.7051	0.8848	0.3963	11.0314	21.8436	0.7080	34.46	37.80
HS	92	1935.216	86.4	67.7644	0.9040	0.4264	19.1952	-7.7923	0.6624	40.53	38.80
HS	92		84	72.6478	0.9026	0.4100	11.8271	26.0462	0.6250		
HS	92		94	66.2242	0.8898	0.3986	11.6285	21.5984	0.7864		
HS	92		93.2	65.443	0.8368	0.4373	5.7006	41.3933	0.6921		
HS	95	817.748	91.2	70.4463	0.8805	0.3928	-1.2958	76.0489	0.1742	32.62	31.27
HS	95	1221.15	91.1	66.287	0.9036	0.4013	10.8870	27.0312	0.3862	33.84	31.33
HS	95	1319.722	95.6	72.172	0.8933	0.3584	12.2744	26.1550	0.3631	45.69	31.14
HS	95		90.3	65.8053	0.8711	0.3944	11.2867	25.0297	0.5390		
HS	95		96.1	66.1875	0.9213	0.3693	9.4570	33.3331	0.5972		
HS	95		92.2	69.651	0.9111	0.3831	10.6946	26.9400	0.6032		
HS	98	1173.521	102	66.1875	0.8132	0.3830	2.6578	55.8063	0.4128	32.97	31.58
HS	98	1087.744	93	72.2758	0.8212	0.4189	8.9489	38.4181	0.4422	33.56	32.89
HS	98	920.499	100.5	71.27826667	0.8795	0.3794	4.8509	53.2480	0.4548	38.37	34.62
HS	98		96.2	68.6236	0.9456	0.3687	11.1990	28.9070	0.4752		

HS	98		101	72.42	0.8943	0.3803	3.3347	61.1753	0.3324		
HS	98		91	60.61286667	0.8570	0.3917	16.9681	-3.7000	0.4013		
HS	104		100.5	67.1615	0.8100	0.3575	12.1044	23.1719	0.3987	54.83	
HS	104		107.1	67.8313	0.8395	0.3935	4.2905	53.9998	0.4160	54.78	
HS	104		100.5	69.5597	0.8395	0.4009	11.0416	29.3697	0.4970	44.2	
HS	104	1365.357	101.9	72.2851	0.8117	0.4259	3.4399	60.2856	0.3245		32.48
HS	104	1308.777	102.1	61.1168	0.8525	0.4026	4.2325	50.1661	0.3343		32.40
HS	104	1111.781	94	63.1255	0.8470	0.4309	4.8103	44.9311	0.4930		32.90
HS	110	915.147	96	69.2377	0.7391	0.4294	6.0039	47.5661	0.3012	53.81	35.26
HS	110	768.198	94	71.8989	0.8203	0.3703	2.8541	65.0871	0.3676	39.83	36.58
HS	110	848.891	100	66.6406	0.7026	0.4327	0.3712	71.1105	0.2399	57.68	37.11
HS	110		106	71.9405	0.8203	0.4333	0.4180	72.4784	0.2173		
HS	110		99	67.1133	0.6740	0.4437	15.0904	15.5856	0.1548		
HS	110		93	63.6411	0.7991	0.4503	0.5032	61.4601	0.2205		

HS	113	762.47	90	68.1519	0.5983	0.4111	15.5072	12.5137	0.2388	54.52	36.15
HS	113	836.785	103	75.5565	0.5801	0.4545	2.0661	67.1101	0.3595	61.09	36.30
HS	113	668.861	94	73.4332	0.6592	0.4174	-2.1196	78.5076	0.2247	41.36	37.28
HS	113		100	71.3971	0.6708	0.4632	1.4905	66.8683	0.2751		
HS	113		102.5	69.758	0.6502	0.3908	2.1463	63.0985	0.1998		
HS	113		93	71.805	0.7044	0.4263	2.8804	59.9601	0.4264		
HS	116	452.084	91.2	78.1107	0.4316	0.4051	3.5727	59.7752	0.2400	62.74	37.27
HS	116	368.51	91.7	74.3517	0.4612	0.4470	2.1627	63.7050	0.1721	37.23	37.50
HS	116	248.625	99.3	72.4205	0.2456	0.4737	3.0841	57.7951	0.0820	67.82	36.85
HS	116		95.6	69.3937	0.2408	0.4330	-0.4115	70.7850	0.2981		
HS	116		91.6	71.4268	0.3764	0.4114	3.7343	56.9206	0.3568		
HS	116		99.4	65.7229	0.5091	0.4538	4.0932	48.5707	0.2833		
HS	123	243.223	101.5	72.7776	0.2411	0.4833	3.2498	56.2687	0.5509	48.28	37.78
HS	123	61.907	100	78.8052	0.1238	0.4189	5.0588	52.0643	0.3119	59.6	38.36
HS	123	154.381	102	71.2047	0.2636	0.4536	4.6037	56.2418	0.2005	41.34	39.47

HS	123	94.5	76.5537	0.0946	0.4809	0.2860	71.4129	0.5149
HS	123	106	77.738	0.1944	0.4750	5.0034	52.3671	0.3994
HS	123	98	67.9327	0.1252	0.4804	-0.8288	71.6295	0.2851

Supplementary Table 5. ANOVA with *Post hoc* comparison using Turkey HSD method to test the responding sensitivity of the rice traits under long-term water stress.

**Traits**	**DFT**	**Type of data**	**Dof**	**Sum of sq**	**Sum of mean_sq**	**F**	**α (>F)**	***Post hoc* rejection**
**LA(cm^2)**	43	WT	2	5354.70	2677.35	0.1453	0.8677	\
		Residual	6	110521.6	18420.27			
	79	WT	2	1438571	719285.6	7.0962	0.0207*	\
		Residual	7	709536.9	101362.4			
	104	WT	2	126002.3	63001.17	1.1614	0.374	
		Residual	6	325477.5	54246.25			
	123	WT	2	227735.2	113867.6	48.0840	0.0002***	\
		Residual	6	14208.58	2368.097			

**ALA(°)**	43	WT	2	148.69	74.35	2.0100	0.1685	\
		Residual	15	554.82	36.99			
	59	WT	2	123.51	61.76	4.2078	0.0354*	WF - WM
		Residual	15	220.15	14.68			
	79	WT	2	44.27	22.14	2.0594	0.1621	\
		Residual	15	161.24	10.75			
	104	WT	2	62.25	31.12	3.0069	0.07978	\
		Residual	15	155.26	10.35			
	123	WT	2	12.59	6.29	0.9299	0.4162	\
		Residual	15	101.53	6.77			
**PH(cm)**	43	WT	2	212.69	106.35	4.2705	0.0340*	WF - WS
		Residual	15	373.54	24.90			
	59	WT	2	70.10	35.05	1.2336	0.3192	\
		Residual	15	426.21	28.41			
	79	WT	2	504.35	252.18	16.85	0.00015***	WF - WM

		Residual	15	224.50	14.97			WF - WS
	104	WT	2	1165.08	582.45	18.96	7.83E-05****	WF - WS
		Residual	15	460.95	30.73			WM - WS
	123	WT	2	209.03	104.51	3.8781	0.0439*	WF - WM
		Residual	15	404.25	26.95			
**RHC**	43	WT	2	0.0011	0.0006	0.4182	0.6657	\
		Residual	15	0.0201	0.0013			
	59	WT	2	0.0078	0.0039	3.4537	0.0584	\
		Residual	15	0.0169	0.0011			
	79	WT	2	8.5E-05	4.3E-05	0.0585	0.9434	\
		Residual	15	0.0109	0.0007			
	104	WT	2	0.0036	0.0018	2.5147	0.1143	\
		Residual	15	0.0108	0.0007			
	123	WT	2	0.0167	0.0084	8.4635	0.0035**	WF - WM

		Residual	15	0.0149	0.0010			WF - WS
a	43	WT	2	32.71	16.36	0.4148	0.6678	\
		Residual	15	591.50	39.43			
	59	WT	2	149.27	74.64	6.8964	0.0075**	WF - WM
		Residual	15	162.34	10.82			
	79	WT	2	212.55	106.28	5.2310	0.0189*	WF - WM
		Residual	15	304.74	20.32			
	104	WT	2	8.98	4.49	0.0840	0.9199	\
		Residual	15	801.68	53.45			
	123	WT	2	73.58	36.79	4.0726	0.0387*	WF - WS
		Residual	15	135.49	9.03			
**b**	43	WT	2	970.88	485.44	0.8307	0.4548	\
		Residual	15	8765.89	584.39			
	59	WT	2	3207.68	1603.84	12.6208	0.0006***	WM - WF
		Residual	15	1906.18	127.08			WS - WF

79	WT	2	3821.98	1910.99	5.2624	0.0186*	WM - WF
	Residual	15	5447.11	363.14			WS - WF
104	WT	2	239.18	119.59	0.1282	0.8806	\
	Residual	15	13993.2	932.88			
123	WT	2	965.73	482.86	3.5954	0.0530	WS - WF
	Residual	15	2014.50	134.30			

Note: (1) ***DFT*** *meaned days from transplanting. (2) In the* ***Table 4***, ***DoF*** *refered to the degrees of freedom.* ***Sum of sq*** *refered to the sum of deviations squared, and* ***Sum of mean_sq*** *indicated the sum of mean squares of deviations. α(>F) showed the significance corresponding to the F value, and the asterisk in the superscript described the level of significance. (3)* ***Post hoc rejection*** *indicated experimental group couples that were significantly different using the Turkey HSD post hoc analysis. The expression form like* ***WF - WM*** *indicated that there was a significant difference between the* ***WF*** *and* ***WS*** *groups for the trait at the specific period, and that the former value was greater.*

Supplementary Table 6. ANOVA with *Post hoc* comparison using Turkey HSD method to test the responding sensitivity of the rice traits under short-term drought-rehydration.

Traits	DFD	Type of data	Dof	Sum of sq	Sum of mean_sq	F	α (>F)	*Post hoc* rejection
**LA(cm^2)**	1	WT	1	195633.9	195633.9	14.3258	0.0194*	\
		Residual	4	54624.2	13656			
	4	WT	1	9736	9736	0.265	0.6338	\
		Residual	4	146954.7	36738.7			
	7	WT	1	96179.8	96179.8	3.0692	0.1547	\
		Residual	4	125347.9	31337			
	13	WT	1	231087.1	231087.1	5.6652	0.0760	\
		Residual	4	163163	40790.7			
	19	WT	1	42.94	42.94	0.0028	0.9602	\
		Residual	4	61037.8	15259.4			
	22	WT	1	108322.6	108322.6	20.6318	0.0105*	\
		Residual	4	21001.1	5250.3			

	25	WT	1	36641.3	36641.3	3.0777	0.1542	\
		Residual	4	47621.6	11905.4			
	32	WT	1	5703.6	5703.6	0.9481	0.3853	\
		Residual	4	24063.8	6015.9			
**ALA(°)**	1	WT	1	0.1094	0.1094	0.021	0.8876	\
		Residual	10	52.07	5.207			
	4	WT	1	0.2947	0.2947	0.0366	0.852	\
		Residual	10	80.41	8.041			
	7	WT	1	4.605	4.605	0.3907	0.5459	\
		Residual	10	117.8	11.78			
	13	WT	1	75.67	75.67	4.6869	0.0556	\
		Residual	10	161.5	16.15			
	19	WT	1	64.66	64.66	10.42	0.0091**	WF - HS
		Residual	10	62.05	6.205			

	22	WT	1	19.22	19.22	2.3333	0.1576	\
		Residual	10	82.35	8.235			
	25	WT	1	6.3108	6.3108	0.2933	0.6	\
		Residual	10	215.2	21.52			
	32	WT	1	0.2001	0.2001	0.0165	0.9003	\
		Residual	10	121.1	12.11			
**PH(cm)**	1	WT	1	4.201	4.201	0.1282	0.7278	\
		Residual	10	327.7	32.77			
	4	WT	1	62.56	62.56	2.9383	0.1173	\
		Residual	10	212.9	21.29			
	7	WT	1	0.9633	0.9633	0.0331	0.8593	\
		Residual	10	291.3	29.13			
	13	WT	1	17.52	17.52	0.6741	0.4308	\
		Residual	10	259.9	25.99			
	19	WT	1	25.52	25.52	1.8333	0.2055	\

		Residual	10	139.2	13.92			
	22	WT	1	2.521	2.521	0.0988	0.7598	\
		Residual	10	255.2	25.52			
	25	WT	1	80.6	80.6	6.508	0.0288*	WF – HS
		Residual	10	123.8	12.38			
	32	WT	1	36.75	36.75	1.7611	0.214	\
		Residual	10	208.7	20.87			
**RHC**	1	WT	1	0.0015	0.0015	6.6128	0.0278*	HS - WF
		Residual	10	0.0022	0.0002			
	4	WT	1	0.0002	0.0002	2.6531	0.1344	\
		Residual	10	0.0057	0.0006			
	7	WT	1	0.0053	0.0053	21.782	0.0009***	WF - HS
		Residual	10	0.0024	0.0002			
	13	WT	1	0.0083	0.0083	8.0767	0.0175*	WF - HS

		Residual	10	0.0102	0.001			
	19	WT	1	5.76E-05	5.76E-05	0.0985	0.7601	\
		Residual	10	0.0059	0.0006			
	22	WT	1	0.0008	0.0008	0.8272	0.3845	\
		Residual	10	0.0098	0.001			
	25	WT	1	0.0012	0.0012	1.2238	0.2945	\
		Residual	10	0.0094	0.0009			
	32	WT	1	0.003	0.003	2.121	0.176	\
		Residual	10	0.014	0.0014			
a	1	WT	1	1.269	1.269	0.0508	0.8262	\
		Residual	10	249.9	24.99			
	4	WT	1	2.320	2.320	0.1108	0.7461	\
		Residual	10	209.3	20.93			
	7	WT	1	4.484	4.484	0.1749	0.6817	\
		Residual	10	256.5	25.65			

	13	WT	1	70.69	70.69	1.067	0.3259	\
		Residual	10	662.5	66.25			
	19	WT	1	12.28	12.28	0.6596	0.4356	\
		Residual	10	186.2	18.62			
	22	WT	1	30.17	30.17	1.1653	0.3057	\
		Residual	10	258.9	25.89			
	25	WT	1	10.17	10.17	0.6014	0.456	\
		Residual	10	169.1	16.91			
	32	WT	1	92.24	92.24	16.04	0.0025^{**}	WF - HS
		Residual	10	57.52	5.752			
b	1	WT	1	46.86	46.86	0.1197	0.7366	\
		Residual	10	3916.1	391.6			
	4	WT	1	40.0	40.0	0.1246	0.7314	\
		Residual	10	3209.8	321.0			

7	WT	1	46.41	46.41	0.1026	0.7553	\
	Residual	10	4523.2	452.3			
13	WT	1	1445.1	1445.1	1.224	0.2945	\
	Residual	10	11806.3	1180.6			
19	WT	1	52.69	52.69	0.2007	0.6637	\
	Residual	10	2625.4	262.5			
22	WT	1	597.3	597.3	1.559	0.2403	\
	Residual	10	3832.3	383.2			
25	WT	1	195.0	195.0	0.6128	0.4519	\
	Residual	10	3181.6	318.2			
32	WT	1	921.9	921.9	14.17	0.0037^{**}	HS - WF
	Residual	10	650.6	65.06			

*Note: **DFD** meaned the days from the start of drought process.*

Supplementary Table 7. Correlation analysis to test the capacity of the rice traits combinations to indicate the rice growth.

**Traits combination**	**Correlation growth index**	**Correlation coefficient R**	**Mean absolute error MAE**	**Mean squared error MSE**	**Root mean squared error RMSE**
F, PH, LA, ALA	ADM(g)	0.8673	7.1975	76.6717	0.8756
PH, GPAR, PAR		0.8980	6.2371	59.8600	0.7737
PH, RHC		0.8745	6.8246	72.7715	0.8531
RHC, *a*, *b*		0.7374	9.8498	141.1399	1.1880
PH, RHC, *a*, *b*		0.8751	6.8035	72.4374	0.8511
GPAR, PAR		0.6600	10.8362	174.6080	1.3214
F, PH, LA, ALA	K	0.5244	0.1161	0.0199	0.0142
GPAR, PAR		0.4728	0.1172	0.0205	0.0098
a, *b*		0.5759	0.1063	0.0177	0.009

Note: F refered to the amount of rice tillering.
