# Supplementary material for: Changes in Vertical Phenotypic Traits of Rice (Oryza sativa L.) Response to Water Stress
Source: Front Plant Sci. 2022 Jul 14;13:942110. doi: 10.3389/fpls.2022.942110 (PMC9331173; doi:10.3389/fpls.2022.942110)

Potted Plants image series

The PDF listed the series of sampling images which were scaled to a approximate size. And they were grouped according to the sampling date and water treatment.

Sampling Date：2021-7-14　Growth Period: Tillering

Experimental Group：WF

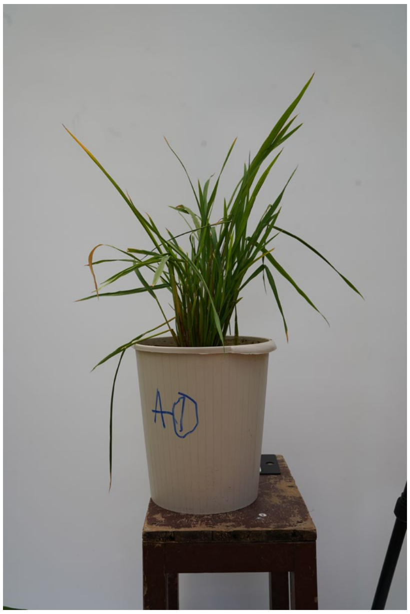
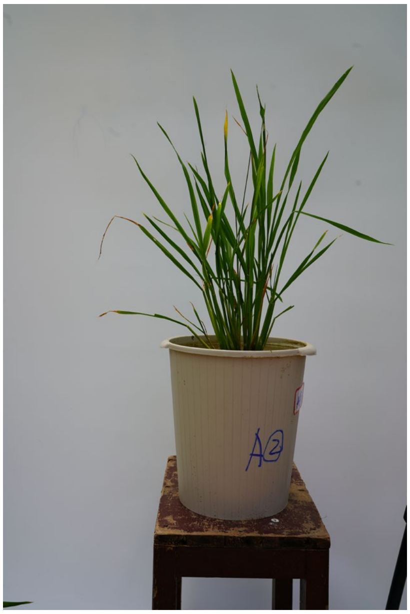
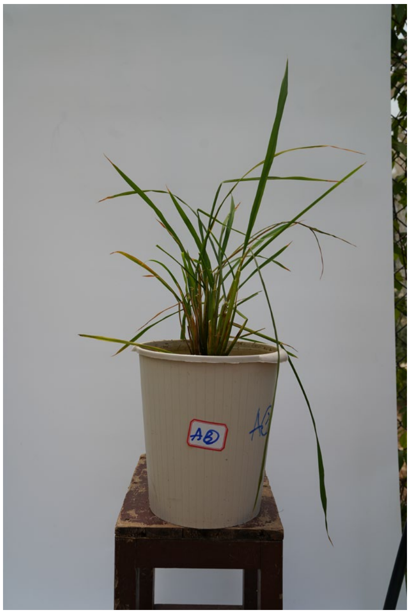
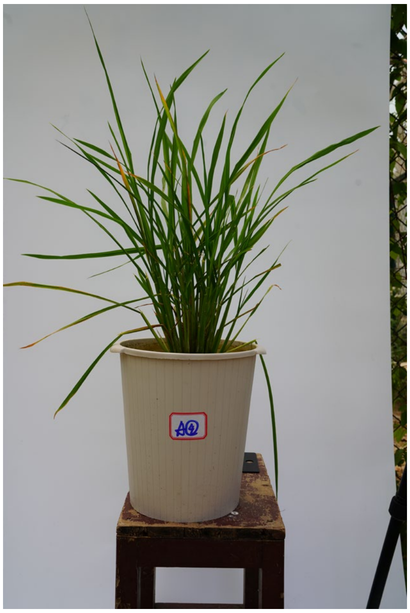
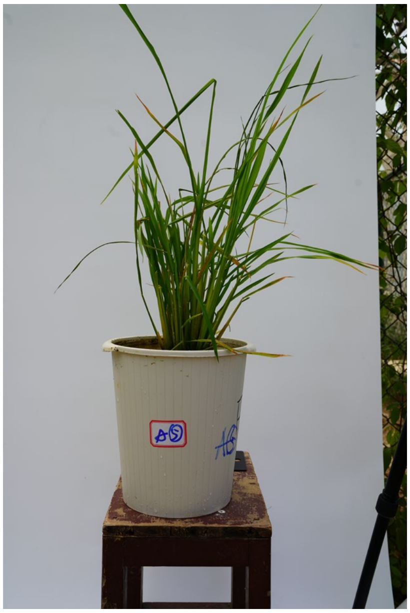
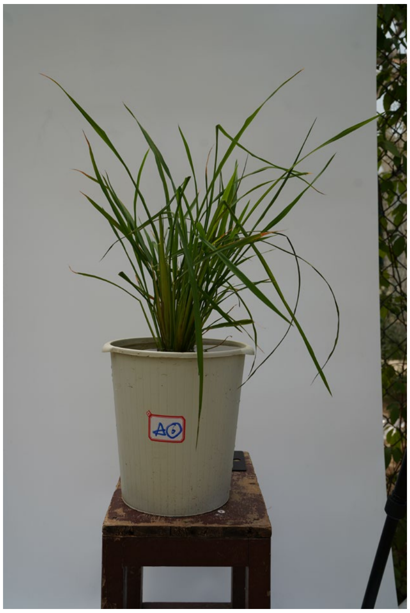

Sampling Date： 2021-7-14 Growth Stage: Tillering

Experimental Group： WM

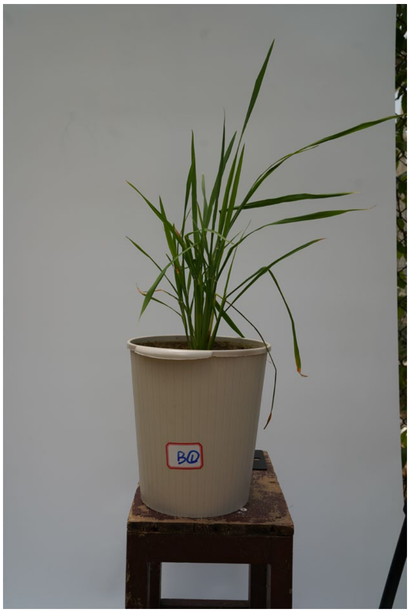
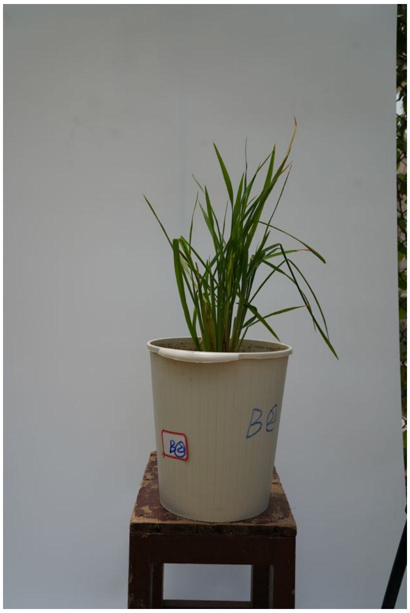
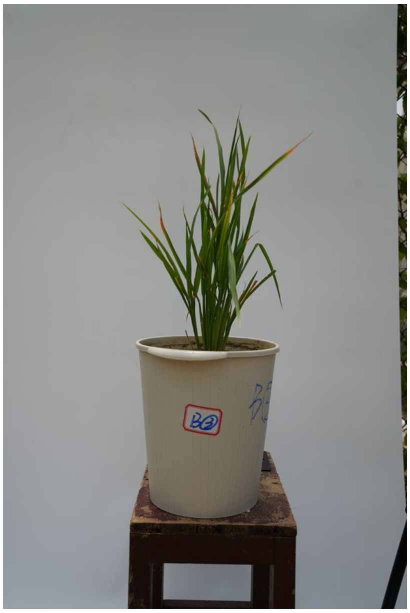
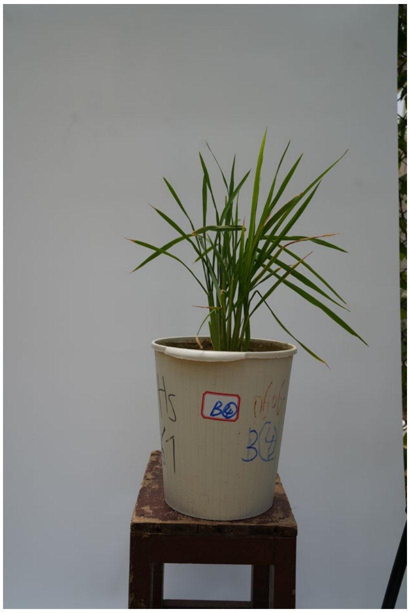
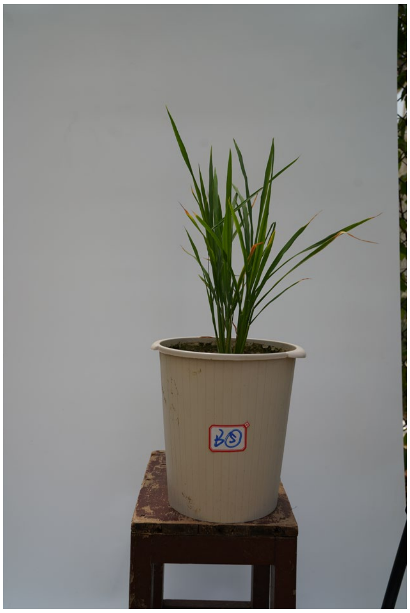
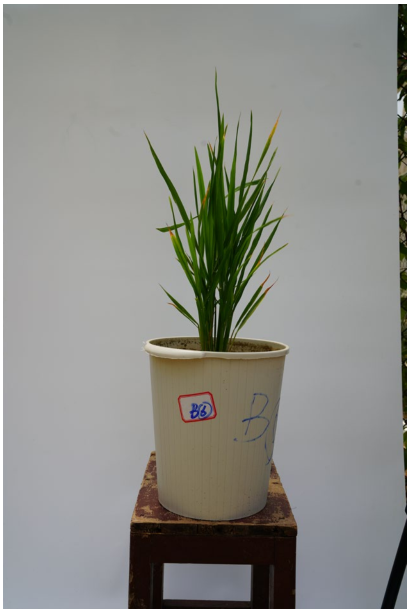

Sampling Date：2021-7-14　Growth Stage: Tillering

Experimental Group：WM

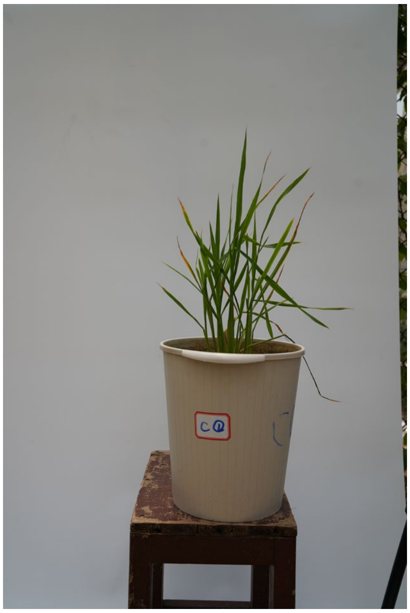
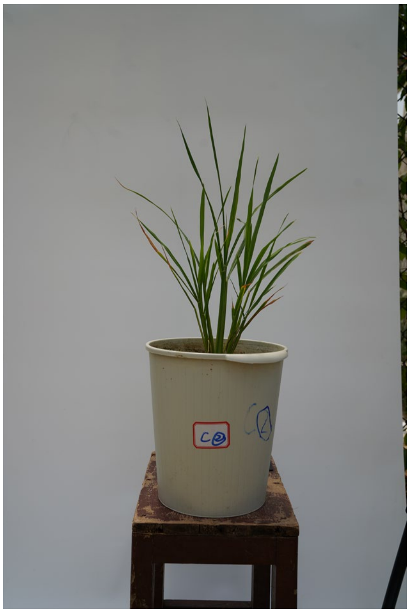
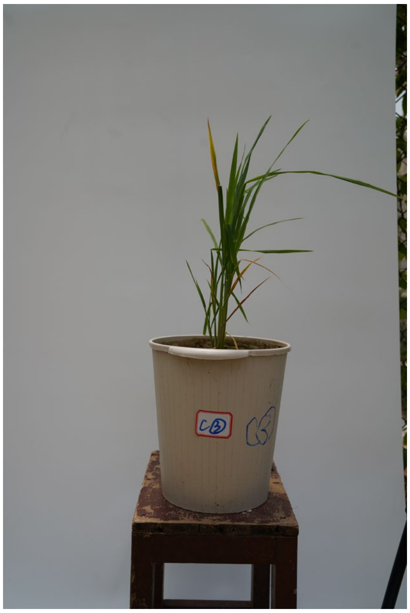
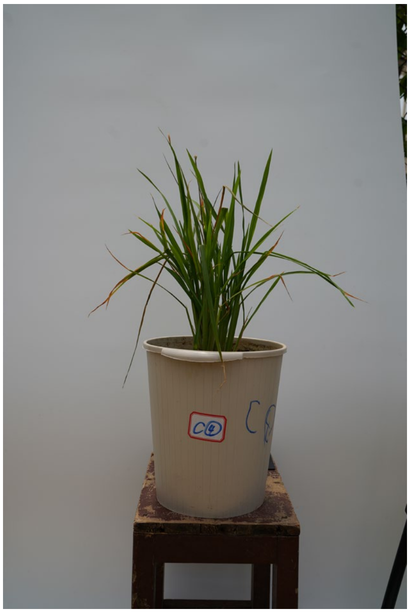
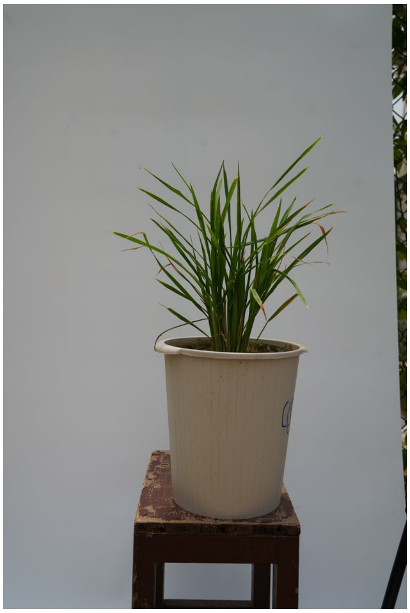
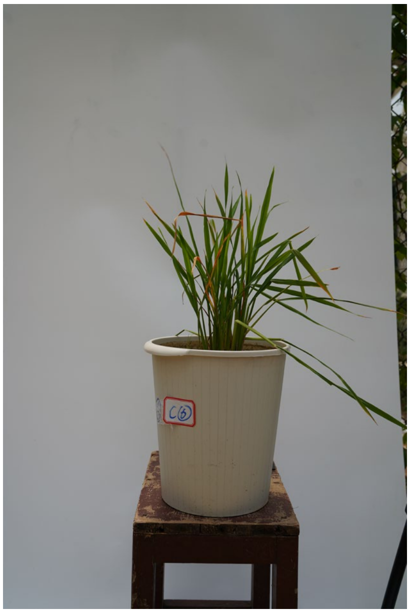

Sampling Date：2021-7-30 Growth Stage: Jointing

Experimental Group：WM

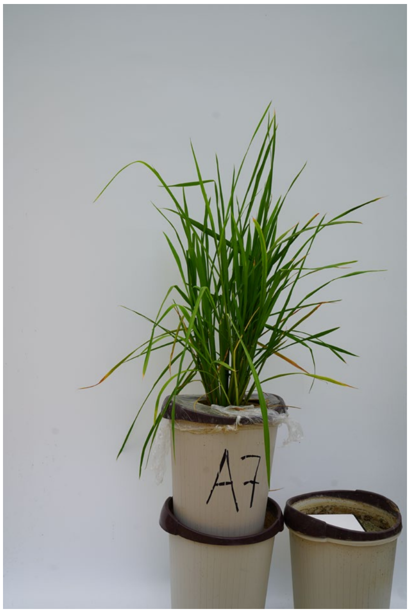

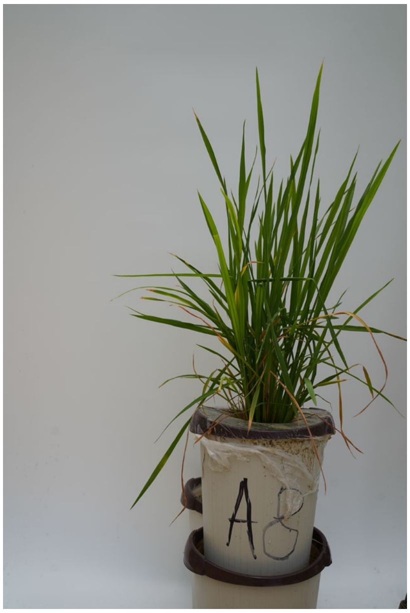

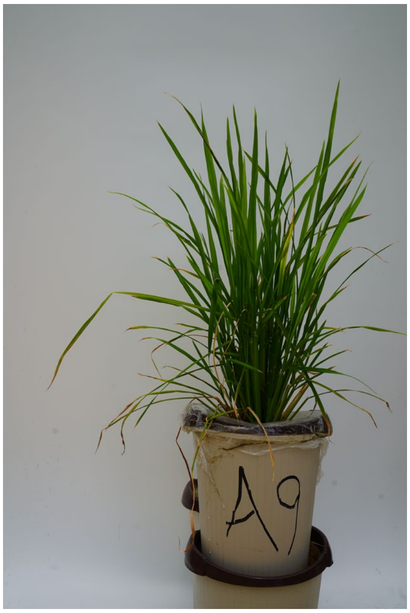

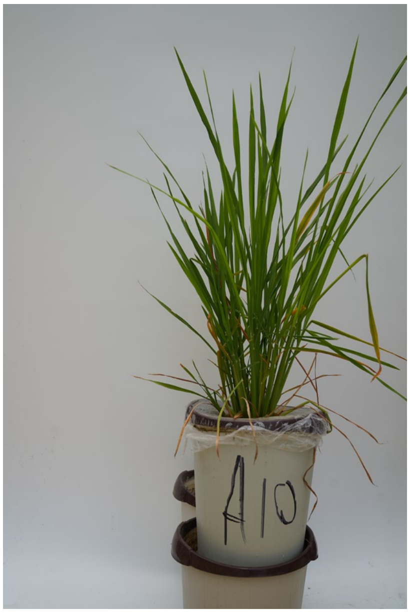

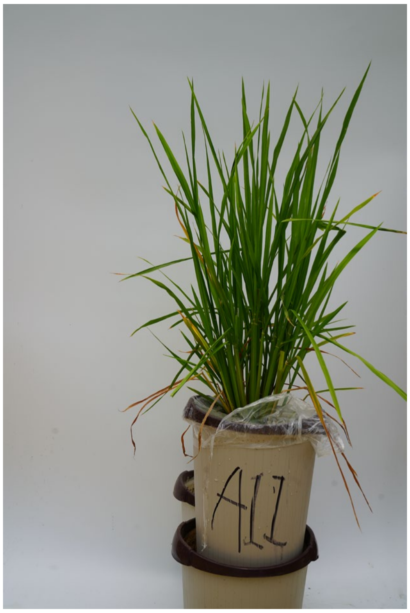

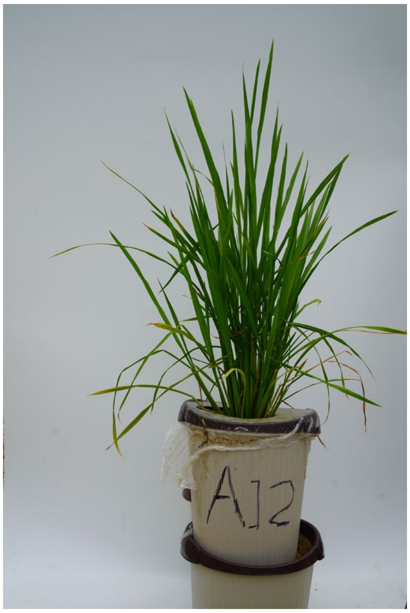

Sampling Date：2021-7-30 Growth Stage: Jointing

Experimental Group：WM

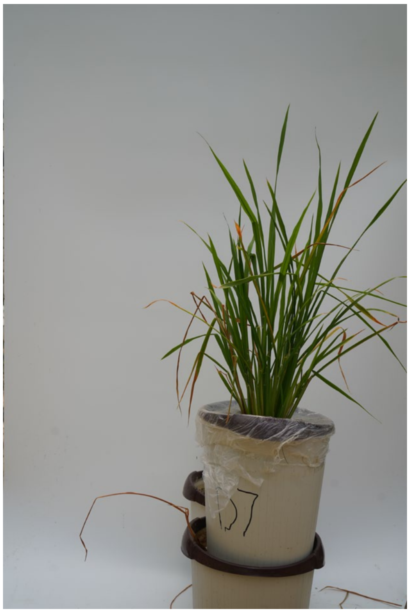

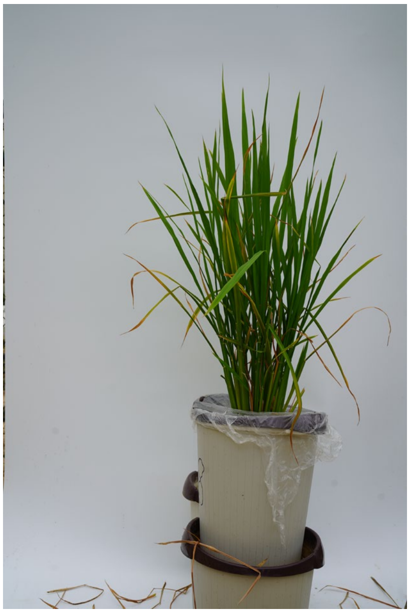

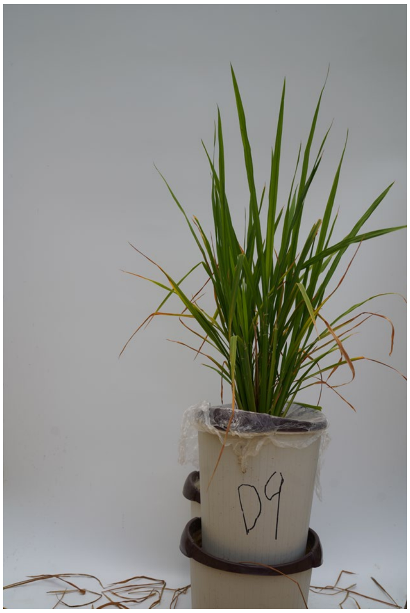

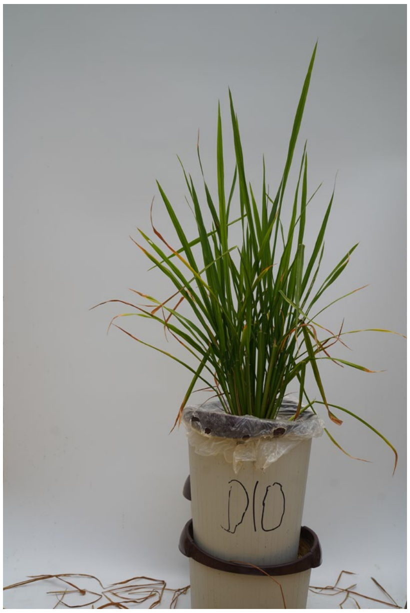

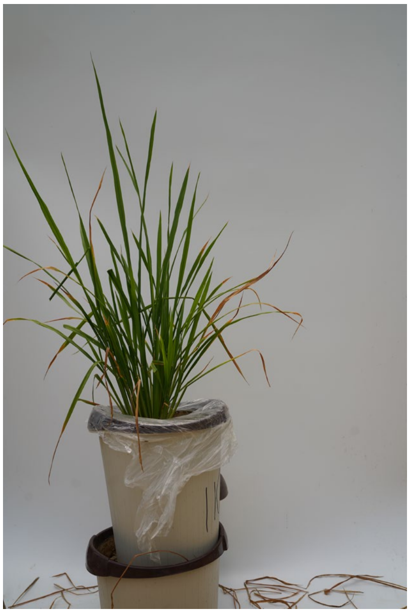

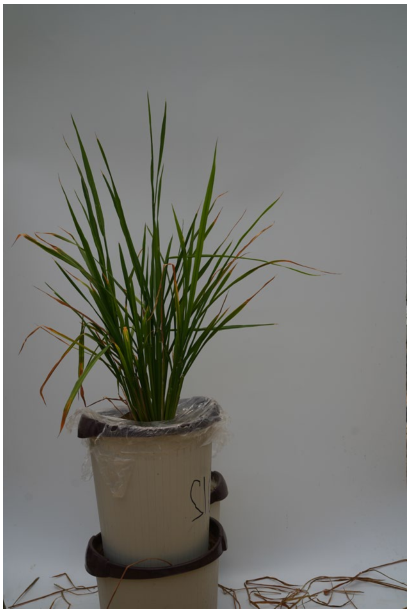

Sampling Date：2021-7-30 Growth Stage: Jointing

Experimental Group：WS

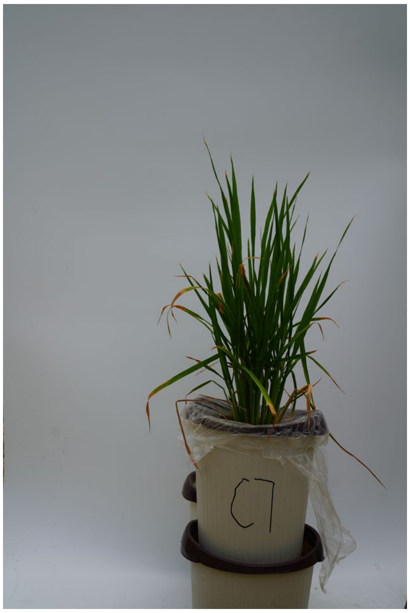

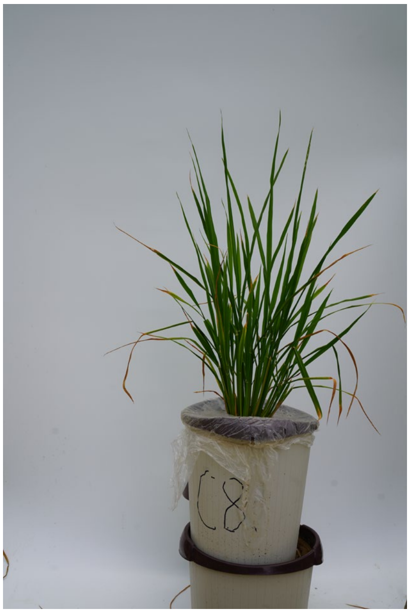

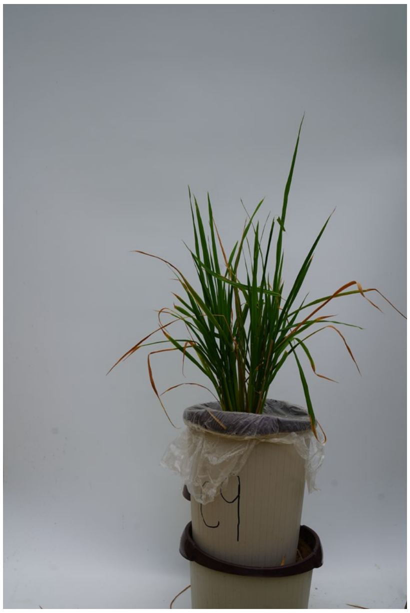

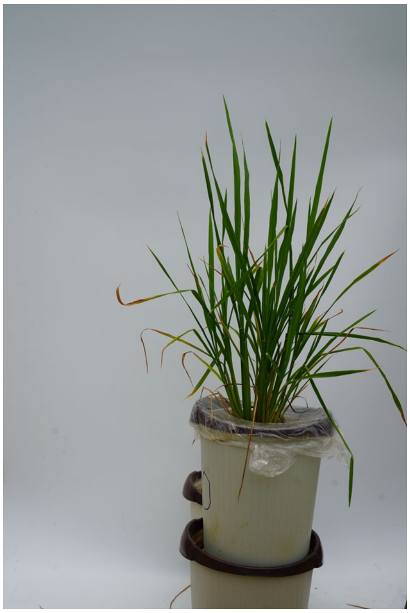

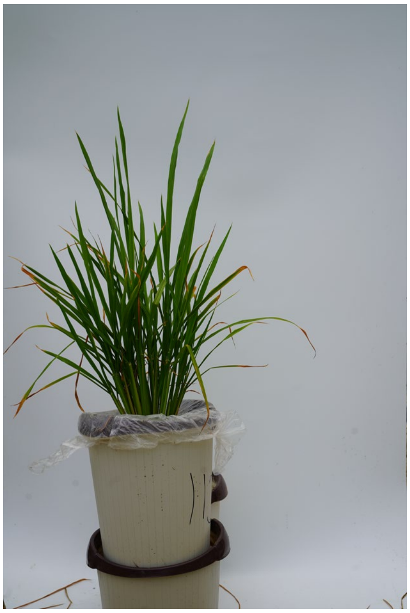

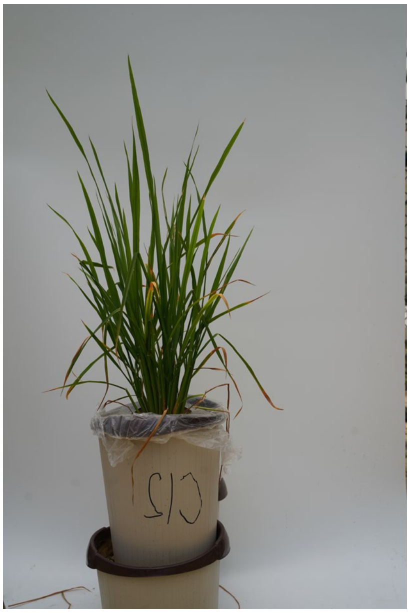

Sampling Date：2021-8-19　Growth Stage: Jointing

Experimental Group：WF

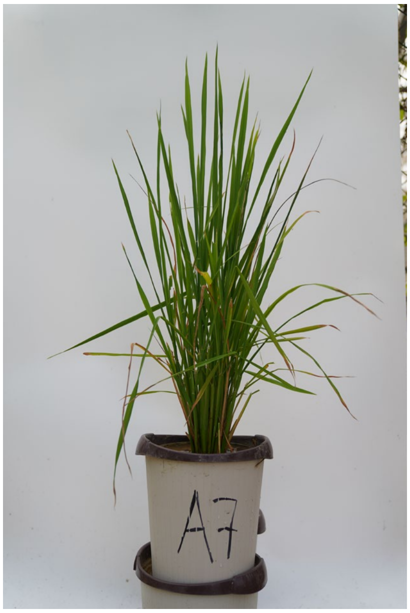

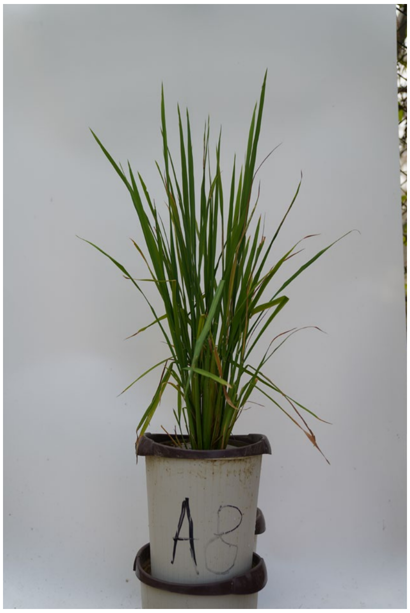

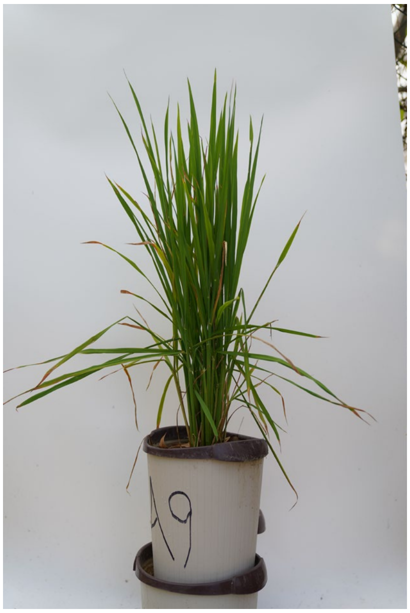

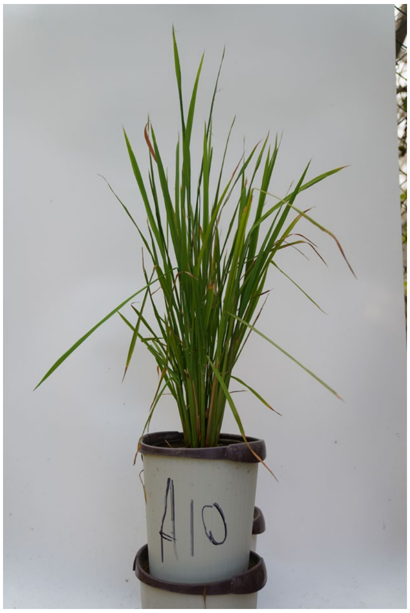

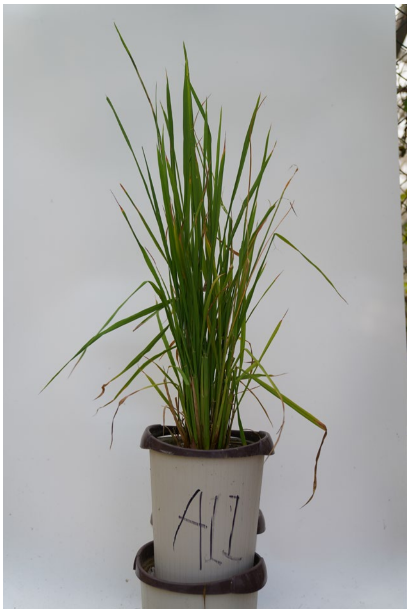

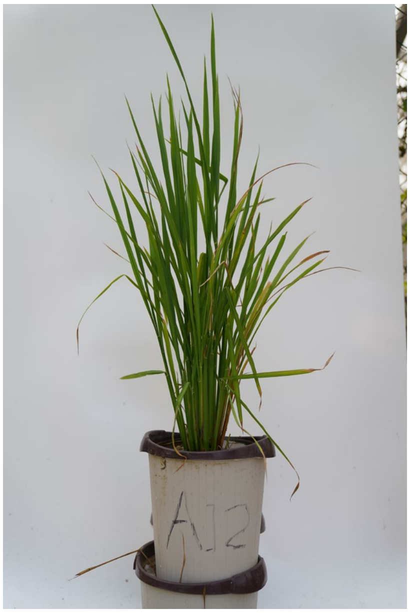

Sampling Date：2021-8-19　Growth Stage: Jointing

Experimental Group：WM

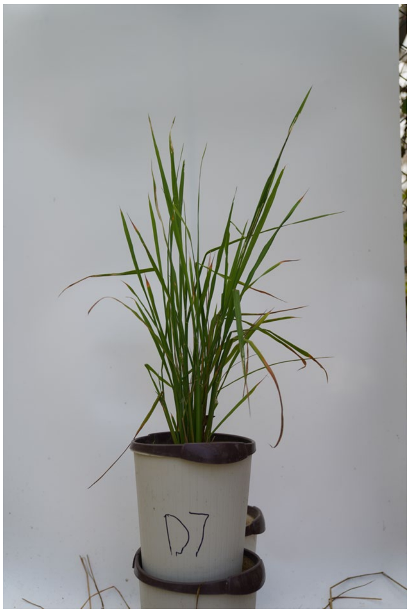

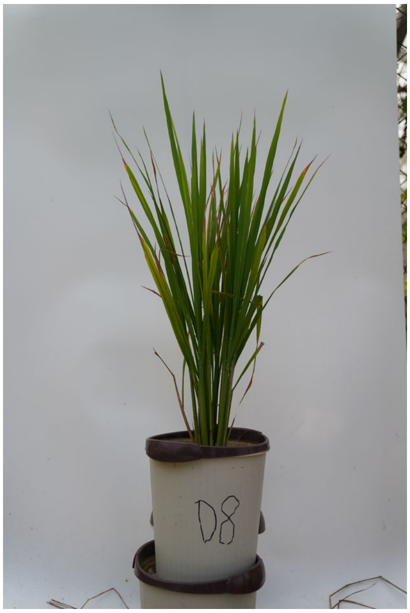

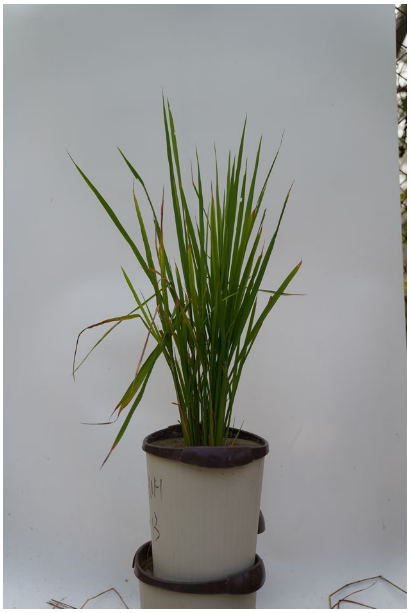

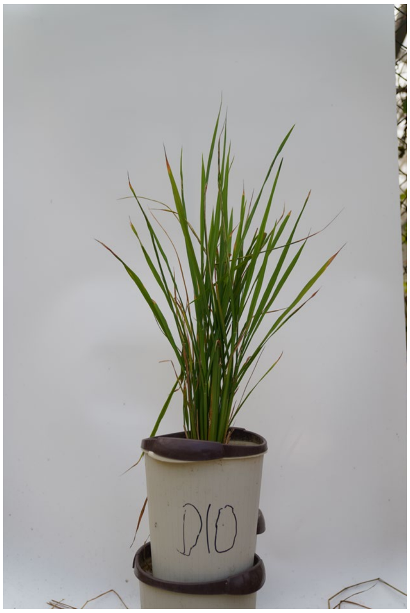

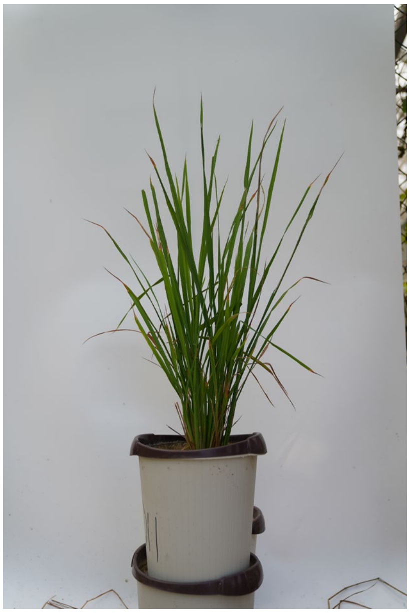

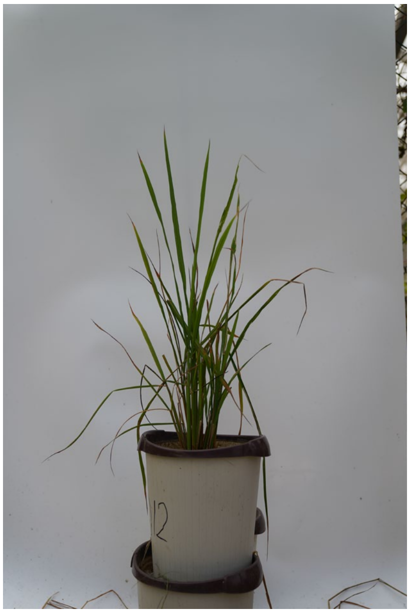

Sampling Date：2021-8-19　Growth Stage: Jointing

Experimental Group：WS

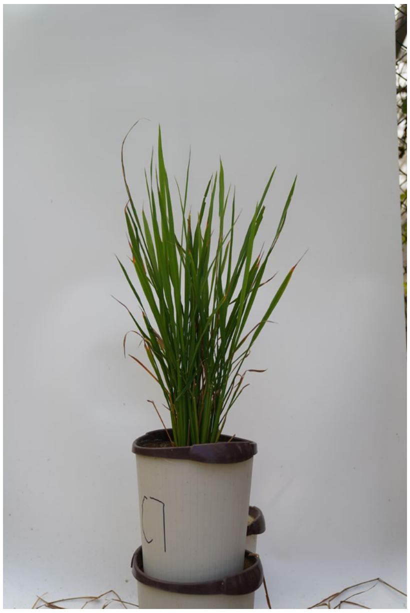

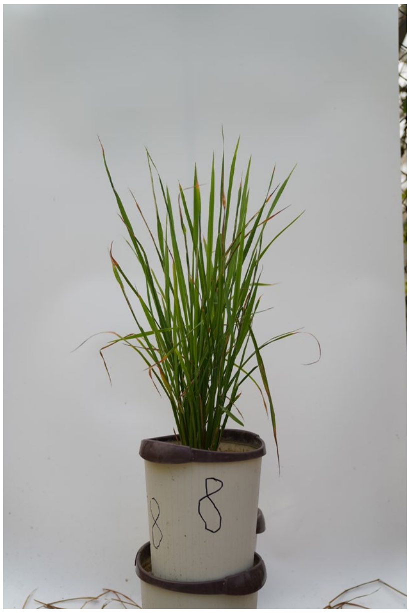

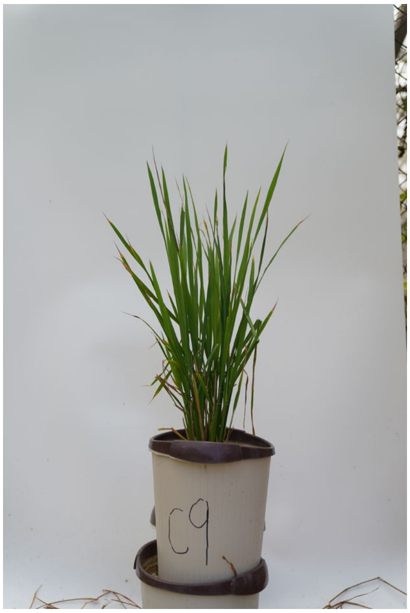

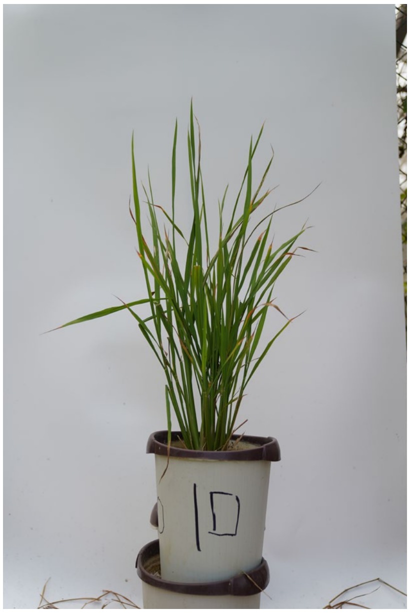

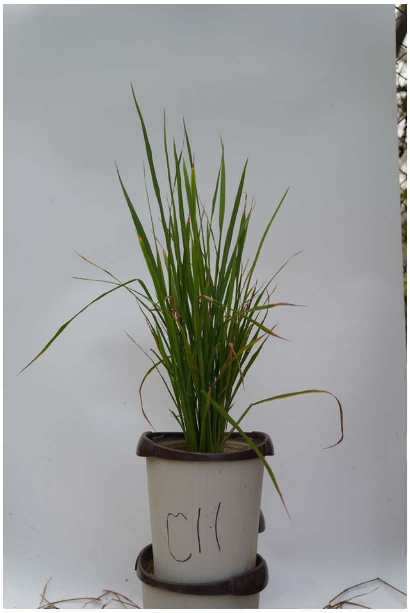

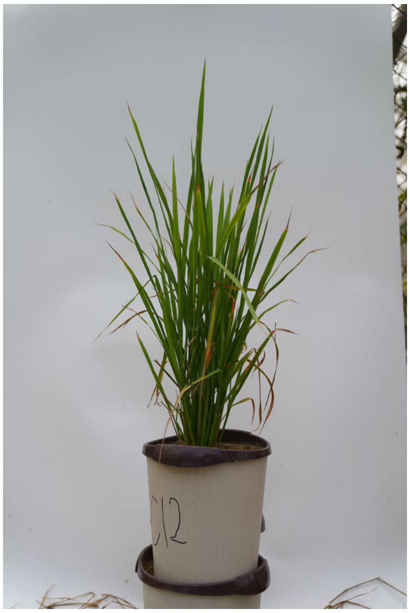

Sampling Date：2021-9-1　　Growth Stage: Heading

Experimental Group：WF

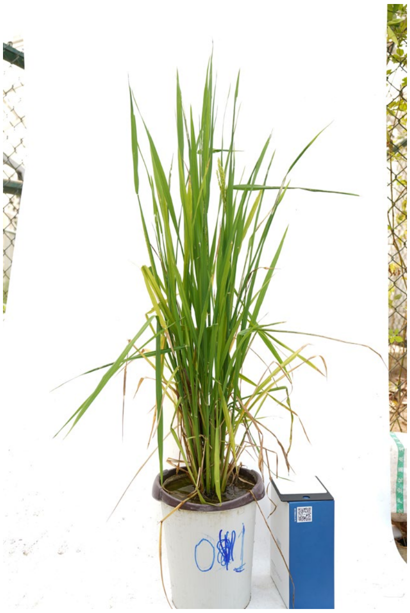
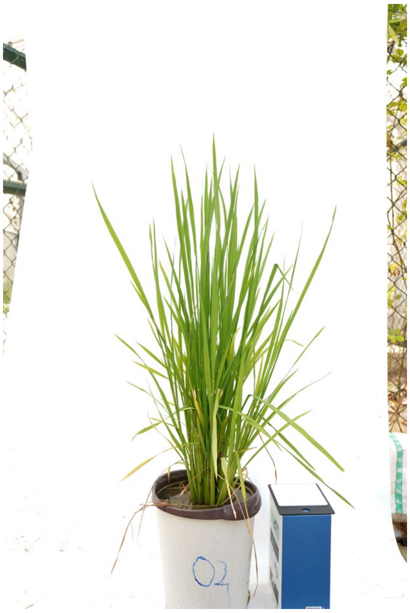
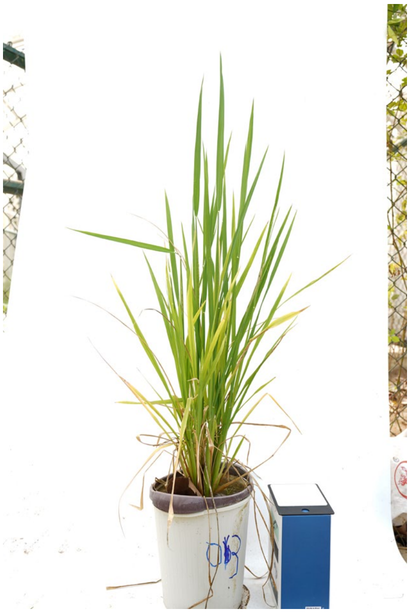
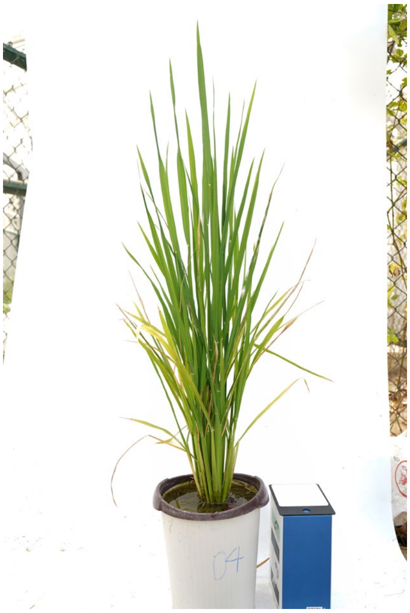
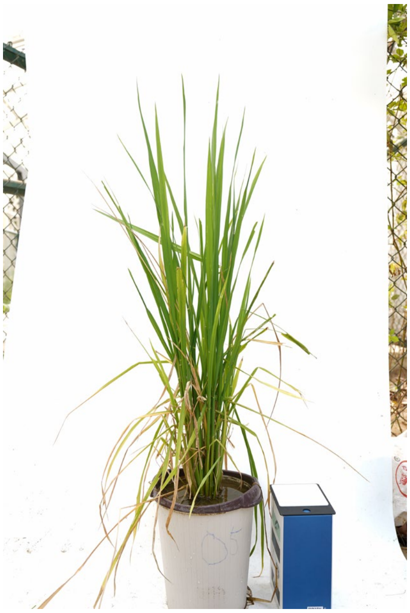
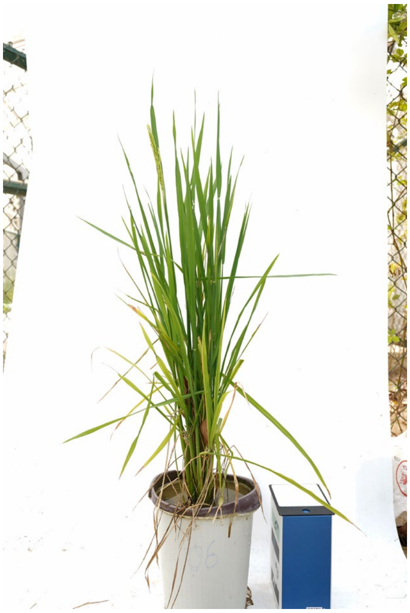

Sampling Date：2021-9-1　　Growth Stage: Heading

Experimental Group：HS

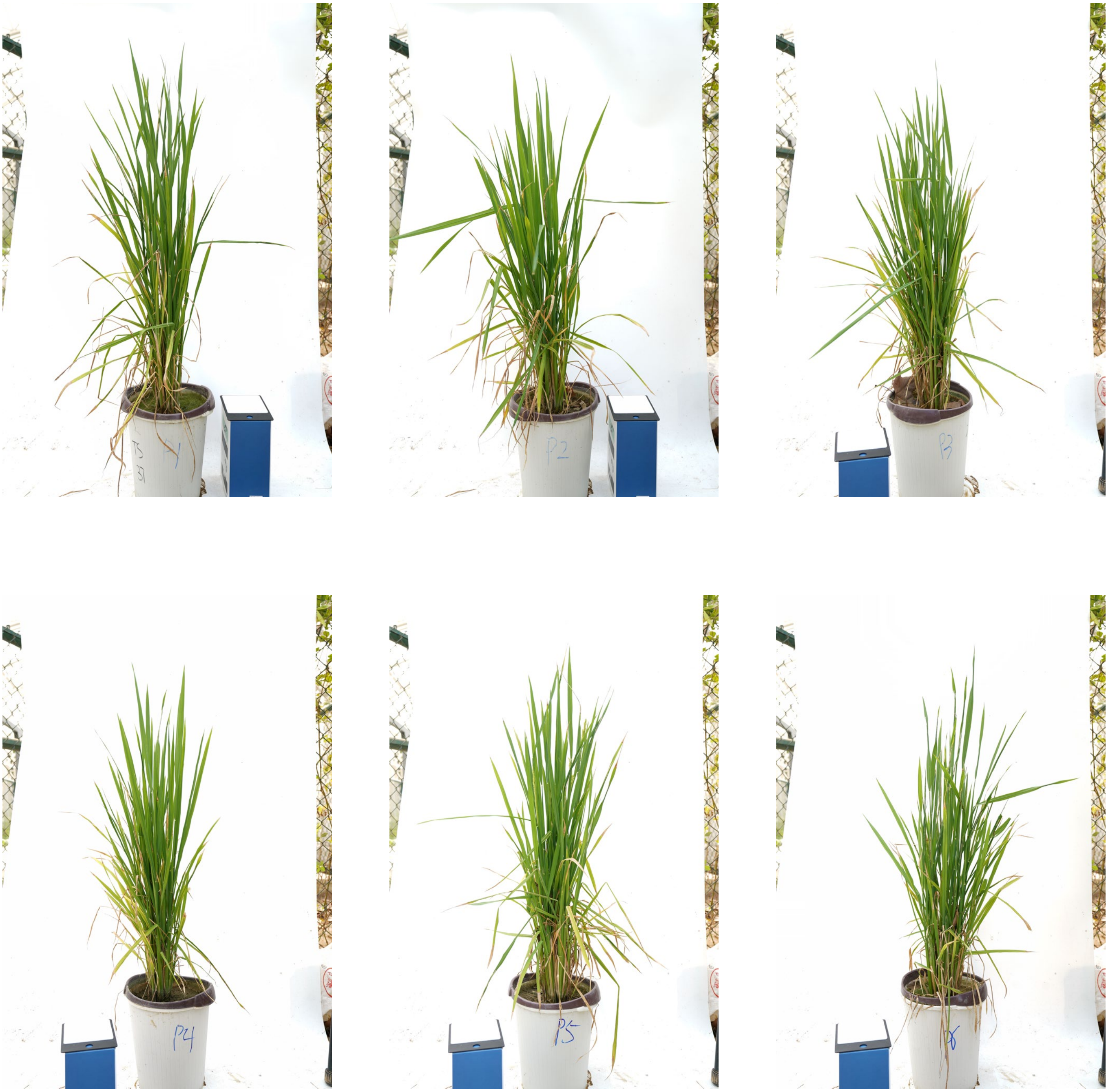

Sampling Date：2021-9-4   Growth Stage: Heading

Experimental Group：WF

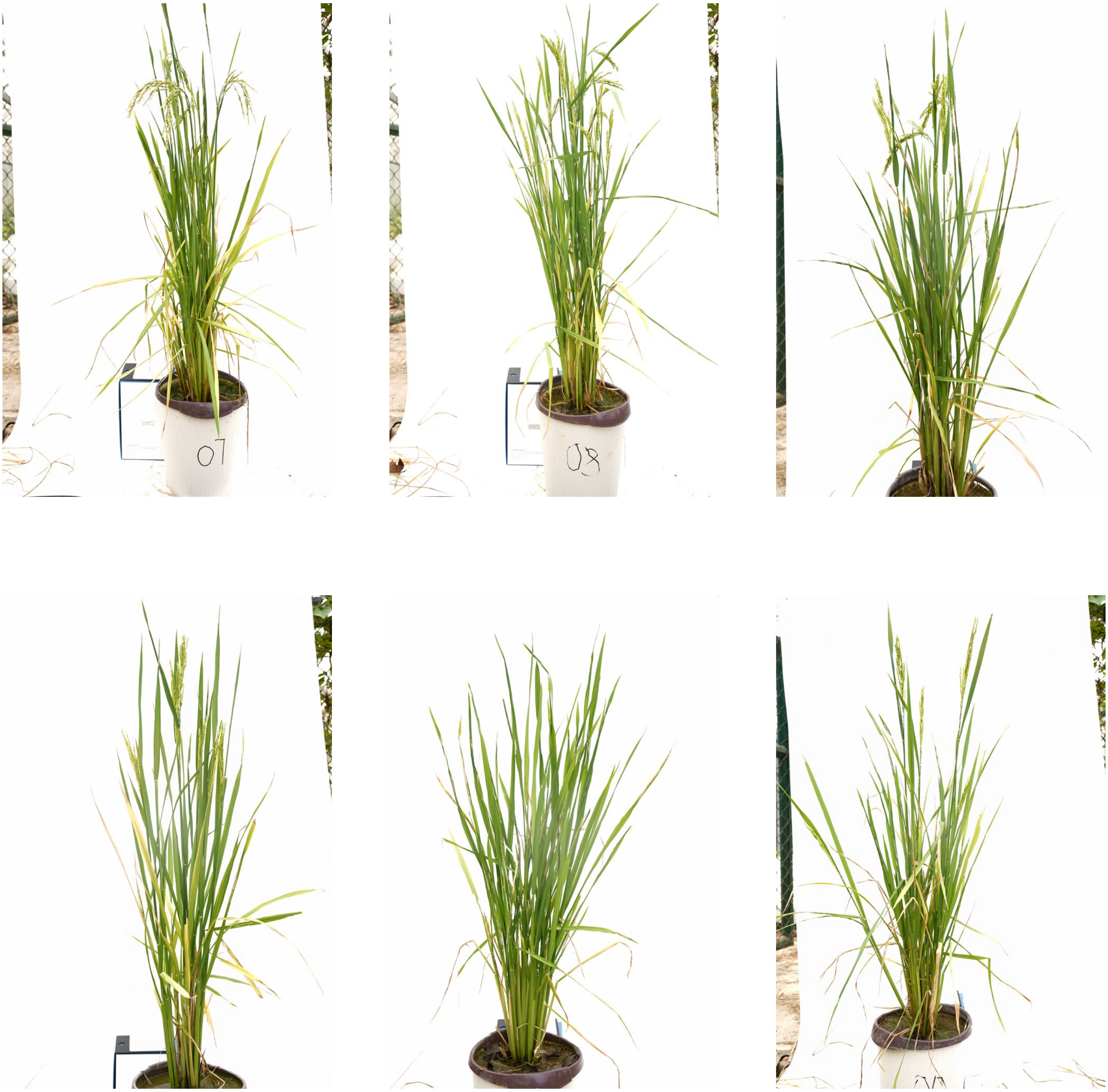

Sampling Date：2021-9-4 Growth Stage: Heading

Experimental Group：HS

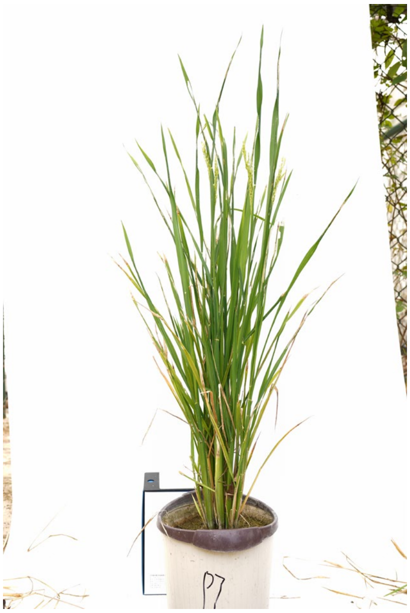

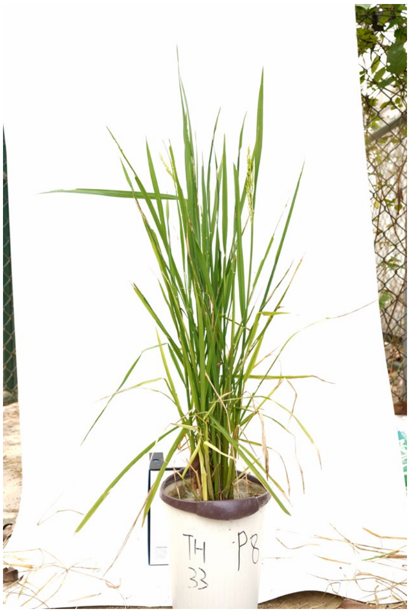

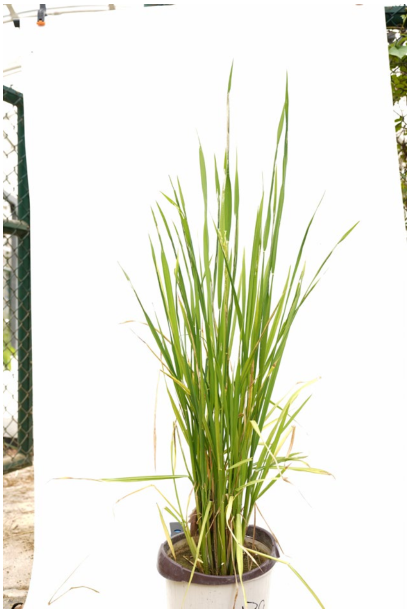

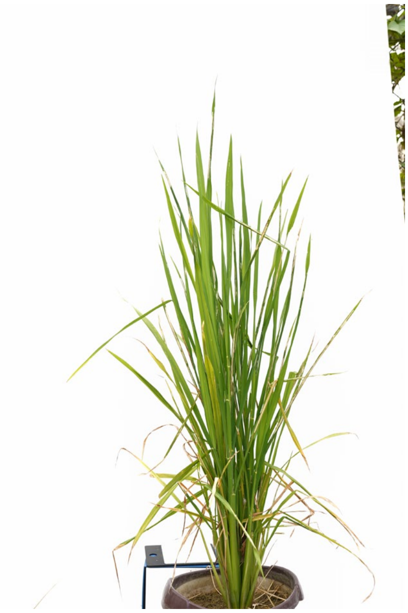

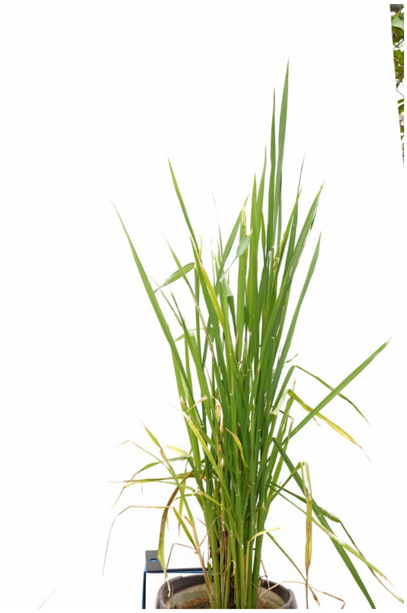

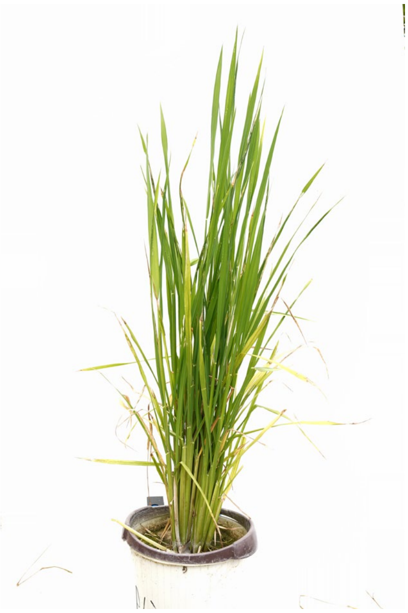

Sampling Date：2021-9-7　Growth Stage: Heading

Experimental Group：WF

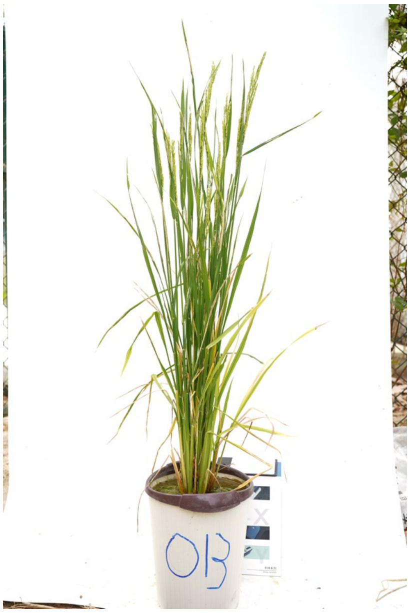

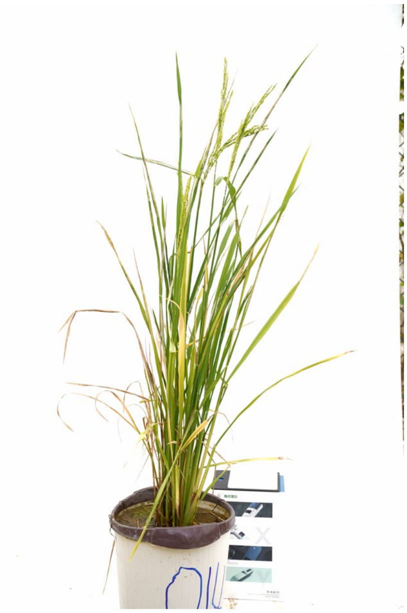

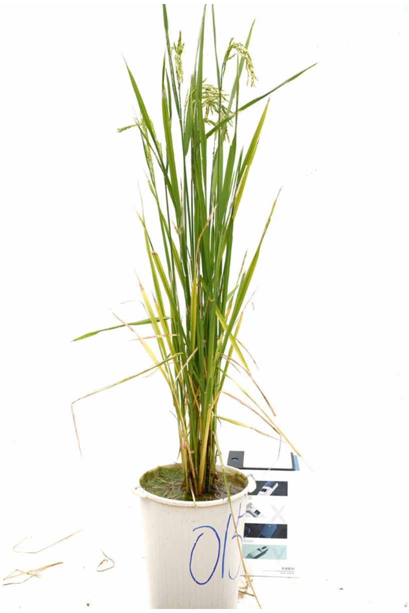

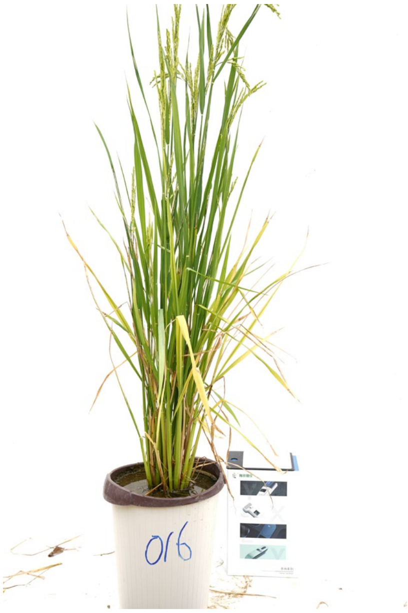

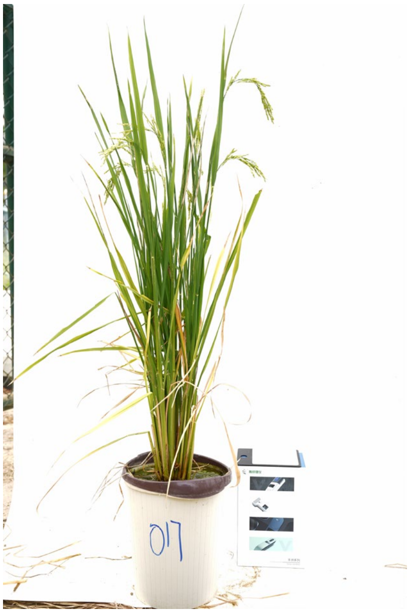

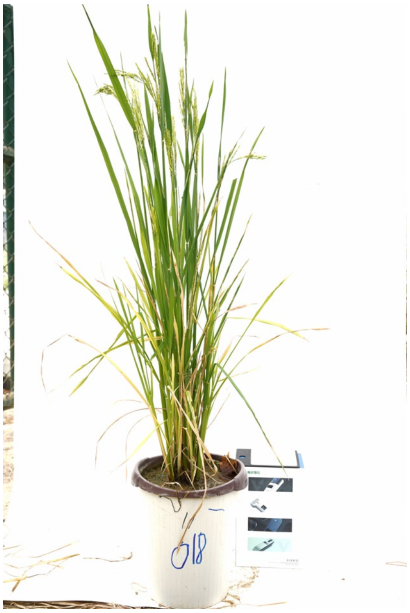

Sampling Date：2021-9-7 Growth Stage: Heading

Experimental Group：HS

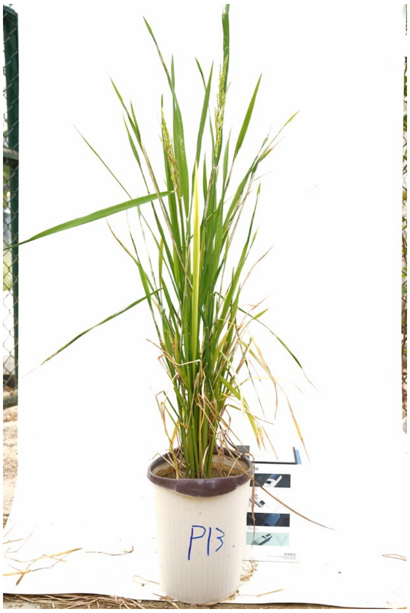

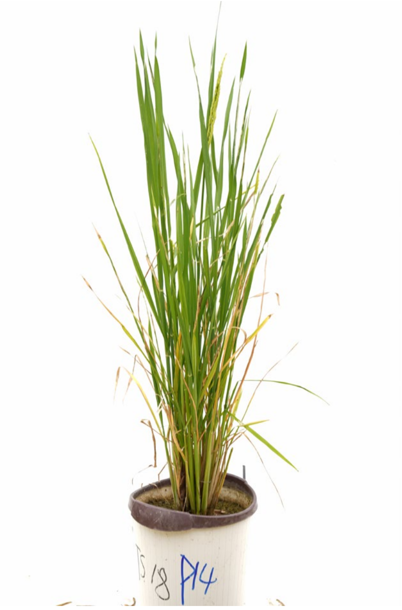

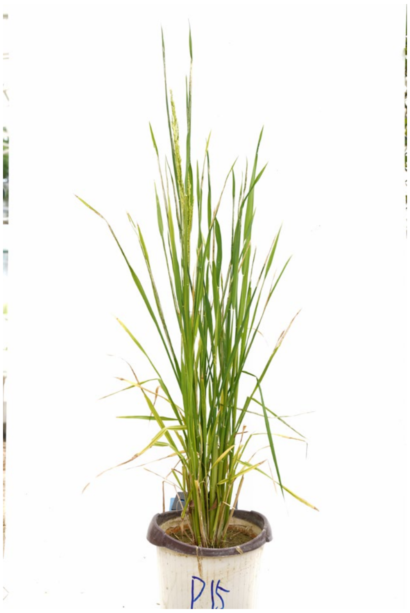

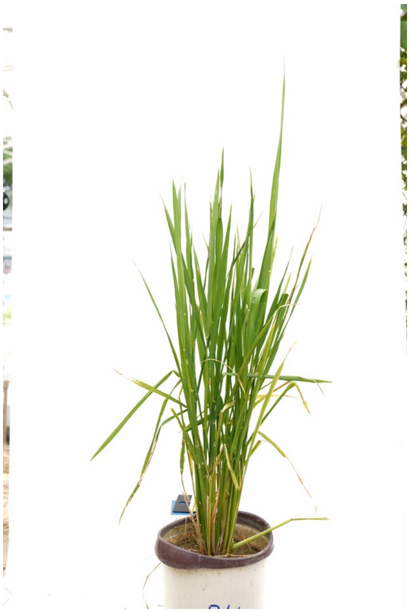

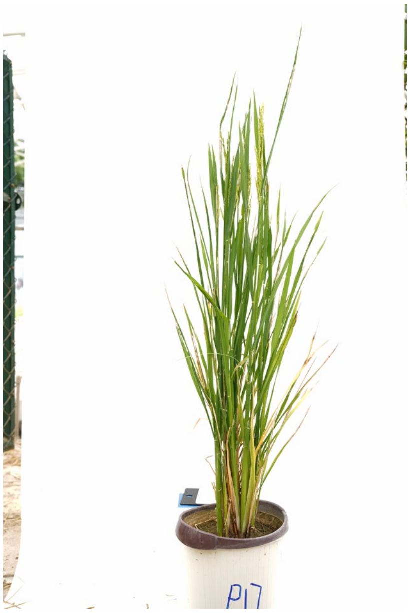

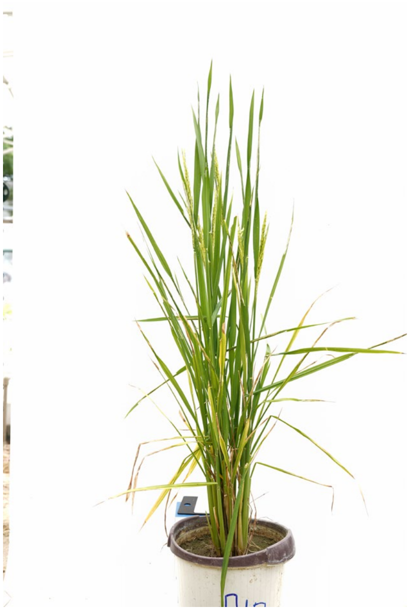

Sampling Date：2021-9-13　Growth Stage: Heading

Experimental Group：WF

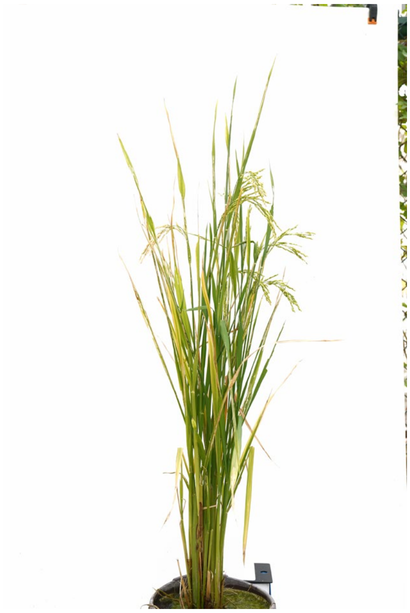
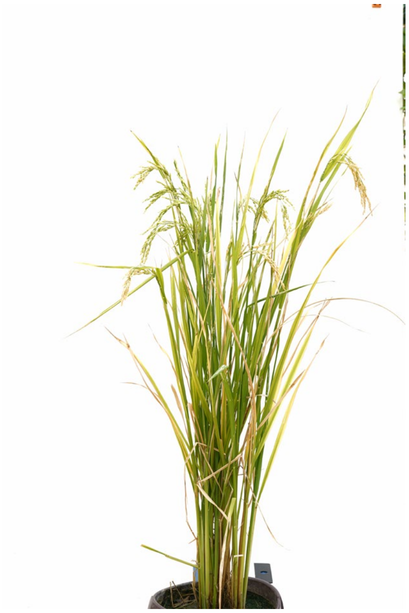
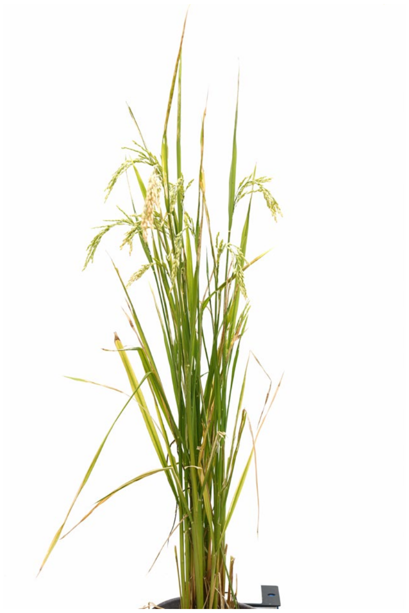
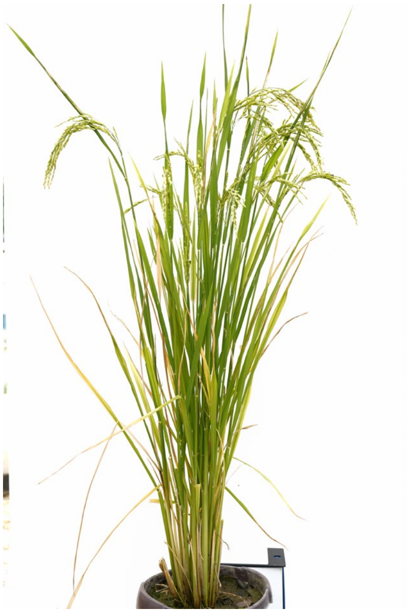
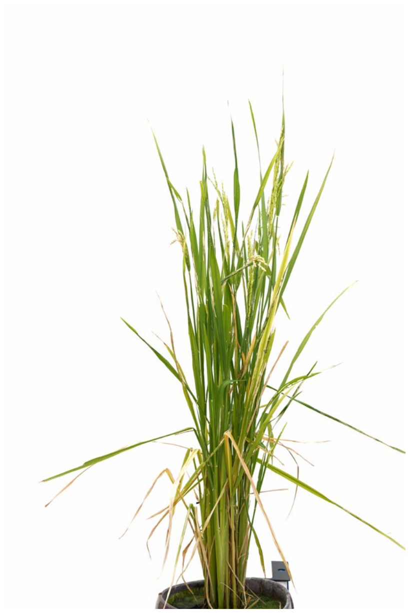
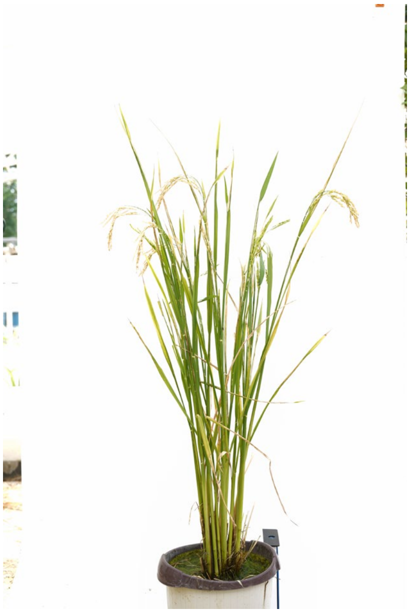

Sampling Date：2021-9-13 Growth Stage: Heading

Experimental Group：WM

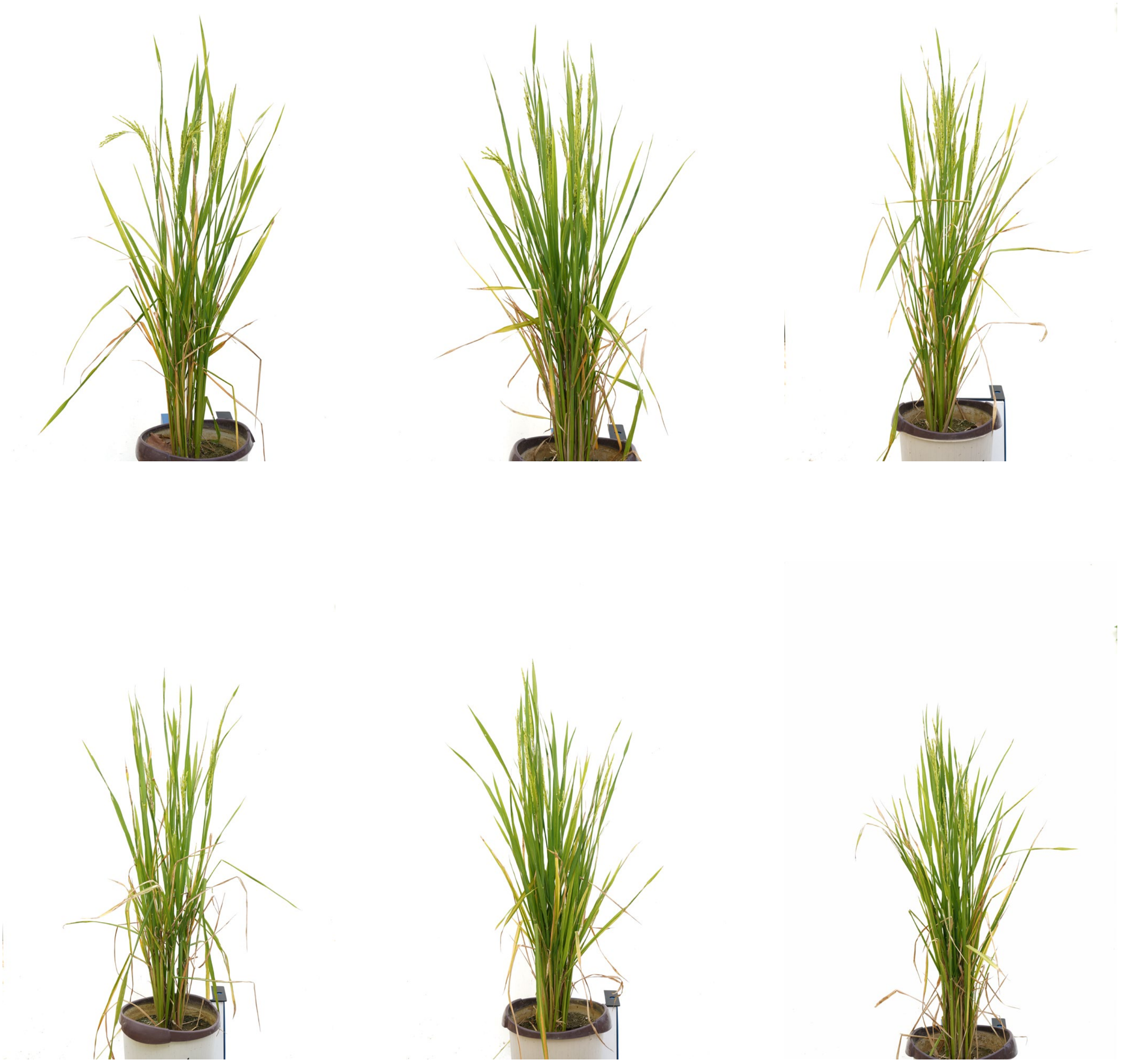

Sampling Date：2021-9-13 Growth Stage: Heading

Experimental Group：WS

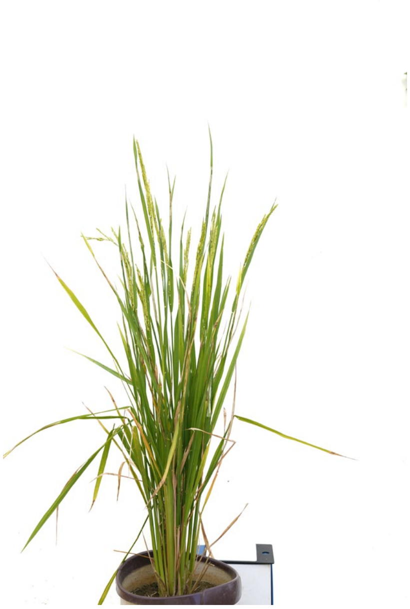
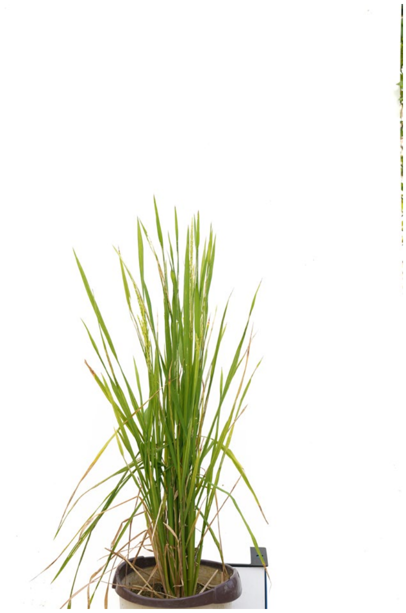
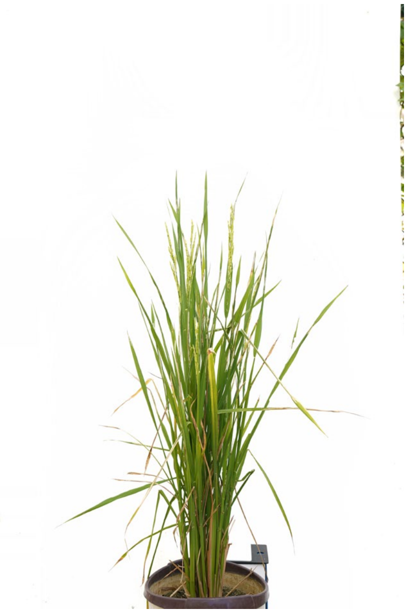
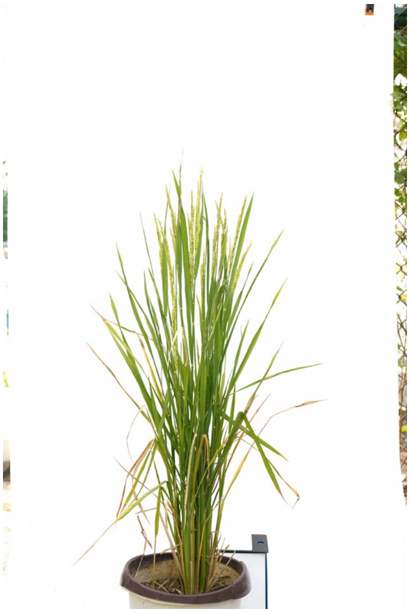
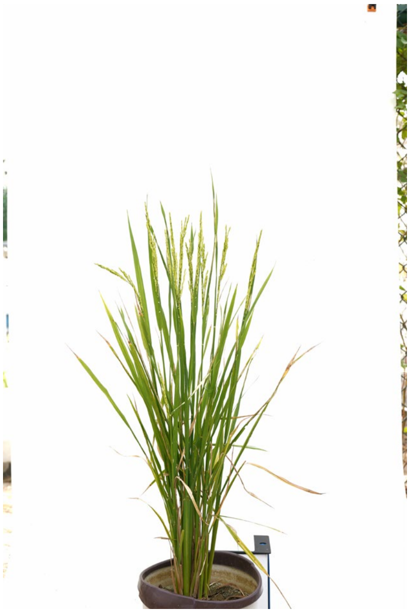
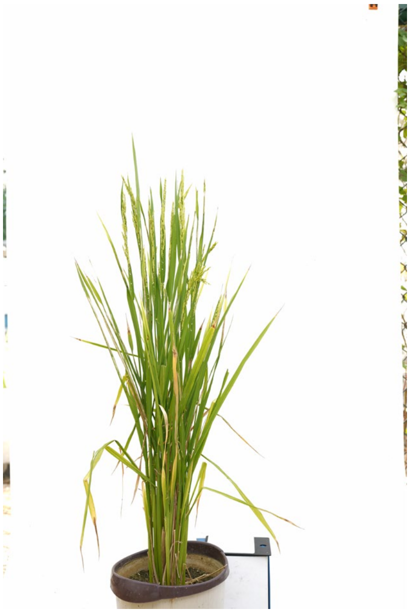

Sampling Date：2021-9-13　Growth Stage: Heading

Experimental Group：HS

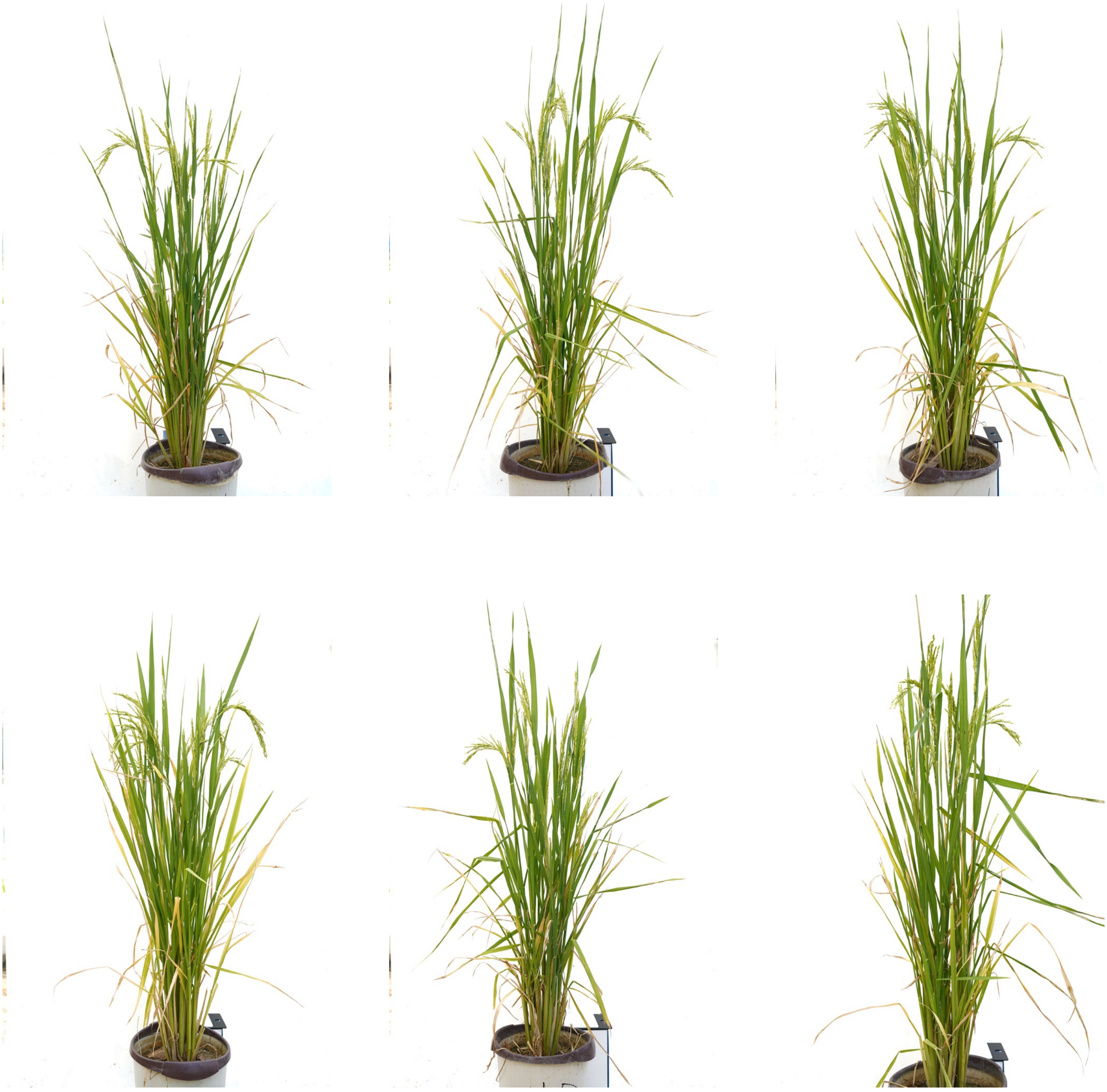

Sampling Date：2021-9-19　Growth Stage: Filling

Experimental Group：WF

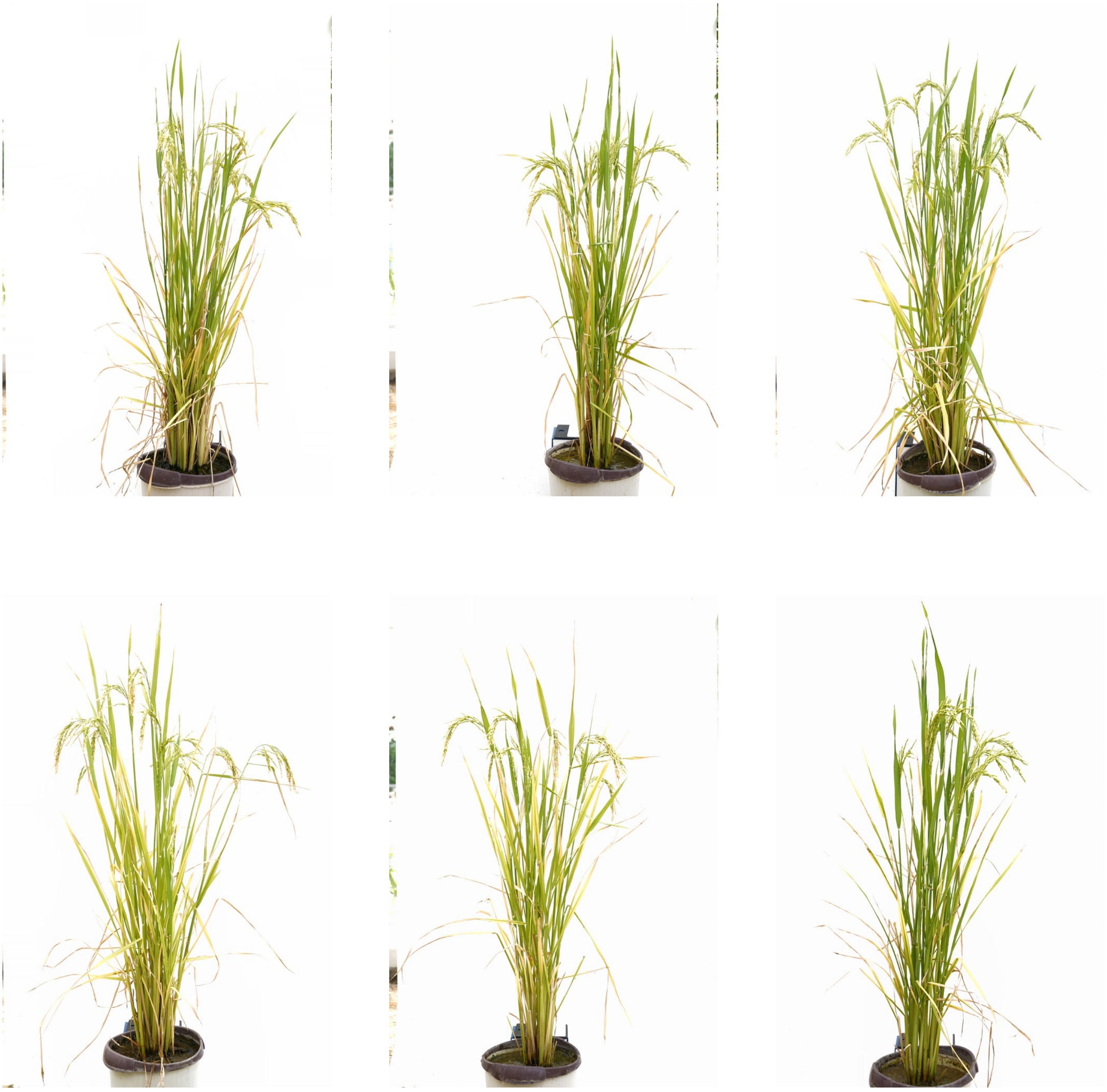

Sampling Date：2021-9-19   Growth Stage: Filling

Experimental Group：HS

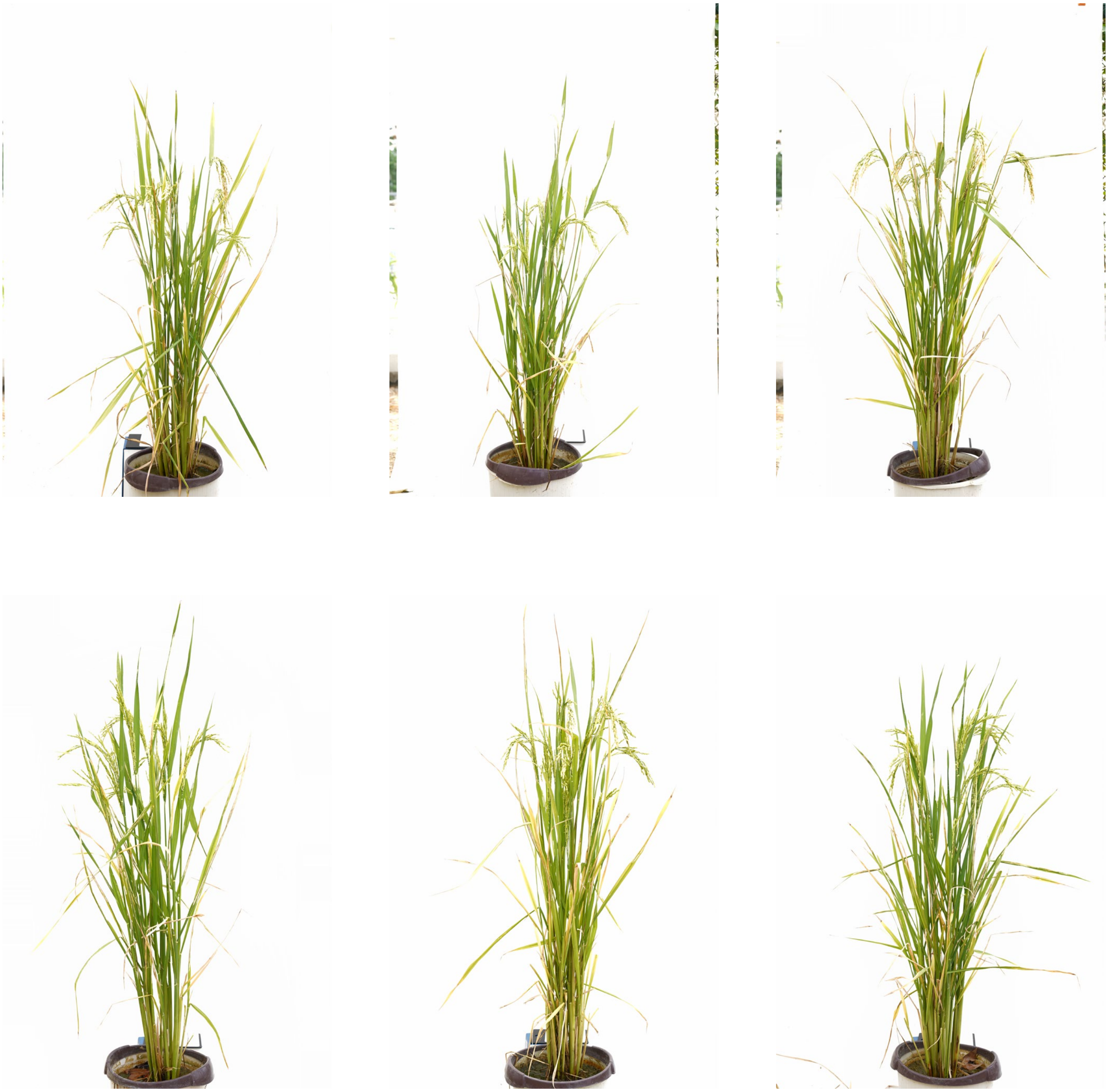

Sampling Date：2021-9-22　Growth Stage: Filling

Experimental Group：WF

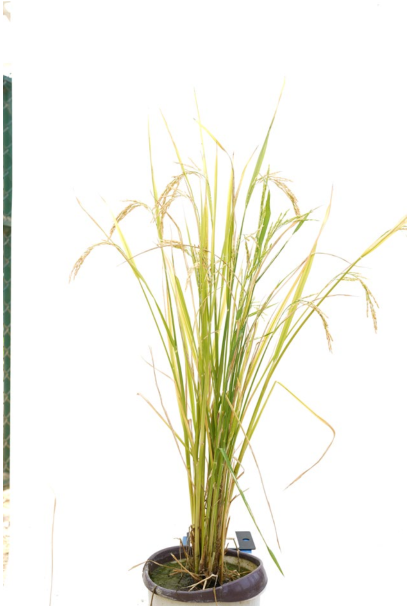
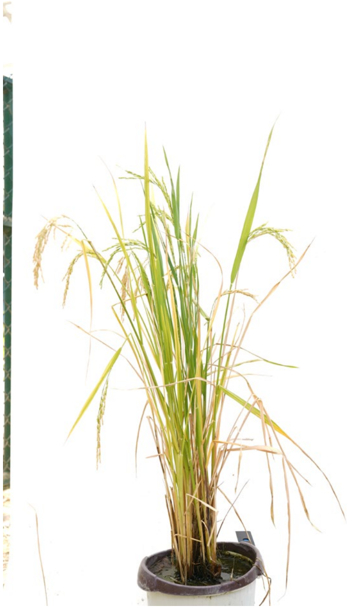
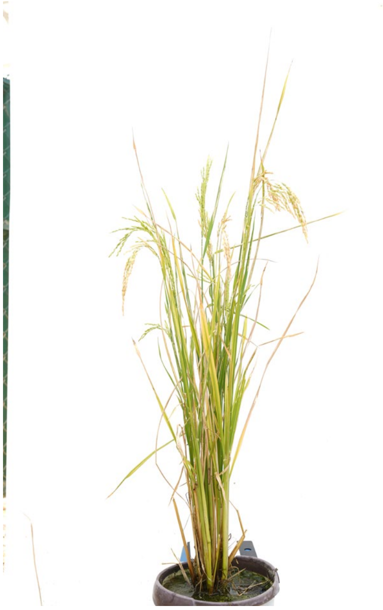
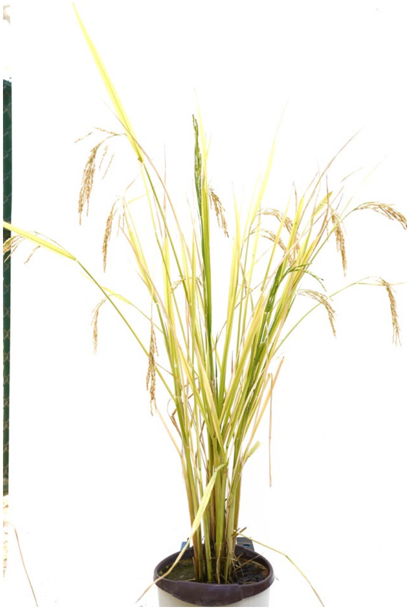
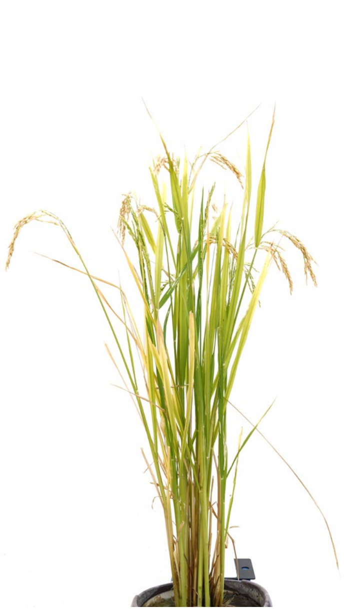
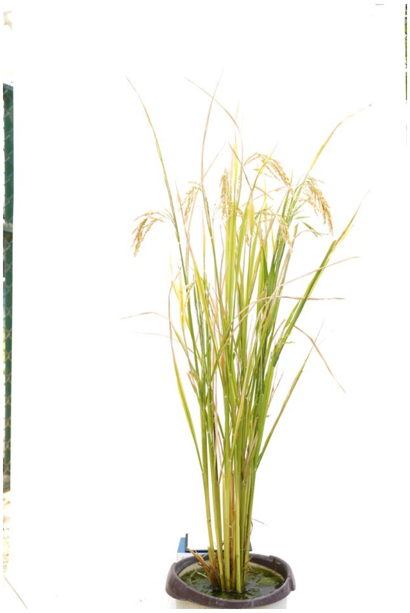

Sampling Date：2021-9-22 Growth Stage: Filling

Experimental Group：HS

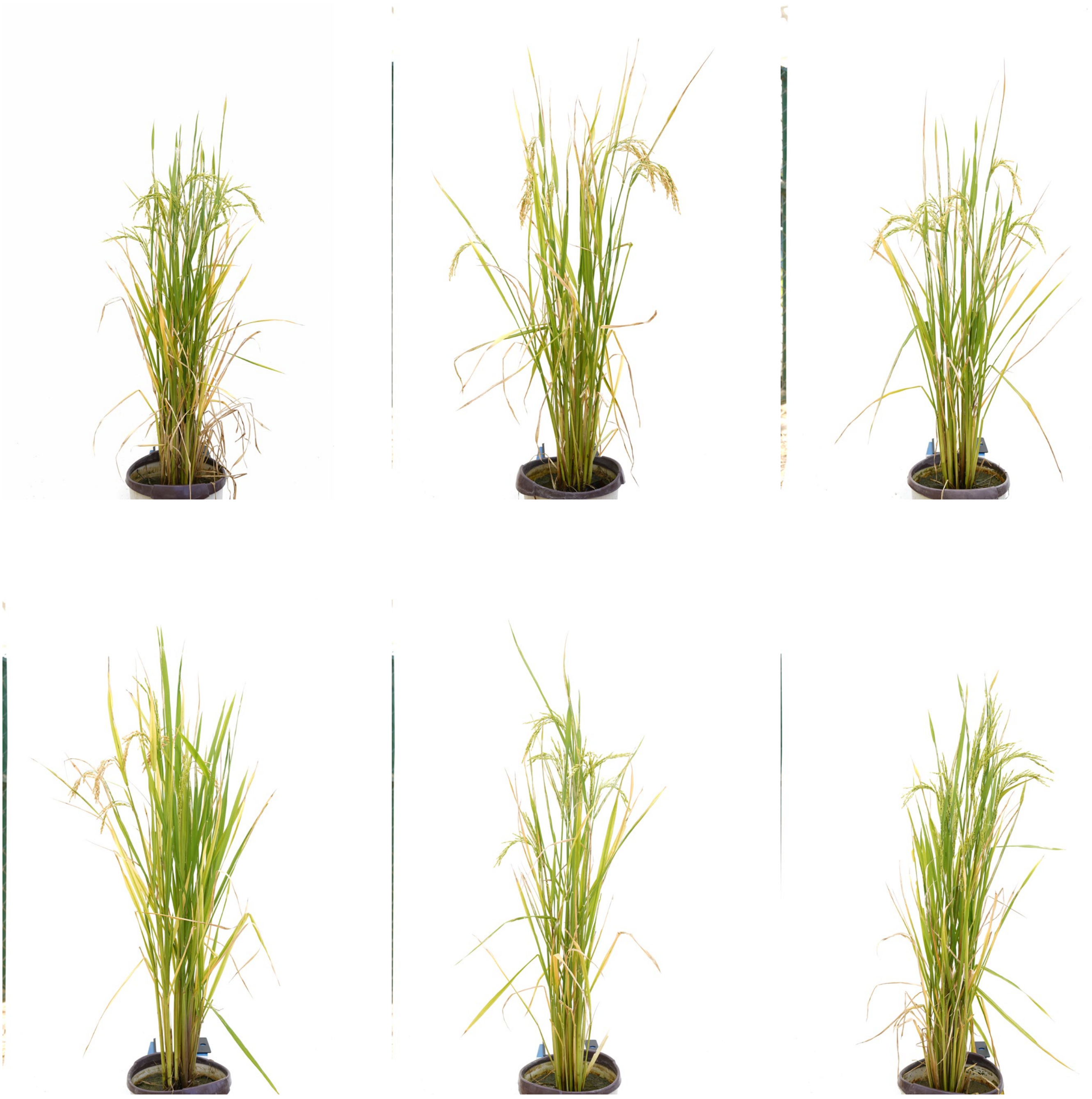

Sampling Date：2021-9-25　Growth Stage: Filling

Experimental Group：WF

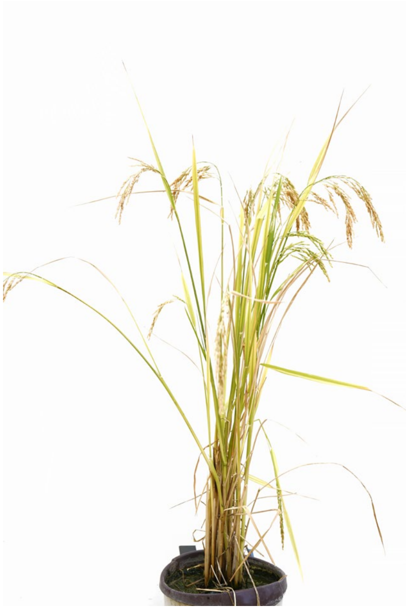
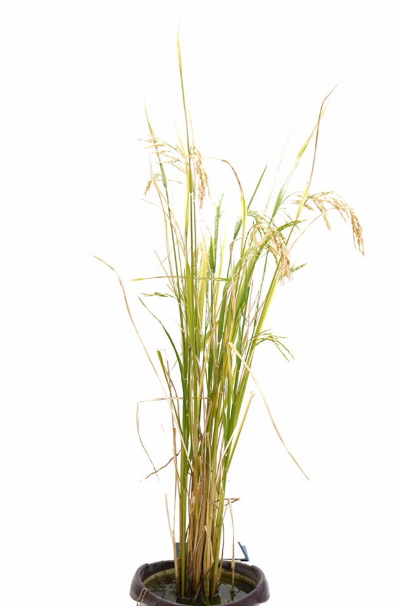
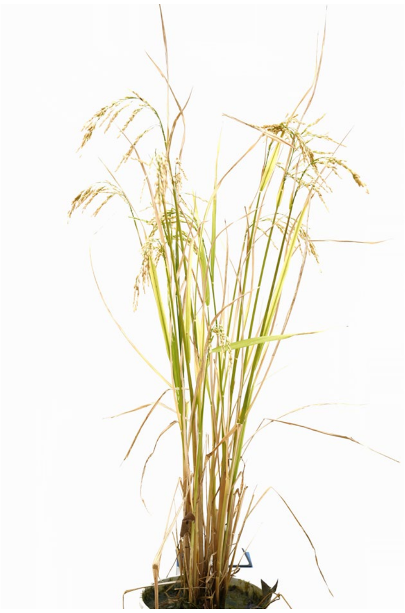
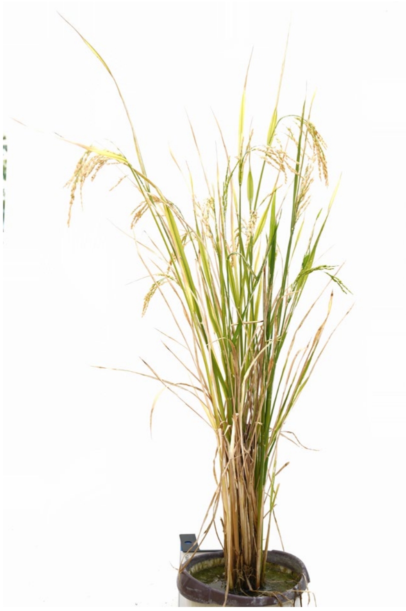
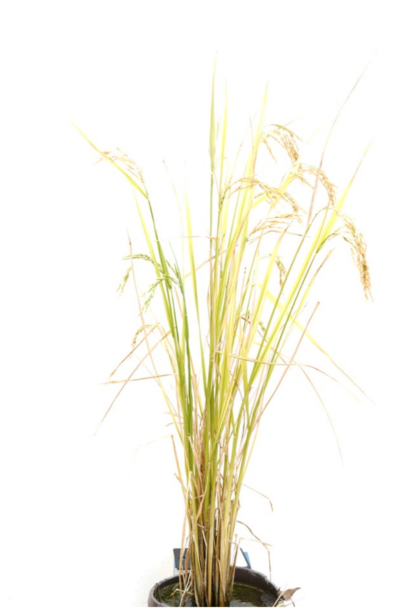
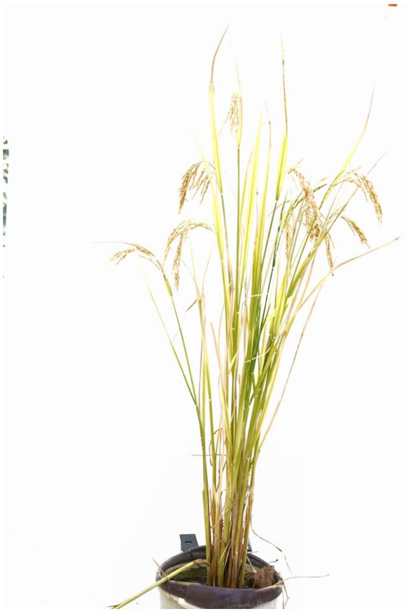

Sampling Date：2021-9-25   Growth Stage: Filling

Experimental Group：HS

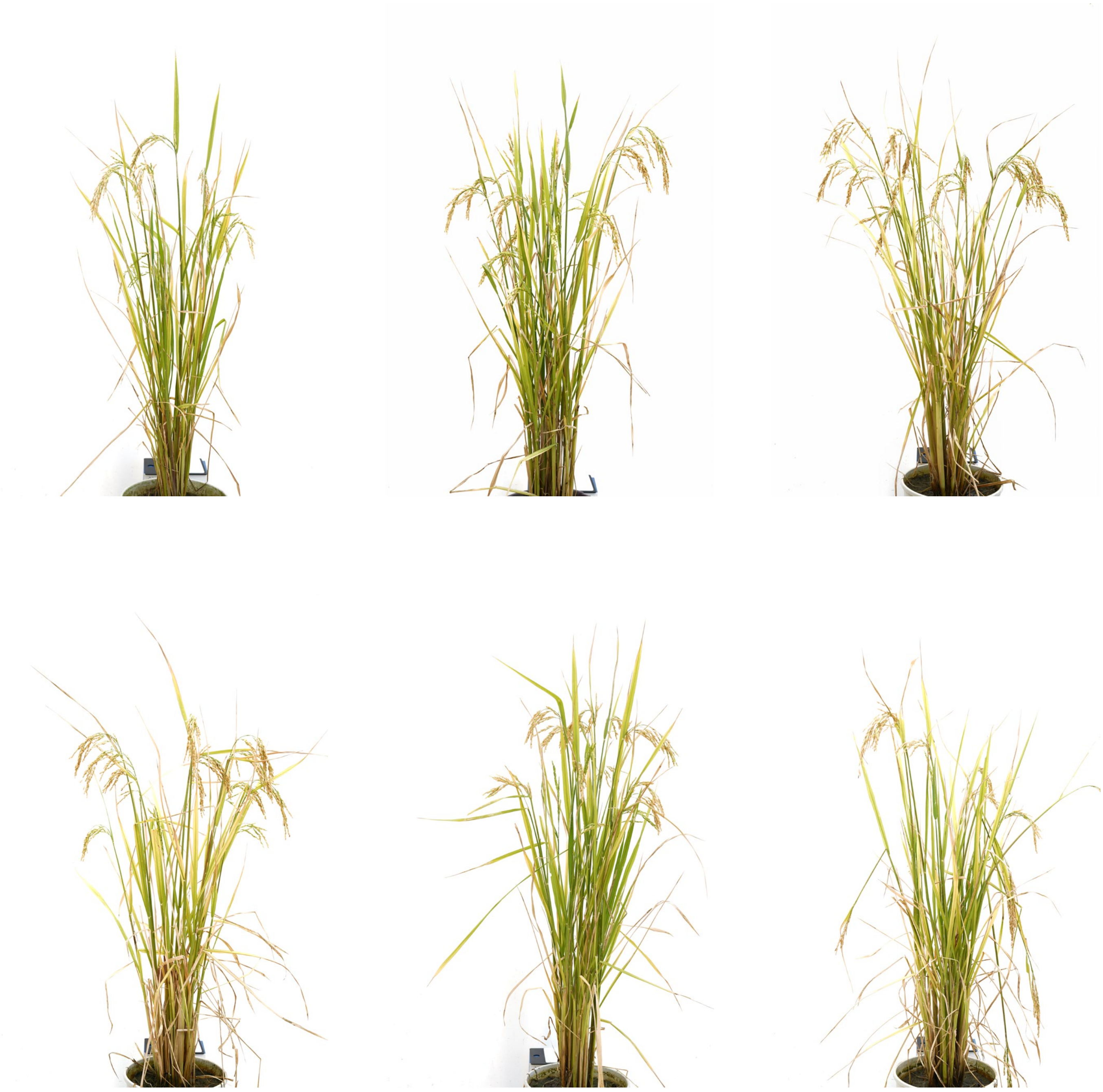

Sampling Date：2021-10-2 Growth Stage: Milk-ripeness

Experimental Group：WF

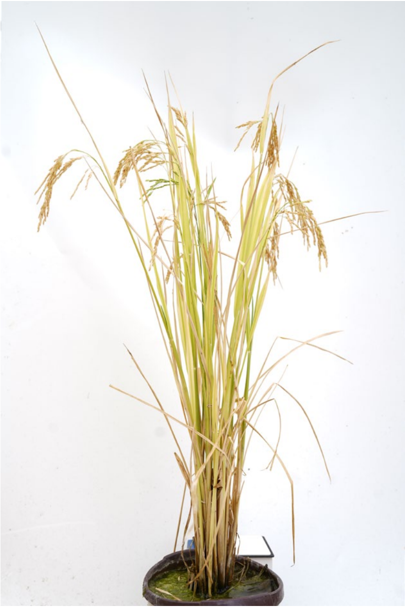
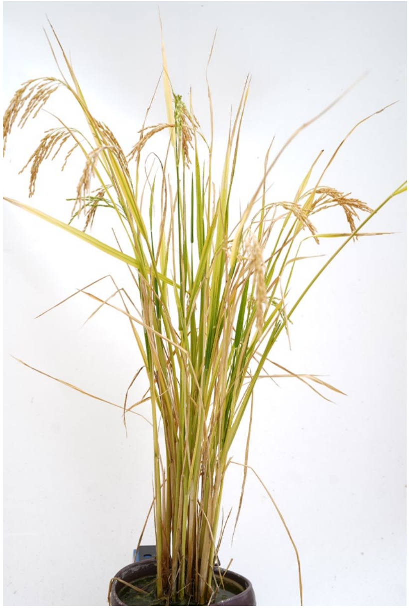
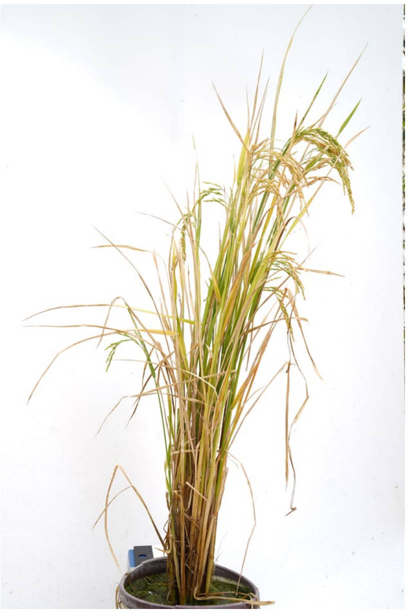
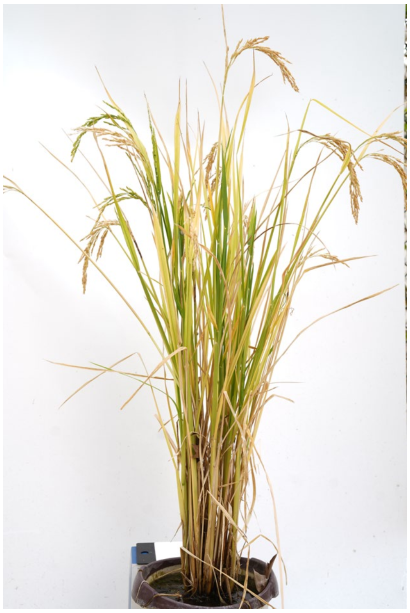
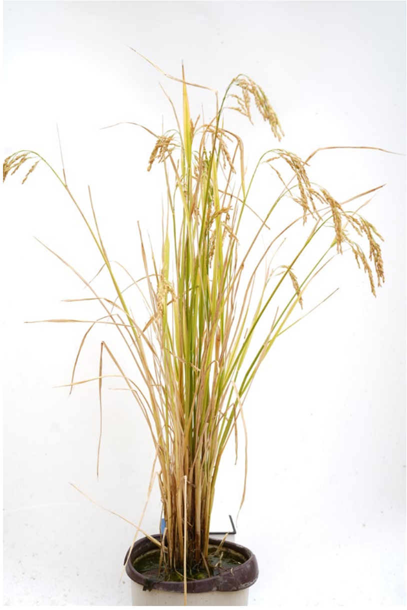
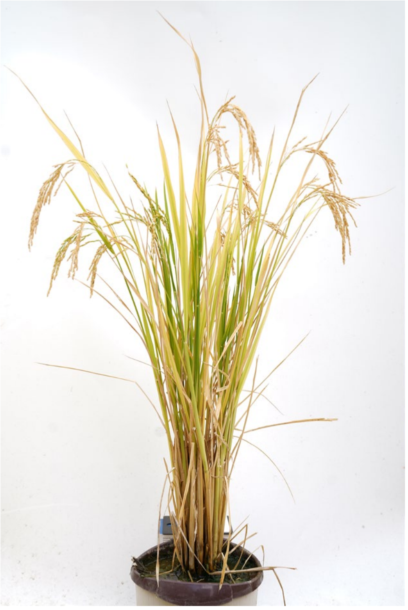

Sampling Date：2021-10-2　Growth Stage: Milk-ripeness

Experimental Group：WM

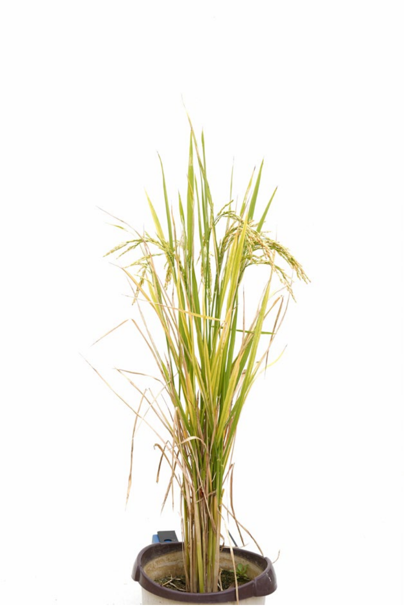
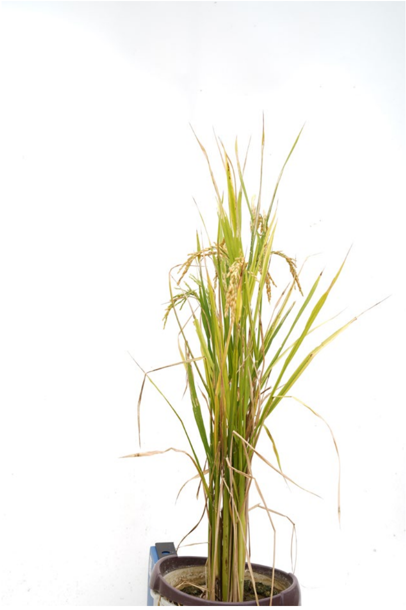
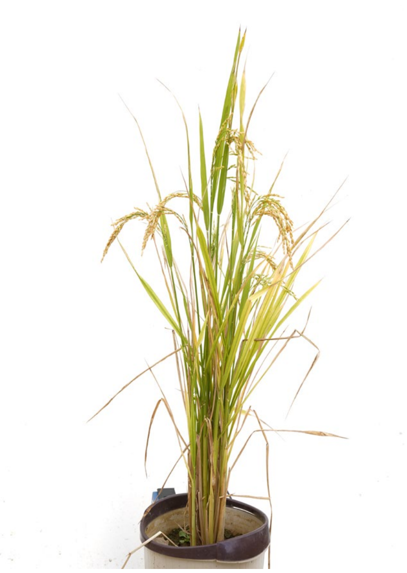

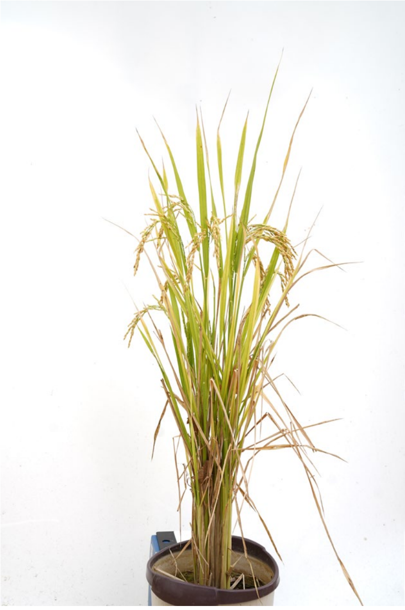
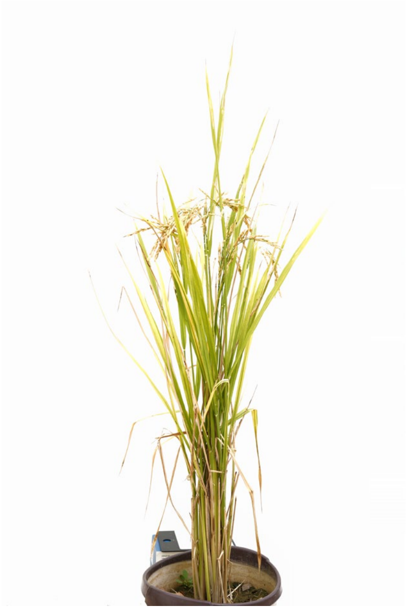
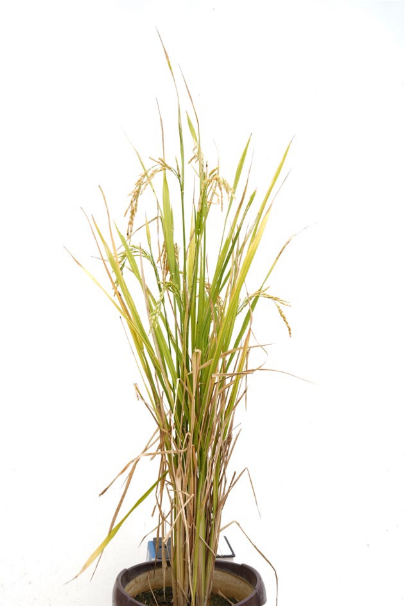

Sampling Date：2021-10-2　Growth Stage: Milk-ripeness

Experimental Group：WS

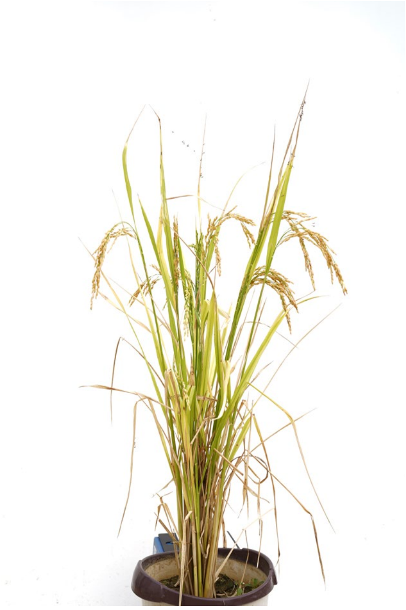
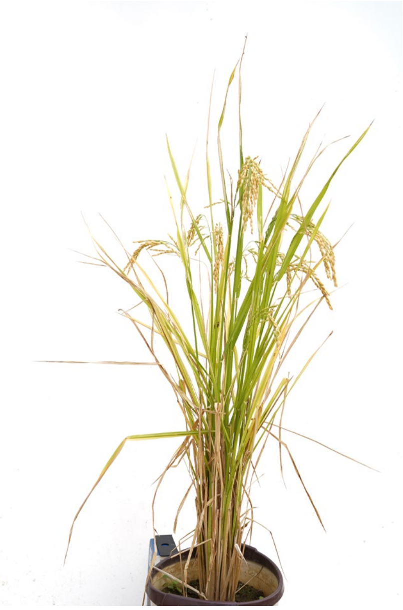
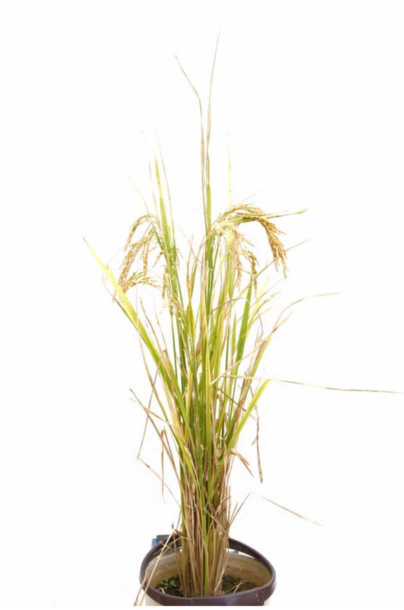
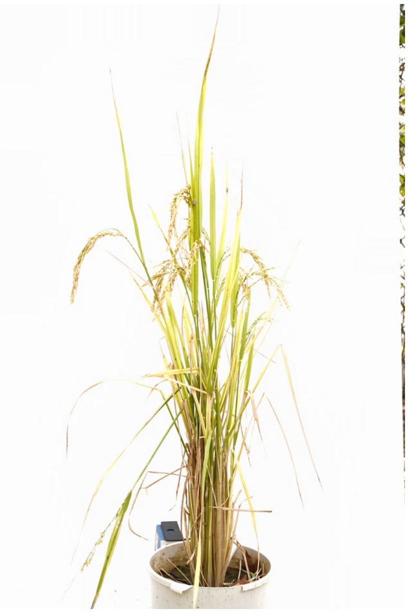
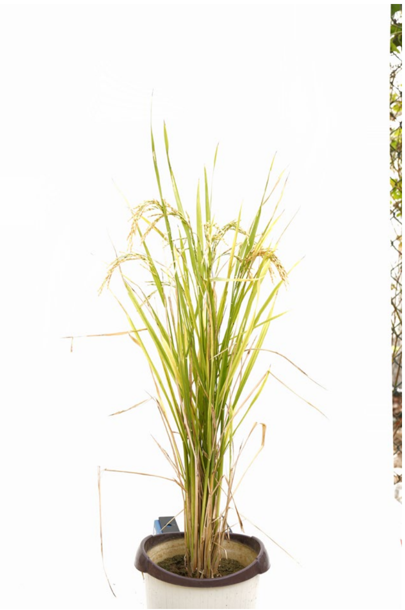
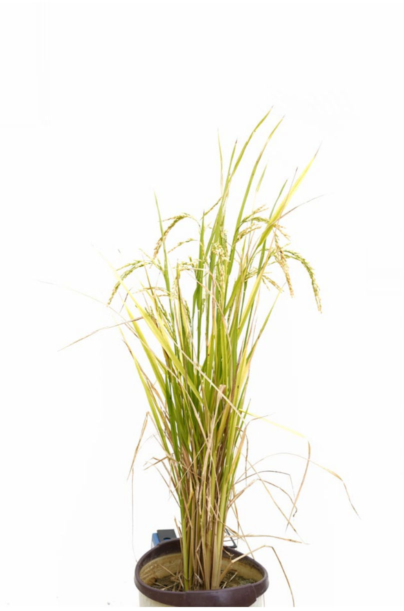

Sampling Date：2021-10-2　Growth Stage: Milk-ripeness

Experimental Group：HS

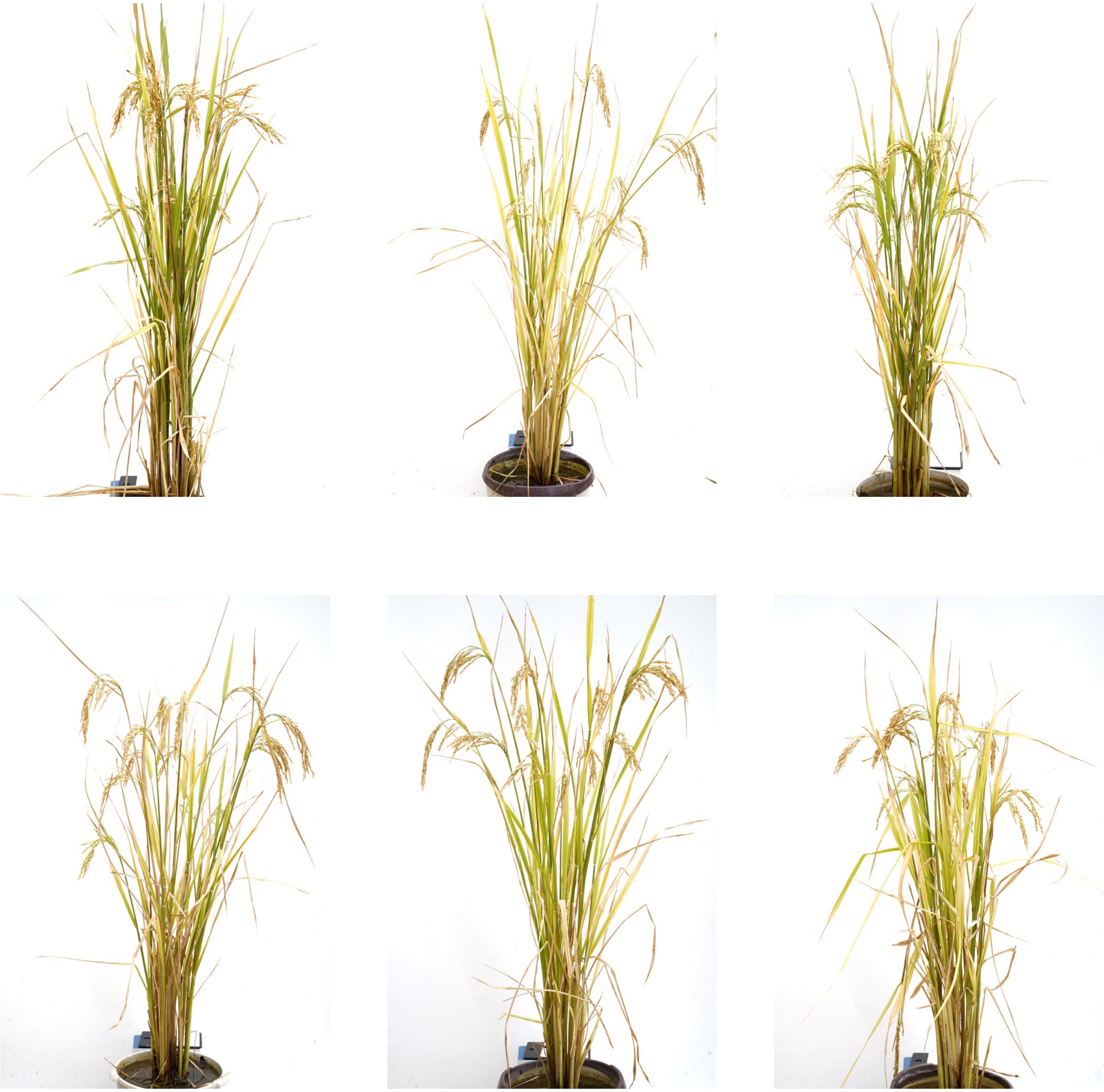

Supplement: Supplementary file 2 [file Data_Sheet_2.PDF]
